# Supplementary material for: Polyketide synthase gene clusters in the ericoid mycorrhizal fungus Oidiodendron maius: genome-wide prediction, expression profiling, and functional characterization of knockout mutants
Source: Front Plant Sci. 2026 Sep 16;17:1924430. doi: 10.3389/fpls.2026.1924430 (PMC13623903; doi:10.3389/fpls.2026.1924430)
Supplement: Supplementary file 1 [file SupplementaryFile1.zip › Supplementary Materials/Supplementary Folder 1.zip!/Oidma1_AssemblyScaffolds_Repeatmasked/knownclusterblast/cluster53/jgi.p_Oidma1_173316_mibig_hits.html]

| MIBiG Protein | Description | MIBiG Cluster | MiBiG Product | % ID | % Coverage | BLAST Score | E-value |
| --- | --- | --- | --- | --- | --- | --- | --- |
| ADH04658.1 | TugB | BGC0001342 | Thuggacin\_biosynthetic\_gene\_cluster | 43 | 60.9951845907 | 1090 | 0.0 |
| ADH04639.1 | TgaA | BGC0001051 | Thuggacin\_biosynthetic\_gene\_cluster | 41 | 66.3322632424 | 1071 | 1.2e-312 |
| BAH02271.1 | polyketide\_synthase | BGC0000126 | Pladienolide\_biosynthetic\_gene\_cluster | 41 | 60.9951845907 | 1008 | 1.3e-293 |
| ADC79637.1 | TamAI | BGC0001052 | Tirandamycin\_biosynthetic\_gene\_cluster | 40 | 64.2455858748 | 986 | 6.7e-287 |
| ANI24099.1 | polyketide\_synthase | BGC0001235 | Antalid\_biosynthetic\_gene\_cluster | 40 | 64.6468699839 | 983 | 5.7e-286 |
| BAB69192.1 | modular\_polyketide\_synthase | BGC0000117 | Oligomycin\_biosynthetic\_gene\_cluster | 39 | 65.3691813804 | 970 | 3.8e-282 |
| AEU17899.1 | putative\_type\_I\_PKS | BGC0001072 | Simocyclinone\_biosynthetic\_gene\_cluster | 39 | 64.9678972713 | 966 | 5.5e-281 |
| CUW01175.1 | Type\_I\_polyketide\_synthase | BGC0001359 | PM100117\_/\_PM100118\_biosynthetic\_gene\_cluster | 39 | 64.7672552167 | 965 | 1.2e-280 |
| AHH99922.1 | PKS\_I | BGC0000002 | Aculeximycin\_biosynthetic\_gene\_cluster | 39 | 63.6436597111 | 965 | 1.6e-280 |
| ADC45534.1 | modular\_polyketide\_synthase | BGC0000093 | Meilingmycin\_biosynthetic\_gene\_cluster | 37 | 65.4895666132 | 941 | 1.5e-273 |
| CUW01176.1 | Type\_I\_polyketide\_synthase | BGC0001359\_c2 | PM100117\_/\_PM100118\_biosynthetic\_gene\_cluster | 40 | 61.1958266453 | 938 | 2.1e-272 |
| AAK73501.1 | AmphI | BGC0000015 | Amphotericin\_biosynthetic\_gene\_cluster | 38 | 65.1685393258 | 935 | 1.4e-271 |
| ADC79616.1 | BafAI | BGC0000028 | Bafilomycin\_biosynthetic\_gene\_cluster | 37 | 67.6565008026 | 928 | 1.7e-269 |
| ABC87511.1 | polyketide\_synthase | BGC0001011 | Meridamycin\_biosynthetic\_gene\_cluster | 39 | 64.3258426966 | 926 | 4.8e-269 |
| ctg1\_orf22 |  | BGC0001013 | Meridamycin\_biosynthetic\_gene\_cluster | 39 | 64.3258426966 | 926 | 4.8e-269 |
| ADC45516.1 | modular\_polyketide\_synthase | BGC0000093 | Meilingmycin\_biosynthetic\_gene\_cluster | 39 | 64.7672552167 | 920 | 5.9e-267 |
| AAB66508.1 | tylactone\_synthase\_module\_7 | BGC0000166 | Tylactone\_biosynthetic\_gene\_cluster | 40 | 61.3563402889 | 919 | 1e-266 |
| CAL58684.1 | polyketide\_synthase | BGC0000149 | Spirangien\_biosynthetic\_gene\_cluster | 38 | 64.3659711075 | 916 | 6.6e-266 |
| AAF71766.1 | NysI | BGC0000115 | Nystatin\_biosynthetic\_gene\_cluster | 38 | 64.0048154093 | 908 | 1.8e-263 |
| ACY06287.1 | type\_I\_polyketide\_synthase | BGC0001042 | Sanglifehrin\_A\_biosynthetic\_gene\_cluster | 38 | 66.1717495987 | 907 | 4e-263 |
| AHH99925.1 | PKS\_I | BGC0000002 | Aculeximycin\_biosynthetic\_gene\_cluster | 39 | 60.9550561798 | 905 | 1.5e-262 |
| AEP40936.1 | polyketide\_synthase\_type\_I | BGC0000021 | Apoptolidin\_biosynthetic\_gene\_cluster | 39 | 59.911717496 | 902 | 1.3e-261 |
| CAJ88177.1 | putative\_type\_I\_polyketide\_synthase | BGC0000151 | Stambomycin\_biosynthetic\_gene\_cluster | 39 | 63.4430176565 | 901 | 1.7e-261 |
| ABJ97439.1 | MerC | BGC0001012 | Meridamycin\_biosynthetic\_gene\_cluster | 38 | 66.1316211878 | 901 | 2.2e-261 |
| AEZ64504.1 | Herc | BGC0001065 | Herboxidiene\_biosynthetic\_gene\_cluster | 38 | 65.2889245586 | 901 | 2.2e-261 |
| ABK32256.1 | AmbB | BGC0000014 | Ambruticin\_biosynthetic\_gene\_cluster | 38 | 60.0321027287 | 898 | 2.4e-260 |
| BAE93729.1 | type\_I\_polyketide\_synthase | BGC0000164 | Tetronomycin\_biosynthetic\_gene\_cluster | 36 | 68.5393258427 | 896 | 5.4e-260 |
| AEH42490.1 | polyketide\_synthase | BGC0000032 | Calcimycin\_biosynthetic\_gene\_cluster | 38 | 62.4398073836 | 893 | 4.5e-259 |
| ABK32288.1 | JerB | BGC0000080 | Jerangolid\_biosynthetic\_gene\_cluster | 38 | 59.8314606742 | 892 | 1e-258 |
| AHB82059.1 | non\_ribosomal\_peptide\_synthetase/polyketide\_synthase | BGC0001019 | Microsclerodermins\_biosynthetic\_gene\_cluster | 38 | 60.9951845907 | 891 | 1.7e-258 |
| AAG13917.1 | megalomicin\_6-deoxyerythronolide\_B\_synthase\_1 | BGC0000092 | Megalomicin\_biosynthetic\_gene\_cluster | 37 | 63.4028892456 | 891 | 2.9e-258 |
| ALA09355.1 | type\_I\_modular\_PKS | BGC0001303 | Lobosamide\_biosynthetic\_gene\_cluster | 37 | 69.5024077047 | 891 | 2.9e-258 |
| ADB12491.1 | EpoD | BGC0000990 | Epothilone\_biosynthetic\_gene\_cluster | 37 | 65.8105939005 | 889 | 8.6e-258 |
| AAF26921.1 | polyketide\_synthase | BGC0000988 | Epothilone\_biosynthetic\_gene\_cluster | 37 | 65.8105939005 | 888 | 1.9e-257 |
| ACB46195.1 | polyketide\_synthase | BGC0000989 | Epothilone\_biosynthetic\_gene\_cluster | 37 | 67.7768860353 | 887 | 4.3e-257 |
| CAE45668.1 | borrelidin\_polyketide\_synthase,\_type\_I | BGC0000031 | Borrelidin\_biosynthetic\_gene\_cluster | 38 | 60.7544141252 | 886 | 5.6e-257 |
| CBD77746.1 | non-ribosomal\_peptide\_synthetase/polyketide\_synthase | BGC0000974 | Crocacin\_biosynthetic\_gene\_cluster | 38 | 61.6773675762 | 886 | 9.5e-257 |
| AAF62883.1 | epoD | BGC0000991 | Epothilone\_biosynthetic\_gene\_cluster | 37 | 65.8105939005 | 885 | 1.2e-256 |
| AHB82072.1 | non\_ribosomal\_peptide\_synthetase/polyketide\_synthase | BGC0001231 | Microsclerodermins\_biosynthetic\_gene\_cluster | 38 | 61.2760834671 | 881 | 2.3e-255 |
| BAC68126.1 | modular\_polyketide\_synthase | BGC0000059 | Filipin\_biosynthetic\_gene\_cluster | 39 | 60.3531300161 | 879 | 8.9e-255 |
| ctg1\_orf27 |  | BGC0000096 | Midecamycin\_biosynthetic\_gene\_cluster | 39 | 60.1123595506 | 876 | 7.5e-254 |
| CAE45672.1 | Borrelidin\_polyketide\_synthase,\_type\_I | BGC0000031 | Borrelidin\_biosynthetic\_gene\_cluster | 38 | 60.3531300161 | 874 | 2.2e-253 |
| AHH99920.1 | PKS\_I | BGC0000002 | Aculeximycin\_biosynthetic\_gene\_cluster | 37 | 61.9582664526 | 872 | 1.1e-252 |
| CAJ88186.1 | putative\_modular\_polyketide\_synthase | BGC0000151 | Stambomycin\_biosynthetic\_gene\_cluster | 39 | 60.9149277689 | 872 | 1.1e-252 |
| AEP40935.1 | polyketide\_synthase\_type\_I | BGC0000021 | Apoptolidin\_biosynthetic\_gene\_cluster | 36 | 68.86035313 | 872 | 1.4e-252 |
| ABC87510.1 | polyketide\_synthase | BGC0001011 | Meridamycin\_biosynthetic\_gene\_cluster | 36 | 66.2921348315 | 869 | 1.2e-251 |
| ctg1\_orf21 |  | BGC0001013 | Meridamycin\_biosynthetic\_gene\_cluster | 36 | 66.2921348315 | 869 | 1.2e-251 |
| ABY21542.1 | AngAV | BGC0000018 | Angolamycin\_biosynthetic\_gene\_cluster | 40 | 57.7447833066 | 868 | 1.6e-251 |
| CAQ18829.1 | polyketide\_synthase | BGC0000954 | Ajudazol\_biosynthetic\_gene\_cluster | 37 | 62.27929374 | 864 | 3.9e-250 |
| CAJ88184.1 | putative\_modular\_polyketide\_synthase | BGC0000151 | Stambomycin\_biosynthetic\_gene\_cluster | 38 | 64.1252006421 | 864 | 3.9e-250 |
| AAC69329.1 | type\_I\_polyketide\_synthase\_PikAI | BGC0000094 | Methymycin\_/\_pikromycin\_biosynthetic\_gene\_cluster | 37 | 64.4060995185 | 858 | 2.1e-248 |
| CAM00062.1 | EryAI\_Erythromycin\_polyketide\_synthase\_modules\_1\_and\_2 | BGC0000055 | Erythromycin\_biosynthetic\_gene\_cluster | 36 | 63.1219903692 | 857 | 4.7e-248 |
| AEZ64505.1 | Herb | BGC0001065 | Herboxidiene\_biosynthetic\_gene\_cluster | 37 | 65.4494382022 | 845 | 1.9e-244 |
| CAQ64687.1 | lasalocid\_modular\_polyketide\_synthase | BGC0000087 | Lasalocid\_biosynthetic\_gene\_cluster | 35 | 68.86035313 | 843 | 5.4e-244 |
| BAG85027.1 | putative\_polyketide\_synthase | BGC0000086 | Lasalocid\_biosynthetic\_gene\_cluster | 35 | 68.86035313 | 843 | 5.4e-244 |
| ABY21538.1 | AngAI | BGC0000018 | Angolamycin\_biosynthetic\_gene\_cluster | 36 | 64.3258426966 | 842 | 1.2e-243 |
| ABJ97438.1 | MerB | BGC0001012 | Meridamycin\_biosynthetic\_gene\_cluster | 35 | 68.86035313 | 840 | 4.6e-243 |
| AGY62755.1 | EbeC | BGC0000051 | Ebelactone\_biosynthetic\_gene\_cluster | 37 | 60.9149277689 | 840 | 6e-243 |
| AEP40940.1 | polyketide\_synthase\_type\_I | BGC0000021 | Apoptolidin\_biosynthetic\_gene\_cluster | 36 | 66.8138041734 | 838 | 1.7e-242 |
| AGI61662.1 | modular\_PKS | BGC0000095\_c5 | Micromonolactam\_biosynthetic\_gene\_cluster | 38 | 61.1556982343 | 838 | 2.3e-242 |
| BAJ16467.1 | polyketide\_synthase | BGC0000058 | FD-891\_biosynthetic\_gene\_cluster | 36 | 65.4895666132 | 837 | 3e-242 |
| ABB88533.1 | polyketide\_synthase\_type\_I | BGC0000049 | E-492\_/\_E-975\_biosynthetic\_gene\_cluster | 36 | 66.2921348315 | 837 | 3.9e-242 |
| AAQ82561.1 | FscA | BGC0000061 | FR-008\_biosynthetic\_gene\_cluster | 36 | 62.3996789727 | 836 | 8.6e-242 |
| BAC76493.1 | lankamycin\_synthase\_LkmAI | BGC0000085 | Lankamycin\_biosynthetic\_gene\_cluster | 36 | 63.9646869984 | 835 | 1.5e-241 |
| CUW01172.1 | Type\_I\_polyketide\_synthase | BGC0001359 | PM100117\_/\_PM100118\_biosynthetic\_gene\_cluster | 35 | 67.4959871589 | 834 | 3.3e-241 |
| AAU93807.2 | polyketide\_synthase\_modules\_1\_and\_2 | BGC0000054 | Erythromycin\_biosynthetic\_gene\_cluster | 36 | 63.4831460674 | 834 | 4.3e-241 |
| ABC87509.1 | polyketide\_synthase | BGC0001011 | Meridamycin\_biosynthetic\_gene\_cluster | 36 | 64.0048154093 | 832 | 1.2e-240 |
| ctg1\_orf20 |  | BGC0001013 | Meridamycin\_biosynthetic\_gene\_cluster | 36 | 64.0048154093 | 832 | 1.2e-240 |
| ABJ97437.1 | MerA | BGC0001012 | Meridamycin\_biosynthetic\_gene\_cluster | 35 | 65.0481540931 | 825 | 2e-238 |
| AAS79459.1 | polyketide\_synthase\_subunit | BGC0000035 | Chalcomycin\_biosynthetic\_gene\_cluster | 36 | 66.9341894061 | 820 | 6.4e-237 |
| ABP55222.1 | beta-ketoacyl\_synthase | BGC0000142 | Salinilactam\_biosynthetic\_gene\_cluster | 37 | 60.3130016051 | 819 | 1.4e-236 |
| AAM81584.2 | putative\_type\_I\_polyketide\_synthase | BGC0000047 | Dihydrochalcomycin\_biosynthetic\_gene\_cluster | 36 | 65.8908507223 | 817 | 4.1e-236 |
| CAQ34918.1 | nonribosomal\_peptide\_synthetase/\_polyketide\_synthase | BGC0000986 | Dkxanthene\_biosynthetic\_gene\_cluster | 36 | 61.1958266453 | 815 | 2.1e-235 |
| ANC94966.1 | AlmHI | BGC0001396 | Aldgamycin\_biosynthetic\_gene\_cluster | 36 | 64.2857142857 | 814 | 2.7e-235 |
| AEZ64503.1 | Herd | BGC0001065 | Herboxidiene\_biosynthetic\_gene\_cluster | 49 | 35.9149277689 | 814 | 2.7e-235 |
| ADU85981.1 | putative\_modular\_polyketide\_synthase | BGC0000165 | Tiacumicin\_B\_biosynthetic\_gene\_cluster | 37 | 59.6709470305 | 814 | 4.6e-235 |
| AFU82614.1 | mixed\_NRPS\_PKS | BGC0000998 | Guadinomine\_biosynthetic\_gene\_cluster | 36 | 61.9181380417 | 814 | 4.6e-235 |
| CAM00065.1 | EryAIII\_Erythromycin\_polyketide\_synthase\_modules\_5\_and\_6 | BGC0000055 | Erythromycin\_biosynthetic\_gene\_cluster | 36 | 64.3258426966 | 812 | 1.7e-234 |
| AAO06917.1 | GdmAII | BGC0000066 | Geldanamycin\_biosynthetic\_gene\_cluster | 35 | 66.5730337079 | 810 | 6.6e-234 |
| ABB86409.1 | GelB | BGC0000067 | Geldanamycin\_biosynthetic\_gene\_cluster | 35 | 66.6131621188 | 807 | 3.3e-233 |
| ABK32257.1 | AmbC | BGC0000014 | Ambruticin\_biosynthetic\_gene\_cluster | 46 | 41.0112359551 | 806 | 7.3e-233 |
| AAX98191.1 | polyketide\_synthase\_type\_I | BGC0000052 | ECO-02301\_biosynthetic\_gene\_cluster | 45 | 41.974317817 | 806 | 9.6e-233 |
| ADU86002.1 | putative\_modular\_polyketide\_synthase | BGC0000165 | Tiacumicin\_B\_biosynthetic\_gene\_cluster | 35 | 66.1316211878 | 805 | 1.2e-232 |
| AAY28226.1 | HbmAII | BGC0000074 | Herbimycin\_biosynthetic\_gene\_cluster | 35 | 66.3322632424 | 803 | 6.2e-232 |
| ABB88519.1 | polyketide\_synthase\_type\_I | BGC0000050 | E-837\_biosynthetic\_gene\_cluster | 35 | 64.6468699839 | 801 | 3.1e-231 |
| AFU82615.1 | polyketide\_synthase | BGC0000998 | Guadinomine\_biosynthetic\_gene\_cluster | 37 | 60.3130016051 | 797 | 4.4e-230 |
| CBA11582.1 | polyketide\_synthase\_type\_I | BGC0001046 | Streptolydigin\_biosynthetic\_gene\_cluster | 44 | 41.3723916533 | 797 | 4.4e-230 |
| ADX66470.1 | ScnS0 | BGC0000108 | Natamycin\_biosynthetic\_gene\_cluster | 35 | 62.3996789727 | 797 | 5.8e-230 |
| AAX98188.1 | polyketide\_synthase\_type\_I | BGC0000052 | ECO-02301\_biosynthetic\_gene\_cluster | 44 | 41.2118780096 | 796 | 9.9e-230 |
| ABX60161.1 | mixed\_NRPS/PKS | BGC0000978 | Cylindrospermopsin\_biosynthetic\_gene\_cluster | 37 | 58.025682183 | 794 | 2.9e-229 |
| ADF88279.1 | mixed\_NRPS/PKS | BGC0000981 | Cylindrospermopsin\_biosynthetic\_gene\_cluster | 37 | 58.025682183 | 794 | 2.9e-229 |
| ABK32289.1 | JerC | BGC0000080 | Jerangolid\_biosynthetic\_gene\_cluster | 46 | 38.8844301766 | 794 | 2.9e-229 |
| ctg1\_orf30 |  | BGC0000096 | Midecamycin\_biosynthetic\_gene\_cluster | 37 | 61.3563402889 | 793 | 6.4e-229 |
| ABY21541.1 | AngAIV | BGC0000018 | Angolamycin\_biosynthetic\_gene\_cluster | 37 | 61.1155698234 | 790 | 5.4e-228 |
| ADH04660.1 | TugD | BGC0001342 | Thuggacin\_biosynthetic\_gene\_cluster | 47 | 36.316211878 | 789 | 9.3e-228 |
| ADX66461.1 | ScnS2 | BGC0000108 | Natamycin\_biosynthetic\_gene\_cluster | 35 | 66.3322632424 | 789 | 9.3e-228 |
| ABK32259.1 | AmbE | BGC0000014 | Ambruticin\_biosynthetic\_gene\_cluster | 44 | 40.9309791332 | 789 | 1.2e-227 |
| WP\_063764077.1 | hypothetical\_protein | BGC0001348 | JBIR-100\_biosynthetic\_gene\_cluster | 45 | 39.7271268058 | 788 | 2.1e-227 |
| BAB69193.1 |  | BGC0000117 | Oligomycin\_biosynthetic\_gene\_cluster | 36 | 60.7142857143 | 788 | 2.1e-227 |
| CAL58687.1 | polyketide\_synthase | BGC0000149 | Spirangien\_biosynthetic\_gene\_cluster | 47 | 38.683788122 | 788 | 2.7e-227 |
| CAQ52624.1 | type\_I\_polyketide\_synthase,\_modules\_7-8 | BGC0001066 | Kendomycin\_biosynthetic\_gene\_cluster | 43 | 42.6163723917 | 787 | 6e-227 |
| AHD05614.1 | putative\_non-ribosomal\_peptide\_ligase/\_polyketide\_synthase\_hybrid | BGC0001033 | Paenilamicin\_biosynthetic\_gene\_cluster | 34 | 61.4365971108 | 785 | 2.3e-226 |
| ABB88522.1 | polyketide\_synthase\_type\_I | BGC0000050 | E-837\_biosynthetic\_gene\_cluster | 36 | 64.0449438202 | 784 | 3e-226 |
| AAX98184.1 | polyketide\_synthase\_type\_I | BGC0000052 | ECO-02301\_biosynthetic\_gene\_cluster | 45 | 40.6099518459 | 783 | 6.6e-226 |
| AFI57005.1 | QmnA1 | BGC0000133 | Quartromicin\_biosynthetic\_gene\_cluster | 42 | 46.4686998395 | 783 | 8.7e-226 |
| ALA09371.1 | type\_I\_modular\_PKS | BGC0001303 | Lobosamide\_biosynthetic\_gene\_cluster | 45 | 39.4060995185 | 783 | 8.7e-226 |
| CAL58683.1 | polyketide\_synthase | BGC0000149 | Spirangien\_biosynthetic\_gene\_cluster | 48 | 36.0353130016 | 782 | 1.1e-225 |
| ADH04659.1 | TugC | BGC0001342 | Thuggacin\_biosynthetic\_gene\_cluster | 47 | 36.9181380417 | 782 | 1.1e-225 |
| AAY28227.1 | HbmAIII | BGC0000074 | Herbimycin\_biosynthetic\_gene\_cluster | 47 | 36.7576243981 | 782 | 1.1e-225 |
| AJO72736.1 | Type\_I\_modular\_polyketide\_synthase | BGC0001381 | Brasilinolide\_biosynthetic\_gene\_cluster | 35 | 64.7271268058 | 782 | 1.5e-225 |
| AAG13919.1 | megalomicin\_6-deoxyerythronolide\_B\_synthase\_3 | BGC0000092 | Megalomicin\_biosynthetic\_gene\_cluster | 35 | 60.5939004815 | 782 | 1.5e-225 |
| CAC20930.1 | PimS0\_protein | BGC0000125 | Pimaricin\_biosynthetic\_gene\_cluster | 35 | 60.6340288925 | 782 | 1.5e-225 |
| AAX98186.1 | polyketide\_synthase\_type\_I | BGC0000052 | ECO-02301\_biosynthetic\_gene\_cluster | 45 | 39.606741573 | 782 | 1.5e-225 |
| ABP55210.1 | beta-ketoacyl\_synthase | BGC0000142 | Salinilactam\_biosynthetic\_gene\_cluster | 44 | 40.088282504 | 781 | 2.5e-225 |
| ABV99085.1 | thioester\_reductase\_domain | BGC0001007 | Lymphostin\_biosynthetic\_gene\_cluster | 35 | 63.8844301766 | 781 | 3.3e-225 |
| ADB12492.1 | EpoE | BGC0000990 | Epothilone\_biosynthetic\_gene\_cluster | 35 | 62.0786516854 | 780 | 4.3e-225 |
| AAX98189.1 | polyketide\_synthase\_type\_I | BGC0000052 | ECO-02301\_biosynthetic\_gene\_cluster | 45 | 39.2455858748 | 780 | 5.6e-225 |
| CUW01177.1 | Type\_I\_polyketide\_synthase | BGC0001359\_c2 | PM100117\_/\_PM100118\_biosynthetic\_gene\_cluster | 45 | 39.4060995185 | 780 | 7.3e-225 |
| CUW01174.1 | Type\_I\_polyketide\_synthase | BGC0001359 | PM100117\_/\_PM100118\_biosynthetic\_gene\_cluster | 44 | 41.9341894061 | 780 | 7.3e-225 |
| AHA38203.1 | GphJ | BGC0000069 | Gephyronic\_acid\_biosynthetic\_gene\_cluster | 36 | 61.9582664526 | 780 | 7.3e-225 |
| CAL58686.1 | polyketide\_synthase | BGC0000149 | Spirangien\_biosynthetic\_gene\_cluster | 46 | 38.3627608347 | 779 | 9.6e-225 |
| ABP55221.1 | acyl\_transferase\_domain\_protein | BGC0000142 | Salinilactam\_biosynthetic\_gene\_cluster | 37 | 61.797752809 | 779 | 9.6e-225 |
| AAC46027.1 | polyketide\_synthase\_module\_6 | BGC0000113 | Niddamycin\_biosynthetic\_gene\_cluster | 35 | 61.8780096308 | 779 | 9.6e-225 |
| AAF26922.1 | polyketide\_synthase | BGC0000988 | Epothilone\_biosynthetic\_gene\_cluster | 35 | 62.0385232745 | 779 | 1.3e-224 |
| AGC45620.1 | polyketide\_synthase | BGC0001394 | Phenalamide\_biosynthetic\_gene\_cluster | 34 | 66.0914927769 | 779 | 1.6e-224 |
| AAF62884.1 | EpoE | BGC0000991 | Epothilone\_biosynthetic\_gene\_cluster | 35 | 62.0786516854 | 778 | 2.8e-224 |
| AAX98192.1 | polyketide\_synthase\_type\_I | BGC0000052 | ECO-02301\_biosynthetic\_gene\_cluster | 44 | 41.2118780096 | 777 | 6.2e-224 |
| AJO72743.1 | Type\_I\_modular\_polyketide\_synthase | BGC0001381 | Brasilinolide\_biosynthetic\_gene\_cluster | 36 | 64.4462279294 | 776 | 1.1e-223 |
| ABK32291.1 | JerE | BGC0000080 | Jerangolid\_biosynthetic\_gene\_cluster | 46 | 37.8009630819 | 775 | 1.4e-223 |
| CAL58685.1 | polyketide\_synthase | BGC0000149 | Spirangien\_biosynthetic\_gene\_cluster | 43 | 40.5296950241 | 775 | 1.8e-223 |
| ABF87031.1 | non-ribosomal\_peptide\_synthetase/polyketide\_synthase | BGC0000393 | Myxoprincomide\_biosynthetic\_gene\_cluster | 35 | 61.5569823435 | 774 | 5.3e-223 |
| ADZ24995.1 | non-ribosomal\_peptide\_synthase/polyketide\_synthase | BGC0000380 | Leupyrrin\_biosynthetic\_gene\_cluster | 34 | 61.9582664526 | 774 | 5.3e-223 |
| ABK32290.1 | JerD | BGC0000080 | Jerangolid\_biosynthetic\_gene\_cluster | 47 | 36.797752809 | 774 | 5.3e-223 |
| ACB46196.1 | polyketide\_synthase | BGC0000989 | Epothilone\_biosynthetic\_gene\_cluster | 35 | 62.0786516854 | 772 | 1.2e-222 |
| AAK57187.1 | MxaC | BGC0001022 | Myxalamid\_biosynthetic\_gene\_cluster | 32 | 70.304975923 | 772 | 1.5e-222 |
| ABK32258.1 | AmbD | BGC0000014 | Ambruticin\_biosynthetic\_gene\_cluster | 47 | 37.0786516854 | 772 | 2e-222 |
| AAZ94387.1 | modular\_polyketide\_synthase | BGC0000040 | Concanamycin\_A\_biosynthetic\_gene\_cluster | 44 | 39.6468699839 | 771 | 2.6e-222 |
| AAO06918.1 | GdmAIII | BGC0000066 | Geldanamycin\_biosynthetic\_gene\_cluster | 47 | 36.7576243981 | 771 | 2.6e-222 |
| AAQ82568.1 | FscD | BGC0000061 | FR-008\_biosynthetic\_gene\_cluster | 44 | 39.5666131621 | 771 | 3.4e-222 |
| AAU93805.2 | polyketide\_synthase\_modules\_5\_and\_6 | BGC0000054 | Erythromycin\_biosynthetic\_gene\_cluster | 35 | 63.7239165329 | 771 | 3.4e-222 |
| CAC20921.1 | PimS2\_protein | BGC0000125 | Pimaricin\_biosynthetic\_gene\_cluster | 45 | 38.4831460674 | 771 | 3.4e-222 |
| ADH04641.1 | TgaC | BGC0001051 | Thuggacin\_biosynthetic\_gene\_cluster | 45 | 36.9983948636 | 770 | 7.6e-222 |
| ABB86410.1 | GelC | BGC0000067 | Geldanamycin\_biosynthetic\_gene\_cluster | 47 | 36.7576243981 | 770 | 7.6e-222 |
| AAR16521.1 | RimA | BGC0000138 | Rimocidin\_biosynthetic\_gene\_cluster | 34 | 62.0786516854 | 770 | 7.6e-222 |
| AHH99921.1 | PKS\_I | BGC0000002 | Aculeximycin\_biosynthetic\_gene\_cluster | 45 | 39.8876404494 | 769 | 9.9e-222 |
| AAC69331.1 | type\_I\_polyketide\_synthase\_PikAIII | BGC0000094 | Methymycin\_/\_pikromycin\_biosynthetic\_gene\_cluster | 36 | 60.6340288925 | 769 | 1.3e-221 |
| CAC20931.1 | PimS1\_protein | BGC0000125 | Pimaricin\_biosynthetic\_gene\_cluster | 43 | 41.5730337079 | 768 | 2.9e-221 |
| ABP55493.1 | thioester\_reductase\_domain | BGC0001006 | Lymphostin\_biosynthetic\_gene\_cluster | 35 | 62.9213483146 | 767 | 3.8e-221 |
| AHN85651.1 | Phn2 | BGC0000122 | Phenylnannolone\_A\_biosynthetic\_gene\_cluster | 43 | 40.7303370787 | 767 | 6.4e-221 |
| ADX66472.1 | ScnS1 | BGC0000108 | Natamycin\_biosynthetic\_gene\_cluster | 43 | 41.6934189406 | 767 | 6.4e-221 |
| ADH04640.1 | TgaB | BGC0001051 | Thuggacin\_biosynthetic\_gene\_cluster | 43 | 42.0947030498 | 765 | 2.4e-220 |
| AAR87760.2 | ZmaK | BGC0001059 | Zwittermycin\_A\_biosynthetic\_gene\_cluster | 33 | 60.9149277689 | 764 | 3.2e-220 |
| AAA79984.2 | soraphen\_polyketide\_synthase\_B | BGC0000147 | Soraphen\_biosynthetic\_gene\_cluster | 46 | 35.834670947 | 764 | 3.2e-220 |
| ALA09356.1 | type\_I\_modular\_PKS | BGC0001303 | Lobosamide\_biosynthetic\_gene\_cluster | 36 | 61.9582664526 | 763 | 7.1e-220 |
| ctg1\_orf7 |  | BGC0000053 | Elaiophylin\_biosynthetic\_gene\_cluster | 44 | 39.9277688604 | 763 | 9.3e-220 |
| ANC94965.1 | AlmHII | BGC0001396 | Aldgamycin\_biosynthetic\_gene\_cluster | 46 | 37.72070626 | 762 | 1.2e-219 |
| BAJ16469.1 | polyketide\_synthase | BGC0000058 | FD-891\_biosynthetic\_gene\_cluster | 45 | 36.3964686998 | 762 | 1.6e-219 |
| AAM81585.1 | putative\_type\_I\_polyketide\_synthase | BGC0000047 | Dihydrochalcomycin\_biosynthetic\_gene\_cluster | 46 | 37.72070626 | 762 | 2.1e-219 |
| AAK19883.1 | soraphen\_polyketide\_synthase\_A | BGC0000147 | Soraphen\_biosynthetic\_gene\_cluster | 43 | 39.5666131621 | 762 | 2.1e-219 |
| AAZ94388.1 | nodular\_polyketide\_synthase | BGC0000040 | Concanamycin\_A\_biosynthetic\_gene\_cluster | 44 | 39.2857142857 | 761 | 3.5e-219 |
| ABV83221.1 | CppI | BGC0000116 | Nystatin-like\_Pseudonocardia\_polyene\_biosynthetic\_gene\_cluster | 42 | 42.455858748 | 761 | 3.5e-219 |
| BAK64650.1 | polyketide\_synthase | BGC0000135 | Reveromycin\_biosynthetic\_gene\_cluster | 33 | 69.0609951846 | 760 | 4.6e-219 |
| BAT51066.1 | type\_I\_polyketide\_synthase | BGC0001296 | Streptazone\_E\_biosynthetic\_gene\_cluster | 42 | 41.051364366 | 760 | 4.6e-219 |
| BAF02926.1 | type\_I\_polyketide\_synthase | BGC0000073 | Halstoctacosanolide\_biosynthetic\_gene\_cluster | 42 | 41.6532905297 | 760 | 4.6e-219 |
| BAT51065.1 | type\_I\_polyketide\_synthase | BGC0001296 | Streptazone\_E\_biosynthetic\_gene\_cluster | 44 | 39.2857142857 | 759 | 1e-218 |
| AAZ77694.1 | ChlA2 | BGC0000036 | Chlorothricin\_biosynthetic\_gene\_cluster | 43 | 39.5666131621 | 759 | 1e-218 |
| BAF02921.1 | type\_I\_polyketide\_synthase | BGC0000073 | Halstoctacosanolide\_biosynthetic\_gene\_cluster | 44 | 41.1717495987 | 759 | 1e-218 |
| BAO66519.1 | type\_I\_polyketide\_synthase | BGC0000042 | Cremimycin\_biosynthetic\_gene\_cluster | 43 | 42.0144462279 | 759 | 1.3e-218 |
| AAZ77696.1 | ChlA3 | BGC0000036 | Chlorothricin\_biosynthetic\_gene\_cluster | 43 | 40.7303370787 | 759 | 1.3e-218 |
| AHE80996.1 | PieA6 | BGC0001169 | Piericidin\_A1\_biosynthetic\_gene\_cluster | 44 | 39.6869983949 | 759 | 1.3e-218 |
| ABB52545.1 | putative\_type\_I\_polyketide\_synthase | BGC0000047 | Dihydrochalcomycin\_biosynthetic\_gene\_cluster | 45 | 36.5971107544 | 758 | 1.7e-218 |
| AAS79463.1 | polyketide\_synthase\_subunit | BGC0000035 | Chalcomycin\_biosynthetic\_gene\_cluster | 45 | 36.6773675762 | 758 | 2.3e-218 |
| AAZ94386.1 | modular\_polyketide\_synthase | BGC0000040 | Concanamycin\_A\_biosynthetic\_gene\_cluster | 43 | 40.7704654896 | 758 | 3e-218 |
| AAG23263.1 | polyketide\_synthase\_extender\_modules\_5-7 | BGC0000148 | Spinosad\_biosynthetic\_gene\_cluster | 43 | 40.3691813804 | 758 | 3e-218 |
| ADC79639.1 | TamAIII | BGC0001052 | Tirandamycin\_biosynthetic\_gene\_cluster | 43 | 39.2054574639 | 758 | 3e-218 |
| BAR73017.1 | putative\_PKS\_(KS-AT-KR-ACP-KS-AT-DH-KR-ACP) | BGC0001194 | Hitachimycin\_biosynthetic\_gene\_cluster | 46 | 35.9550561798 | 757 | 3.9e-218 |
| AAK73502.1 | AmphJ | BGC0000015 | Amphotericin\_biosynthetic\_gene\_cluster | 43 | 39.0850722311 | 757 | 5.1e-218 |
| BAH02270.1 | polyketide\_synthase | BGC0000126 | Pladienolide\_biosynthetic\_gene\_cluster | 44 | 39.165329053 | 757 | 5.1e-218 |
| AAX98185.1 | polyketide\_synthase\_type\_I | BGC0000052 | ECO-02301\_biosynthetic\_gene\_cluster | 44 | 39.1252006421 | 757 | 5.1e-218 |
| WP\_043476519.1 | polyketide\_synthase | BGC0001232 | Leucanicidin\_biosynthetic\_gene\_cluster | 44 | 40.4895666132 | 757 | 6.6e-218 |
| AFV30247.1 | polyketide\_synthase | BGC0000075 | Hygrocin\_biosynthetic\_gene\_cluster | 44 | 39.7271268058 | 756 | 1.1e-217 |
| AAC46028.1 | polyketide\_synthase\_module\_7 | BGC0000113 | Niddamycin\_biosynthetic\_gene\_cluster | 45 | 37.8410914928 | 756 | 1.1e-217 |
| ABB88521.1 | polyketide\_synthase\_type\_I | BGC0000050 | E-837\_biosynthetic\_gene\_cluster | 46 | 35.9951845907 | 755 | 1.5e-217 |
| BAB69194.1 | modular\_polyketide\_synthase | BGC0000117 | Oligomycin\_biosynthetic\_gene\_cluster | 44 | 39.165329053 | 755 | 1.9e-217 |
| ANC94962.1 | AlmHV | BGC0001396 | Aldgamycin\_biosynthetic\_gene\_cluster | 45 | 36.5971107544 | 755 | 1.9e-217 |
| BAJ16468.1 | polyketide\_synthase | BGC0000058 | FD-891\_biosynthetic\_gene\_cluster | 43 | 41.051364366 | 755 | 2.5e-217 |
| AAQ82564.1 | FscC | BGC0000034 | Candicidin\_biosynthetic\_gene\_cluster | 43 | 41.051364366 | 754 | 3.3e-217 |
| ctg1\_orf254 |  | BGC0001200 | Lorneic\_acid\_A\_biosynthetic\_gene\_cluster | 43 | 39.165329053 | 754 | 4.3e-217 |
| AAS79460.1 | polyketide\_synthase\_subunit | BGC0000035 | Chalcomycin\_biosynthetic\_gene\_cluster | 45 | 37.9213483146 | 754 | 4.3e-217 |
| AAC69330.1 | type\_I\_polyketide\_synthase\_PikAII | BGC0000094 | Methymycin\_/\_pikromycin\_biosynthetic\_gene\_cluster | 35 | 62.4398073836 | 754 | 4.3e-217 |
| CAD55506.1 | putative\_type\_I\_polyketide\_synthase | BGC0000038 | Coelimycin\_biosynthetic\_gene\_cluster | 43 | 39.2054574639 | 753 | 7.3e-217 |
| AAF71776.1 | NysC | BGC0000115 | Nystatin\_biosynthetic\_gene\_cluster | 44 | 39.3258426966 | 753 | 9.6e-217 |
| ACO94488.1 | polyketide\_synthase\_type\_I | BGC0000097 | ML-449\_biosynthetic\_gene\_cluster | 42 | 41.8539325843 | 752 | 1.3e-216 |
| ACO94460.1 | polyketide\_synthase\_type\_I | BGC0000029 | BE-14106\_biosynthetic\_gene\_cluster | 42 | 41.7736757624 | 752 | 1.6e-216 |
| BAO66539.1 | type\_I\_polyketide\_synthase | BGC0000042 | Cremimycin\_biosynthetic\_gene\_cluster | 45 | 39.2054574639 | 752 | 1.6e-216 |
| BAR73007.1 | putative\_PKS\_(ACP-KS-AT-DH-KR-ACP-KS-AT-DH-ER-KR-ACP) | BGC0001194 | Hitachimycin\_biosynthetic\_gene\_cluster | 43 | 42.6163723917 | 752 | 2.1e-216 |
| CCP20051.1 | divM\_protein | BGC0001119 | Divergolide\_biosynthetic\_gene\_cluster | 45 | 39.3659711075 | 751 | 2.8e-216 |
| AAZ77697.1 | ChlA4 | BGC0000036 | Chlorothricin\_biosynthetic\_gene\_cluster | 46 | 36.4767255217 | 750 | 4.8e-216 |
| AGY30676.1 | Ann4 | BGC0001298 | 4-Z-annimycin\_biosynthetic\_gene\_cluster | 44 | 39.9277688604 | 750 | 8.1e-216 |
| AAK73514.1 | AmphC | BGC0000015 | Amphotericin\_biosynthetic\_gene\_cluster | 44 | 40.7303370787 | 749 | 1.1e-215 |
| ctg1\_orf29 |  | BGC0000096 | Midecamycin\_biosynthetic\_gene\_cluster | 36 | 61.0754414125 | 749 | 1.1e-215 |
| BAH02268.1 | polyketide\_synthase | BGC0000126 | Pladienolide\_biosynthetic\_gene\_cluster | 44 | 39.606741573 | 749 | 1.4e-215 |
| BAF02924.1 | type\_I\_polyketide\_synthase | BGC0000073 | Halstoctacosanolide\_biosynthetic\_gene\_cluster | 43 | 42.3756019262 | 749 | 1.4e-215 |
| AAB66504.1 | tylactone\_synthase\_starter\_module\_and\_modules\_1\_&\_2 | BGC0000166 | Tylactone\_biosynthetic\_gene\_cluster | 34 | 65.1284109149 | 748 | 1.8e-215 |
| ABB05105.1 | LipPks4 | BGC0001003 | Lipomycin\_biosynthetic\_gene\_cluster | 43 | 39.2455858748 | 748 | 1.8e-215 |
| ctg1\_orf31 |  | BGC0000096 | Midecamycin\_biosynthetic\_gene\_cluster | 45 | 36.2359550562 | 748 | 2.4e-215 |
| BAO66529.1 | type\_I\_polyketide\_synthase | BGC0000042 | Cremimycin\_biosynthetic\_gene\_cluster | 44 | 39.3659711075 | 748 | 3.1e-215 |
| ACA99172.1 | Polyketide\_synthase | BGC0001160 | Nonadecene\_biosynthetic\_gene\_cluster | 31 | 74.518459069 | 748 | 3.1e-215 |
| ACB46485.1 | polyketide\_synthase | BGC0000082 | Kijanimicin\_biosynthetic\_gene\_cluster | 46 | 36.5569823435 | 747 | 4e-215 |
| ACB37743.1 | putative\_type\_I\_polyketide\_synthase | BGC0000162 | Tetrocarcin\_A\_biosynthetic\_gene\_cluster | 45 | 38.0417335474 | 747 | 4e-215 |
| ACB37741.1 | putative\_type\_I\_polyketide\_synthase | BGC0000162 | Tetrocarcin\_A\_biosynthetic\_gene\_cluster | 42 | 42.3756019262 | 747 | 4e-215 |
| AEZ53952.1 | polyketide\_synthase | BGC0000144 | Salinomycin\_biosynthetic\_gene\_cluster | 43 | 39.4462279294 | 747 | 4e-215 |
| WP\_048832936.1 | polyketide\_synthase | BGC0001348 | JBIR-100\_biosynthetic\_gene\_cluster | 43 | 40.2889245586 | 747 | 5.3e-215 |
| BAF02923.1 | type\_I\_polyketide\_synthase | BGC0000073 | Halstoctacosanolide\_biosynthetic\_gene\_cluster | 43 | 40.9711075441 | 747 | 6.9e-215 |
| AAZ77699.1 | ChlA6 | BGC0000036 | Chlorothricin\_biosynthetic\_gene\_cluster | 44 | 36.9582664526 | 745 | 1.5e-214 |
| BAK64637.1 | polyketide\_synthase | BGC0000135 | Reveromycin\_biosynthetic\_gene\_cluster | 35 | 62.4398073836 | 745 | 2e-214 |
| ABB05104.1 | LipPks3 | BGC0001003 | Lipomycin\_biosynthetic\_gene\_cluster | 42 | 40.0481540931 | 745 | 2e-214 |
| CUW01173.1 | Type\_I\_polyketide\_synthase | BGC0001359 | PM100117\_/\_PM100118\_biosynthetic\_gene\_cluster | 46 | 36.5971107544 | 745 | 2.6e-214 |
| BAH02269.1 | polyketide\_synthase | BGC0000126 | Pladienolide\_biosynthetic\_gene\_cluster | 46 | 35.9550561798 | 745 | 2.6e-214 |
| AAG23262.1 | polyketide\_synthase\_extender\_modules\_8-10 | BGC0000148 | Spinosad\_biosynthetic\_gene\_cluster | 43 | 40.1685393258 | 744 | 3.4e-214 |
| BAC68129.1 | modular\_polyketide\_synthase | BGC0000059 | Filipin\_biosynthetic\_gene\_cluster | 43 | 41.3322632424 | 744 | 3.4e-214 |
| AAZ77698.1 | ChlA5 | BGC0000036 | Chlorothricin\_biosynthetic\_gene\_cluster | 45 | 35.9550561798 | 744 | 4.5e-214 |
| AAF71767.1 | NysJ | BGC0000115 | Nystatin\_biosynthetic\_gene\_cluster | 44 | 40.1685393258 | 743 | 5.8e-214 |
| ABC87512.1 | polyketide\_synthase | BGC0001011 | Meridamycin\_biosynthetic\_gene\_cluster | 43 | 40.569823435 | 743 | 7.6e-214 |
| ctg1\_orf23 |  | BGC0001013 | Meridamycin\_biosynthetic\_gene\_cluster | 43 | 40.569823435 | 743 | 7.6e-214 |
| AKD43753.1 | HerB | BGC0001349 | Heronamide\_biosynthetic\_gene\_cluster | 42 | 42.2552166934 | 743 | 9.9e-214 |
| ctg1\_orf256 |  | BGC0001200 | Lorneic\_acid\_A\_biosynthetic\_gene\_cluster | 42 | 40.569823435 | 743 | 9.9e-214 |
| BAF02925.1 | type\_I\_polyketide\_synthase | BGC0000073 | Halstoctacosanolide\_biosynthetic\_gene\_cluster | 44 | 39.3258426966 | 742 | 1.7e-213 |
| AAQ82567.1 | FscE | BGC0000061 | FR-008\_biosynthetic\_gene\_cluster | 41 | 41.532905297 | 741 | 2.9e-213 |
| ABV83230.1 | CppC | BGC0000116 | Nystatin-like\_Pseudonocardia\_polyene\_biosynthetic\_gene\_cluster | 42 | 44.1011235955 | 740 | 6.4e-213 |
| CAO85897.1 | modular\_polyketide\_synthase\_NorB | BGC0000110 | Neoaureothin\_biosynthetic\_gene\_cluster | 44 | 35.9149277689 | 740 | 8.4e-213 |
| ctg1\_orf10 |  | BGC0000053 | Elaiophylin\_biosynthetic\_gene\_cluster | 44 | 38.8443017657 | 740 | 8.4e-213 |
| AAZ77693.1 | ChlA1 | BGC0000036 | Chlorothricin\_biosynthetic\_gene\_cluster | 43 | 40.6902086677 | 739 | 1.4e-212 |
| CAJ88175.1 | putative\_polyketide\_synthase\_B | BGC0000151 | Stambomycin\_biosynthetic\_gene\_cluster | 46 | 36.1556982343 | 738 | 1.9e-212 |
| CAQ64686.1 | lasalocid\_modular\_polyketide\_synthase | BGC0000087 | Lasalocid\_biosynthetic\_gene\_cluster | 42 | 42.0947030498 | 738 | 1.9e-212 |
| BAG85026.1 | putative\_polyketide\_synthase | BGC0000086 | Lasalocid\_biosynthetic\_gene\_cluster | 42 | 42.0947030498 | 738 | 1.9e-212 |
| CAM00064.1 | EryAII\_Erythromycin\_polyketide\_synthase\_modules\_3\_and\_4 | BGC0000055 | Erythromycin\_biosynthetic\_gene\_cluster | 34 | 61.8378812199 | 738 | 1.9e-212 |
| CAQ52626.1 | type\_I\_polyketide\_synthase,\_loading\_module\_and\_modules\_1-3 | BGC0001066 | Kendomycin\_biosynthetic\_gene\_cluster | 42 | 40.9309791332 | 738 | 2.4e-212 |
| CAE02605.1 | polyketide\_synthase\_type\_I | BGC0000024 | Aureothin\_biosynthetic\_gene\_cluster | 45 | 35.8747993579 | 738 | 3.2e-212 |
| AAG23266.1 | polyketide\_synthase\_extender\_modules\_3-4 | BGC0000148 | Spinosad\_biosynthetic\_gene\_cluster | 42 | 39.6869983949 | 736 | 9.3e-212 |
| BAF02922.1 | type\_I\_polyketide\_synthase | BGC0000073 | Halstoctacosanolide\_biosynthetic\_gene\_cluster | 43 | 41.8539325843 | 736 | 1.2e-211 |
| AGI99496.1 | Type\_I\_polyketide\_synthase | BGC0001004 | Lobophorin\_biosynthetic\_gene\_cluster | 44 | 39.4060995185 | 735 | 1.6e-211 |
| ACB46487.1 | polyketide\_synthase | BGC0000082 | Kijanimicin\_biosynthetic\_gene\_cluster | 43 | 40.1284109149 | 735 | 1.6e-211 |
| BAO66542.1 | type\_I\_polyketide\_synthase | BGC0000042 | Cremimycin\_biosynthetic\_gene\_cluster | 43 | 40.0481540931 | 735 | 2.7e-211 |
| AAG23264.1 | polyketide\_synthase\_loading\_and\_extender\_module\_1 | BGC0000148 | Spinosad\_biosynthetic\_gene\_cluster | 40 | 43.41894061 | 735 | 2.7e-211 |
| AAB66507.1 | tylactone\_synthase\_module\_6 | BGC0000166 | Tylactone\_biosynthetic\_gene\_cluster | 35 | 60.9550561798 | 734 | 3.5e-211 |
| AKD43768.1 | HerA1 | BGC0001349 | Heronamide\_biosynthetic\_gene\_cluster | 43 | 39.8475120385 | 734 | 4.6e-211 |
| BAB69199.1 | modular\_polyketide\_synthase | BGC0000117 | Oligomycin\_biosynthetic\_gene\_cluster | 44 | 37.6805778491 | 734 | 4.6e-211 |
| AHH99926.1 | PKS\_I | BGC0000002 | Aculeximycin\_biosynthetic\_gene\_cluster | 44 | 38.9646869984 | 734 | 4.6e-211 |
| AEZ53945.1 | polyketide\_synthase | BGC0000144 | Salinomycin\_biosynthetic\_gene\_cluster | 42 | 44.4622792937 | 732 | 1.3e-210 |
| AGI99494.1 | Type\_I\_polyketide\_synthase | BGC0001004 | Lobophorin\_biosynthetic\_gene\_cluster | 44 | 37.4799357945 | 732 | 1.8e-210 |
| AEZ53946.1 | polyketide\_synthase | BGC0000144 | Salinomycin\_biosynthetic\_gene\_cluster | 42 | 40.6902086677 | 731 | 3.9e-210 |
| AAQ84145.1 | Plm5 | BGC0000123 | Phoslactomycin\_B\_biosynthetic\_gene\_cluster | 45 | 38.2423756019 | 730 | 6.7e-210 |
| BAE93722.1 | type\_I\_polyketide\_synthase | BGC0000164 | Tetronomycin\_biosynthetic\_gene\_cluster | 42 | 39.4863563403 | 730 | 6.7e-210 |
| ctg1\_orf253 |  | BGC0001200 | Lorneic\_acid\_A\_biosynthetic\_gene\_cluster | 42 | 39.2455858748 | 730 | 6.7e-210 |
| AEZ54378.1 | PieA5 | BGC0000124 | Piericidin\_A1\_biosynthetic\_gene\_cluster | 42 | 40.4895666132 | 730 | 8.7e-210 |
| CAJ88185.2 | putative\_modular\_polyketide\_synthase | BGC0000151 | Stambomycin\_biosynthetic\_gene\_cluster | 42 | 41.0914927769 | 729 | 1.1e-209 |
| AGC09487.1 | LobS5 | BGC0001183 | Lobophorin\_biosynthetic\_gene\_cluster | 44 | 37.4398073836 | 729 | 1.5e-209 |
| BAC57031.1 | protomycinolide\_IV\_synthase\_4 | BGC0000102 | Mycinamicin\_biosynthetic\_gene\_cluster | 36 | 61.0754414125 | 728 | 1.9e-209 |
| BAQ21948.1 | putative\_type\_I\_polyketide\_synthase | BGC0001204 | Versipelostatin\_biosynthetic\_gene\_cluster | 45 | 36.2760834671 | 728 | 1.9e-209 |
| AJO72734.1 | Type\_I\_modular\_polyketide\_synthase | BGC0001381 | Brasilinolide\_biosynthetic\_gene\_cluster | 34 | 65.3691813804 | 728 | 2.5e-209 |
| ACR50774.1 | polyketide\_synthase | BGC0000163 | Tetronasin\_biosynthetic\_gene\_cluster | 41 | 41.7335473515 | 728 | 2.5e-209 |
| ABC84471.1 | NigAVII | BGC0000114 | Nigericin\_biosynthetic\_gene\_cluster | 41 | 42.455858748 | 728 | 2.5e-209 |
| AAF71774.1 | NysA | BGC0000115 | Nystatin\_biosynthetic\_gene\_cluster | 45 | 36.4767255217 | 727 | 7.4e-209 |
| CAC22145.1 | putative\_type\_I\_polyketide\_synthase | BGC0000038 | Coelimycin\_biosynthetic\_gene\_cluster | 43 | 39.6869983949 | 726 | 9.6e-209 |
| AHH25595.1 | PKS | BGC0000957 | Ansatrienin\_(mycotrienin)\_biosynthetic\_gene\_cluster | 41 | 42.0545746388 | 726 | 1.3e-208 |
| BAO66528.1 | type\_I\_polyketide\_synthase | BGC0000042 | Cremimycin\_biosynthetic\_gene\_cluster | 45 | 36.0754414125 | 726 | 1.3e-208 |
| BAQ21939.1 | putative\_type\_I\_polyketide\_synthase | BGC0001204 | Versipelostatin\_biosynthetic\_gene\_cluster | 41 | 43.9004815409 | 726 | 1.3e-208 |
| ACY06289.1 | type\_I\_polyketide\_synthase | BGC0001042 | Sanglifehrin\_A\_biosynthetic\_gene\_cluster | 44 | 39.606741573 | 725 | 2.1e-208 |
| AFI57007.1 | QmnA3 | BGC0000133 | Quartromicin\_biosynthetic\_gene\_cluster | 42 | 39.5666131621 | 725 | 2.8e-208 |
| BAC57028.1 | protomycinolide\_IV\_synthase\_1 | BGC0000102 | Mycinamicin\_biosynthetic\_gene\_cluster | 41 | 40.8105939005 | 724 | 3.7e-208 |
| ABB05103.1 | LipPks2 | BGC0001003 | Lipomycin\_biosynthetic\_gene\_cluster | 42 | 40.2889245586 | 724 | 3.7e-208 |
| CAI94682.1 | putative\_polyketide\_synthase | BGC0000141 | Rubradirin\_biosynthetic\_gene\_cluster | 41 | 41.9341894061 | 724 | 3.7e-208 |
| BAC68125.1 | modular\_polyketide\_synthase | BGC0000059 | Filipin\_biosynthetic\_gene\_cluster | 46 | 35.9550561798 | 724 | 4.8e-208 |
| AAP85335.1 | type\_I\_PKS | BGC0000233 | Hedamycin\_biosynthetic\_gene\_cluster | 43 | 37.8009630819 | 724 | 4.8e-208 |
| ACR50773.1 | polyketide\_synthase | BGC0000163 | Tetronasin\_biosynthetic\_gene\_cluster | 40 | 44.1011235955 | 724 | 4.8e-208 |
| BAQ25511.1 | type\_I\_polyketide\_synthase | BGC0001288 | Maklamicin\_biosynthetic\_gene\_cluster | 43 | 39.3659711075 | 723 | 6.2e-208 |
| AAB66505.1 | tylactone\_synthase\_module\_3 | BGC0000166 | Tylactone\_biosynthetic\_gene\_cluster | 44 | 37.1990369181 | 723 | 6.2e-208 |
| ABV97151.1 | AMP-dependent\_synthetase\_and\_ligase | BGC0000137 | Rifamycin\_biosynthetic\_gene\_cluster | 40 | 44.1011235955 | 723 | 1.1e-207 |
| ADM46356.1 | polyketide\_synthase | BGC0000106 | Naphthomycin\_biosynthetic\_gene\_cluster | 42 | 40.4494382022 | 723 | 1.1e-207 |
| AHH99923.1 | PKS\_I | BGC0000002 | Aculeximycin\_biosynthetic\_gene\_cluster | 45 | 36.8378812199 | 723 | 1.1e-207 |
| BAF85844.1 | modular\_polyketide\_synthase | BGC0000109 | Nemadectin\_biosynthetic\_gene\_cluster | 43 | 40.8908507223 | 722 | 1.4e-207 |
| BAJ16470.1 | polyketide\_synthase | BGC0000058 | FD-891\_biosynthetic\_gene\_cluster | 45 | 36.1958266453 | 722 | 1.8e-207 |
| BAD08360.1 | polyketide\_synthase\_modules\_7-8 | BGC0000167 | Vicenistatin\_biosynthetic\_gene\_cluster | 41 | 41.7335473515 | 721 | 4e-207 |
| ctg1\_orf28 |  | BGC0000096 | Midecamycin\_biosynthetic\_gene\_cluster | 43 | 39.0850722311 | 721 | 4e-207 |
| CCP20047.1 | divK\_protein | BGC0001119 | Divergolide\_biosynthetic\_gene\_cluster | 41 | 45.2247191011 | 720 | 5.3e-207 |
| AEK75502.1 | type\_1\_polyketide\_synthase | BGC0000001 | Abyssomicin\_biosynthetic\_gene\_cluster | 43 | 41.1316211878 | 720 | 5.3e-207 |
| BAB69196.1 | modular\_polyketide\_synthase | BGC0000117 | Oligomycin\_biosynthetic\_gene\_cluster | 41 | 42.7367576244 | 720 | 5.3e-207 |
| BAO66541.1 | type\_I\_polyketide\_synthase | BGC0000042 | Cremimycin\_biosynthetic\_gene\_cluster | 46 | 35.9149277689 | 720 | 6.9e-207 |
| AAG23265.1 | polyketide\_synthase\_extender\_module\_2 | BGC0000148 | Spinosad\_biosynthetic\_gene\_cluster | 43 | 36.2359550562 | 720 | 9e-207 |
| ABW96542.1 | type\_I\_modular\_polyketide\_synthase | BGC0000159 | Tautomycin\_biosynthetic\_gene\_cluster | 41 | 43.1781701445 | 719 | 1.2e-206 |
| BAD08373.1 | polyketide\_synthase\_modules\_1-3 | BGC0000167 | Vicenistatin\_biosynthetic\_gene\_cluster | 43 | 38.8443017657 | 719 | 1.2e-206 |
| ACO94456.1 | polyketide\_synthase\_type\_I | BGC0000029 | BE-14106\_biosynthetic\_gene\_cluster | 41 | 42.4959871589 | 719 | 1.5e-206 |
| BAC68128.1 | modular\_polyketide\_synthase | BGC0000059 | Filipin\_biosynthetic\_gene\_cluster | 44 | 37.27929374 | 719 | 1.5e-206 |
| CAC20919.1 | PimS4\_protein | BGC0000125 | Pimaricin\_biosynthetic\_gene\_cluster | 45 | 35.834670947 | 718 | 2e-206 |
| BAO66543.1 | type\_I\_polyketide\_synthase | BGC0000042 | Cremimycin\_biosynthetic\_gene\_cluster | 43 | 38.4430176565 | 718 | 2.6e-206 |
| AEP40939.1 | polyketide\_synthase\_type\_I | BGC0000021 | Apoptolidin\_biosynthetic\_gene\_cluster | 45 | 35.9951845907 | 718 | 2.6e-206 |
| ACN69988.1 | polyketide\_synthase | BGC0000079 | Indanomycin\_biosynthetic\_gene\_cluster | 41 | 40.6500802568 | 718 | 2.6e-206 |
| ACY06288.1 | type\_I\_polyketide\_synthase | BGC0001042 | Sanglifehrin\_A\_biosynthetic\_gene\_cluster | 42 | 40.9309791332 | 718 | 3.4e-206 |
| ABC84459.1 | NigAIV | BGC0000114 | Nigericin\_biosynthetic\_gene\_cluster | 43 | 40.2487961477 | 717 | 4.5e-206 |
| ADC45538.1 | modular\_polyketide\_synthase | BGC0000093 | Meilingmycin\_biosynthetic\_gene\_cluster | 41 | 41.0914927769 | 717 | 5.8e-206 |
| ADX66460.1 | ScnS3 | BGC0000108 | Natamycin\_biosynthetic\_gene\_cluster | 45 | 36.0353130016 | 717 | 5.8e-206 |
| AEZ54379.1 | PieA6 | BGC0000124 | Piericidin\_A1\_biosynthetic\_gene\_cluster | 42 | 40.8105939005 | 717 | 7.6e-206 |
| AEC13069.1 | fosC | BGC0000060 | Fostriecin\_biosynthetic\_gene\_cluster | 42 | 40.6099518459 | 716 | 1.3e-205 |
| CAC20920.1 | PimS3\_protein | BGC0000125 | Pimaricin\_biosynthetic\_gene\_cluster | 45 | 36.1155698234 | 716 | 1.3e-205 |
| AHH99919.1 | PKS\_I | BGC0000002 | Aculeximycin\_biosynthetic\_gene\_cluster | 45 | 35.9550561798 | 716 | 1.3e-205 |
| ABV91286.1 | type\_I\_modular\_polyketide\_synthase | BGC0000158 | Tautomycetin\_biosynthetic\_gene\_cluster | 43 | 39.7271268058 | 715 | 1.7e-205 |
| ADC45535.1 | modular\_polyketide\_synthase | BGC0000093 | Meilingmycin\_biosynthetic\_gene\_cluster | 44 | 37.0786516854 | 715 | 2.2e-205 |
| ACO94484.1 | polyketide\_synthase\_type\_I | BGC0000097 | ML-449\_biosynthetic\_gene\_cluster | 44 | 36.6773675762 | 715 | 2.9e-205 |
| AAQ82566.1 | FscF | BGC0000034 | Candicidin\_biosynthetic\_gene\_cluster | 44 | 35.7945425361 | 714 | 3.8e-205 |
| BAQ25483.1 | type\_I\_polyketide\_synthase | BGC0001288 | Maklamicin\_biosynthetic\_gene\_cluster | 43 | 37.4398073836 | 714 | 3.8e-205 |
| AEZ53947.1 | polyketide\_synthase | BGC0000144 | Salinomycin\_biosynthetic\_gene\_cluster | 40 | 43.1380417335 | 714 | 4.9e-205 |
| AJW65408.1 | type\_I\_modular\_polyketide\_synthase | BGC0001195 | Nocardiopsin\_biosynthetic\_gene\_cluster | 43 | 39.3659711075 | 713 | 6.5e-205 |
| BAR73020.1 | putative\_PKS\_(KS-AT-DH-KR-ACP-KS-AT-DH-KR-ACP-KS-AT-DH-KR-ACP) | BGC0001194 | Hitachimycin\_biosynthetic\_gene\_cluster | 45 | 35.9149277689 | 713 | 8.4e-205 |
| ABI94379.1 | tautomycetin\_biosynthetic\_PKS | BGC0000157 | Tautomycetin\_biosynthetic\_gene\_cluster | 43 | 39.7271268058 | 713 | 8.4e-205 |
| BAG85030.1 | putative\_polyketide\_synthase | BGC0000086 | Lasalocid\_biosynthetic\_gene\_cluster | 41 | 40.8507223114 | 713 | 1.1e-204 |
| CAQ64690.1 | lasalocid\_modular\_polyketide\_synthase | BGC0000087 | Lasalocid\_biosynthetic\_gene\_cluster | 41 | 40.8507223114 | 713 | 1.1e-204 |
| BAQ21946.1 | putative\_type\_I\_polyketide\_synthase | BGC0001204 | Versipelostatin\_biosynthetic\_gene\_cluster | 39 | 44.7431781701 | 713 | 1.1e-204 |
| AGY30677.1 | Ann5 | BGC0001298 | 4-Z-annimycin\_biosynthetic\_gene\_cluster | 43 | 37.9213483146 | 711 | 4.2e-204 |
| CQR60497.1 | Polyketide\_synthase,\_type\_I,\_modules:\_loading,\_1,\_2\_and\_3 | BGC0001287 | Chaxamycin\_A\_/\_chaxamycin\_B\_/\_chaxamycin\_C\_/\_chaxamycin\_D | 41 | 42.5762439807 | 711 | 4.2e-204 |
| ctg1\_orf11 |  | BGC0000053 | Elaiophylin\_biosynthetic\_gene\_cluster | 44 | 36.3964686998 | 711 | 4.2e-204 |
| ABB88535.1 | polyketide\_synthase\_type\_I | BGC0000049\_c2 | E-492\_/\_E-975\_biosynthetic\_gene\_cluster | 40 | 43.6597110754 | 710 | 5.5e-204 |
| AAC01710.1 | RifA | BGC0000136 | Rifamycin\_biosynthetic\_gene\_cluster | 35 | 61.9582664526 | 710 | 9.3e-204 |
| CAL58681.1 | polyketide\_synthase | BGC0000149 | Spirangien\_biosynthetic\_gene\_cluster | 42 | 38.5634028892 | 710 | 9.3e-204 |
| CAJ88176.1 | putative\_polyketide\_synthase\_B | BGC0000151 | Stambomycin\_biosynthetic\_gene\_cluster | 43 | 37.1187800963 | 709 | 1.2e-203 |
| ABW96540.1 | type\_I\_modular\_polyketide\_synthase | BGC0000159 | Tautomycin\_biosynthetic\_gene\_cluster | 42 | 39.165329053 | 708 | 2.1e-203 |
| ADX66459.1 | ScnS4 | BGC0000108 | Natamycin\_biosynthetic\_gene\_cluster | 45 | 35.7544141252 | 708 | 2.7e-203 |
| BAP34739.1 | type\_I\_polyketide\_synthase | BGC0000078 | Incednine\_biosynthetic\_gene\_cluster | 41 | 40.6902086677 | 707 | 4.6e-203 |
| BAF85843.1 | modular\_polyketide\_synthase | BGC0000109 | Nemadectin\_biosynthetic\_gene\_cluster | 41 | 40.1685393258 | 707 | 4.6e-203 |
| AFL48527.1 | laidlomycin\_polyketide\_synthase\_(module\_3\_and\_module\_4) | BGC0000084 | Laidlomycin\_biosynthetic\_gene\_cluster | 45 | 36.9181380417 | 706 | 1e-202 |
| ABI94380.1 | tautomycetin\_biosynthetic\_PKS | BGC0000157 | Tautomycetin\_biosynthetic\_gene\_cluster | 43 | 40.2086677368 | 706 | 1.3e-202 |
| CAQ64692.1 | lasalocid\_modular\_polyketide\_synthase | BGC0000087 | Lasalocid\_biosynthetic\_gene\_cluster | 44 | 36.6773675762 | 706 | 1.3e-202 |
| AFL48528.1 | laidlomycin\_polyketide\_synthase\_(module\_7\_and\_module\_8) | BGC0000084 | Laidlomycin\_biosynthetic\_gene\_cluster | 41 | 41.8940609952 | 705 | 1.8e-202 |
| ACR50785.1 | polyketide\_synthase | BGC0000163 | Tetronasin\_biosynthetic\_gene\_cluster | 41 | 39.5264847512 | 705 | 1.8e-202 |
| ALP32045.1 | CycE | BGC0001293 | Cyclizidine\_biosynthetic\_gene\_cluster | 42 | 40.569823435 | 705 | 1.8e-202 |
| AAQ84157.1 | Plm2-3 | BGC0000123 | Phoslactomycin\_B\_biosynthetic\_gene\_cluster | 42 | 40.0080256822 | 705 | 2.3e-202 |
| ACN69989.1 | polyketide\_synthase | BGC0000079 | Indanomycin\_biosynthetic\_gene\_cluster | 42 | 39.4863563403 | 705 | 2.3e-202 |
| AHE80995.1 | PieA5 | BGC0001169 | Piericidin\_A1\_biosynthetic\_gene\_cluster | 42 | 40.2487961477 | 705 | 2.3e-202 |
| AHE80992.1 | PieA2 | BGC0001169 | Piericidin\_A1\_biosynthetic\_gene\_cluster | 34 | 62.6003210273 | 705 | 3e-202 |
| AEC13071.1 | fosE | BGC0000060 | Fostriecin\_biosynthetic\_gene\_cluster | 42 | 40.9711075441 | 704 | 3.9e-202 |
| ABV91287.1 | type\_I\_modular\_polyketide\_synthase | BGC0000158 | Tautomycetin\_biosynthetic\_gene\_cluster | 42 | 39.4462279294 | 703 | 8.7e-202 |
| ACO94483.1 | polyketide\_synthase\_type\_I | BGC0000097 | ML-449\_biosynthetic\_gene\_cluster | 40 | 42.8571428571 | 703 | 8.7e-202 |
| CAC22144.1 | putative\_type\_I\_polyketide\_synthase | BGC0000038 | Coelimycin\_biosynthetic\_gene\_cluster | 44 | 35.9550561798 | 703 | 1.1e-201 |
| CAJ88187.2 | putative\_type\_I\_modular\_polyketide\_synthase | BGC0000151 | Stambomycin\_biosynthetic\_gene\_cluster | 45 | 36.0754414125 | 703 | 1.1e-201 |
| BAF85839.1 | modular\_polyketide\_synthase | BGC0000109 | Nemadectin\_biosynthetic\_gene\_cluster | 41 | 41.0112359551 | 701 | 3.3e-201 |
| ACR50782.1 | polyketide\_synthase | BGC0000163 | Tetronasin\_biosynthetic\_gene\_cluster | 42 | 36.6773675762 | 701 | 4.3e-201 |
| CAA60462.1 | polyketide\_synthase | BGC0001040 | Rapamycin\_biosynthetic\_gene\_cluster | 40 | 40.7303370787 | 700 | 5.7e-201 |
| CAD70195.1 | non-ribosomal\_peptide\_synthetase | BGC0001047 | Syringolin\_A\_biosynthetic\_gene\_cluster | 34 | 62.27929374 | 700 | 9.6e-201 |
| BAF85838.1 | modular\_polyketide\_synthase | BGC0000109 | Nemadectin\_biosynthetic\_gene\_cluster | 39 | 45.5457463884 | 699 | 1.3e-200 |
| AFL48529.1 | laidlomycin\_polyketide\_synthase\_(module\_5\_and\_module\_6) | BGC0000084 | Laidlomycin\_biosynthetic\_gene\_cluster | 42 | 39.9678972713 | 699 | 1.3e-200 |
| CAN89636.1 | putative\_polyketide\_synthase | BGC0001070 | Kirromycin\_biosynthetic\_gene\_cluster | 40 | 43.0577849117 | 699 | 1.3e-200 |
| BAP34734.1 | type\_I\_polyketide\_synthase | BGC0000078 | Incednine\_biosynthetic\_gene\_cluster | 41 | 41.6131621188 | 699 | 1.3e-200 |
| AFV30251.1 | polyketide\_synthase | BGC0000075 | Hygrocin\_biosynthetic\_gene\_cluster | 42 | 41.2520064205 | 699 | 1.6e-200 |
| ABW96541.1 | type\_I\_modular\_polyketide\_synthase | BGC0000159 | Tautomycin\_biosynthetic\_gene\_cluster | 41 | 39.7271268058 | 698 | 2.1e-200 |
| AAG13918.1 | megalomicin\_6-deoxyerythronolide\_B\_synthase\_2 | BGC0000092 | Megalomicin\_biosynthetic\_gene\_cluster | 34 | 59.9518459069 | 698 | 2.8e-200 |
| ACO94498.1 | polyketide\_synthase\_type\_I | BGC0000097 | ML-449\_biosynthetic\_gene\_cluster | 44 | 36.0754414125 | 698 | 3.7e-200 |
| AAQ84156.1 | Plm1 | BGC0000123 | Phoslactomycin\_B\_biosynthetic\_gene\_cluster | 40 | 42.8170144462 | 698 | 3.7e-200 |
| EJK79843.1 | amino\_acid\_adenylation\_enzyme/thioester\_reductase\_family\_protein | BGC0000436 | Syringolin\_A\_biosynthetic\_gene\_cluster | 34 | 61.1958266453 | 698 | 3.7e-200 |
| ADM46357.1 | polyketide\_synthase | BGC0000106 | Naphthomycin\_biosynthetic\_gene\_cluster | 41 | 41.5730337079 | 698 | 3.7e-200 |
| AJW65409.1 | type\_I\_modular\_polyketide\_synthase | BGC0001195 | Nocardiopsin\_biosynthetic\_gene\_cluster | 42 | 41.051364366 | 697 | 4.8e-200 |
| CAQ18834.1 | polyketide\_synthase | BGC0000954 | Ajudazol\_biosynthetic\_gene\_cluster | 44 | 36.1155698234 | 697 | 4.8e-200 |
| CCA29203.1 | non-ribosomal\_peptide\_synthetase/polyketide\_synthase | BGC0000955 | Althiomycin\_biosynthetic\_gene\_cluster | 33 | 63.202247191 | 697 | 6.3e-200 |
| CAL80821.1 | sylD-like\_NRPS/PKS | BGC0000997 | Glidobactin\_biosynthetic\_gene\_cluster | 33 | 62.3595505618 | 696 | 8.2e-200 |
| ctg1\_orf523 |  | BGC0001199 | Akaeolide\_biosynthetic\_gene\_cluster | 45 | 35.9550561798 | 696 | 1.1e-199 |
| AAO65799.1 | monensin\_polyketide\_synthase\_modules\_5\_and\_6 | BGC0000100 | Monensin\_biosynthetic\_gene\_cluster | 41 | 40.1284109149 | 696 | 1.1e-199 |
| ACN69990.1 | polyketide\_synthase | BGC0000079 | Indanomycin\_biosynthetic\_gene\_cluster | 44 | 36.0754414125 | 695 | 1.8e-199 |
| AAP42857.1 | NanA3 | BGC0000105 | Nanchangmycin\_biosynthetic\_gene\_cluster | 44 | 36.9983948636 | 695 | 1.8e-199 |
| AEU17897.1 | putative\_type\_I\_PKS | BGC0001072 | Simocyclinone\_biosynthetic\_gene\_cluster | 44 | 36.3563402889 | 695 | 2.4e-199 |
| BAC68127.1 | modular\_polyketide\_synthase | BGC0000059 | Filipin\_biosynthetic\_gene\_cluster | 44 | 36.3964686998 | 695 | 2.4e-199 |
| AEH42491.1 | polyketide\_synthase | BGC0000032 | Calcimycin\_biosynthetic\_gene\_cluster | 44 | 35.9550561798 | 695 | 3.1e-199 |
| BAR73021.1 | putative\_PKS\_(KS-AT-KR-ACP) | BGC0001194 | Hitachimycin\_biosynthetic\_gene\_cluster | 44 | 36.7174959872 | 695 | 3.1e-199 |
| BAD08359.1 | polyketide\_synthase\_modules\_5-6 | BGC0000167 | Vicenistatin\_biosynthetic\_gene\_cluster | 43 | 37.9213483146 | 695 | 3.1e-199 |
| AEK75504.1 | type\_1\_polyketide\_synthase | BGC0000001 | Abyssomicin\_biosynthetic\_gene\_cluster | 45 | 35.7945425361 | 695 | 3.1e-199 |
| AAO65800.1 | monensin\_polyketide\_synthase\_modules\_7\_and\_8 | BGC0000100 | Monensin\_biosynthetic\_gene\_cluster | 40 | 41.8940609952 | 694 | 5.3e-199 |
| CBZ41585.1 | putative\_modular\_polyketide\_synthase | BGC0000151 | Stambomycin\_biosynthetic\_gene\_cluster | 43 | 38.3627608347 | 694 | 5.3e-199 |
| ABV83222.1 | CppJ | BGC0000116 | Nystatin-like\_Pseudonocardia\_polyene\_biosynthetic\_gene\_cluster | 44 | 36.0353130016 | 694 | 5.3e-199 |
| BAR73019.1 | putative\_PKS\_(KS-AT-DH-KR-ACP-TE) | BGC0001194 | Hitachimycin\_biosynthetic\_gene\_cluster | 44 | 35.8747993579 | 693 | 6.9e-199 |
| ACN69991.1 | polyketide\_synthase | BGC0000079 | Indanomycin\_biosynthetic\_gene\_cluster | 44 | 36.1155698234 | 693 | 9e-199 |
| CQR60493.1 | Polyketide\_synthase,\_type\_I,\_modules:\_9\_and\_10 | BGC0001287 | Chaxamycin\_A\_/\_chaxamycin\_B\_/\_chaxamycin\_C\_/\_chaxamycin\_D | 42 | 40.0080256822 | 693 | 1.2e-198 |
| ACO94468.1 | polyketide\_synthase\_type\_I | BGC0000029 | BE-14106\_biosynthetic\_gene\_cluster | 42 | 38.3226324238 | 693 | 1.2e-198 |
| AEC13079.1 | fosA | BGC0000060 | Fostriecin\_biosynthetic\_gene\_cluster | 42 | 39.2857142857 | 693 | 1.2e-198 |
| AAT70107.1 | CurL | BGC0001165 | Curacin\_biosynthetic\_gene\_cluster | 41 | 37.2391653291 | 693 | 1.2e-198 |
| AKD43769.1 | HerA2 | BGC0001349 | Heronamide\_biosynthetic\_gene\_cluster | 45 | 36.3563402889 | 693 | 1.2e-198 |
| AEE88278.1 | CurL | BGC0000976 | Curacin\_biosynthetic\_gene\_cluster | 41 | 37.2391653291 | 693 | 1.2e-198 |
| BAK64638.1 | polyketide\_synthase | BGC0000135 | Reveromycin\_biosynthetic\_gene\_cluster | 41 | 40.569823435 | 692 | 1.5e-198 |
| ALA09358.1 | type\_I\_modular\_PKS | BGC0001303 | Lobosamide\_biosynthetic\_gene\_cluster | 43 | 38.9646869984 | 692 | 2e-198 |
| BAK64649.1 | polyketide\_synthase | BGC0000135 | Reveromycin\_biosynthetic\_gene\_cluster | 41 | 41.492776886 | 691 | 2.6e-198 |
| ACO94470.1 | polyketide\_synthase\_type\_I | BGC0000029 | BE-14106\_biosynthetic\_gene\_cluster | 43 | 37.6404494382 | 690 | 5.8e-198 |
| ABG02264.1 | SalB | BGC0000143 | Salinomycin\_biosynthetic\_gene\_cluster | 40 | 44.6227929374 | 690 | 7.6e-198 |
| ALP32046.1 | CycF | BGC0001293 | Cyclizidine\_biosynthetic\_gene\_cluster | 41 | 39.9678972713 | 689 | 1.3e-197 |
| CAE46843.1 | Type\_I\_modular\_polyketide\_synthase | BGC0000103 | Mycolactone\_biosynthetic\_gene\_cluster | 39 | 42.455858748 | 689 | 1.3e-197 |
| ALP32043.1 | CycC | BGC0001293 | Cyclizidine\_biosynthetic\_gene\_cluster | 41 | 40.2487961477 | 689 | 1.7e-197 |
| BAQ25481.1 | type\_I\_polyketide\_synthase | BGC0001288 | Maklamicin\_biosynthetic\_gene\_cluster | 40 | 42.8170144462 | 689 | 1.7e-197 |
| ACO94496.1 | polyketide\_synthase\_type\_I | BGC0000097 | ML-449\_biosynthetic\_gene\_cluster | 43 | 38.0818619583 | 688 | 2.2e-197 |
| BAG85032.1 | putative\_polyketide\_synthase | BGC0000086 | Lasalocid\_biosynthetic\_gene\_cluster | 43 | 36.316211878 | 688 | 2.2e-197 |
| AAC46024.1 | polyketide\_synthase\_modules\_1\_and\_2 | BGC0000113 | Niddamycin\_biosynthetic\_gene\_cluster | 42 | 39.4863563403 | 688 | 3.8e-197 |
| AAP42859.1 | NanA5 | BGC0000105 | Nanchangmycin\_biosynthetic\_gene\_cluster | 41 | 39.5666131621 | 688 | 3.8e-197 |
| AAP42858.1 | NanA4 | BGC0000105 | Nanchangmycin\_biosynthetic\_gene\_cluster | 42 | 39.5666131621 | 688 | 3.8e-197 |
| AEC13072.1 | fosF | BGC0000060 | Fostriecin\_biosynthetic\_gene\_cluster | 43 | 37.27929374 | 687 | 5e-197 |
| AJW65407.1 | type\_I\_modular\_polyketide\_synthase | BGC0001195 | Nocardiopsin\_biosynthetic\_gene\_cluster | 40 | 42.1348314607 | 687 | 5e-197 |
| ACO94499.1 | polyketide\_synthase\_type\_I | BGC0000097 | ML-449\_biosynthetic\_gene\_cluster | 42 | 38.683788122 | 687 | 5e-197 |
| ACF35445.1 | MbcAI | BGC0000090 | Macbecin\_biosynthetic\_gene\_cluster | 42 | 39.165329053 | 687 | 6.5e-197 |
| AAS79461.1 | polyketide\_synthase\_subunit | BGC0000035 | Chalcomycin\_biosynthetic\_gene\_cluster | 36 | 55.9390048154 | 687 | 6.5e-197 |
| BAC57032.1 | protomycinolide\_IV\_synthase\_5 | BGC0000102 | Mycinamicin\_biosynthetic\_gene\_cluster | 43 | 35.9951845907 | 686 | 1.1e-196 |
| ACR50775.1 | polyketide\_synthase | BGC0000163 | Tetronasin\_biosynthetic\_gene\_cluster | 41 | 37.4799357945 | 686 | 1.1e-196 |
| ALD82522.1 | polyketide\_synthase | BGC0001212 | Nannocystin\_biosynthetic\_gene\_cluster | 42 | 39.1252006421 | 686 | 1.1e-196 |
| CCP20049.1 | divL2\_protein | BGC0001119 | Divergolide\_biosynthetic\_gene\_cluster | 44 | 36.7576243981 | 686 | 1.4e-196 |
| AJO72737.1 | Type\_I\_modular\_polyketide\_synthase | BGC0001381 | Brasilinolide\_biosynthetic\_gene\_cluster | 40 | 42.3756019262 | 686 | 1.4e-196 |
| CBA11584.1 | polyketide\_synthase\_type\_I | BGC0001046 | Streptolydigin\_biosynthetic\_gene\_cluster | 41 | 40.3290529695 | 685 | 1.9e-196 |
| CAE46851.1 | Type\_I\_modular\_polyketide\_synthase | BGC0000103 | Mycolactone\_biosynthetic\_gene\_cluster | 40 | 40.2086677368 | 685 | 1.9e-196 |
| ABC84458.1 | NigAIII | BGC0000114 | Nigericin\_biosynthetic\_gene\_cluster | 42 | 37.27929374 | 685 | 1.9e-196 |
| ADI59531.1 | CorI | BGC0001091 | Corallopyronin\_biosynthetic\_gene\_cluster | 37 | 46.227929374 | 685 | 3.2e-196 |
| AAQ84147.1 | Plm7 | BGC0000123 | Phoslactomycin\_B\_biosynthetic\_gene\_cluster | 44 | 37.5200642055 | 684 | 4.2e-196 |
| ABS90470.1 | NRPS/PKS | BGC0001106 | Oxazolomycin\_biosynthetic\_gene\_cluster | 38 | 47.632423756 | 684 | 4.2e-196 |
| ALA09357.1 | type\_I\_modular\_PKS | BGC0001303 | Lobosamide\_biosynthetic\_gene\_cluster | 41 | 41.1316211878 | 684 | 5.5e-196 |
| ABG02263.1 | SalA | BGC0000143 | Salinomycin\_biosynthetic\_gene\_cluster | 40 | 43.0577849117 | 684 | 5.5e-196 |
| ADM46358.1 | polyketide\_synthase | BGC0000106 | Naphthomycin\_biosynthetic\_gene\_cluster | 41 | 40.2086677368 | 684 | 5.5e-196 |
| AAU93806.2 | polyketide\_synthase\_modules\_3\_and\_4 | BGC0000054 | Erythromycin\_biosynthetic\_gene\_cluster | 35 | 60.2728731942 | 684 | 5.5e-196 |
| AKD43761.1 | HerD | BGC0001349 | Heronamide\_biosynthetic\_gene\_cluster | 40 | 44.1011235955 | 683 | 7.2e-196 |
| ADB12493.1 | EpoF | BGC0000990 | Epothilone\_biosynthetic\_gene\_cluster | 43 | 35.8747993579 | 683 | 9.3e-196 |
| BAQ25512.1 | type\_I\_polyketide\_synthase | BGC0001288 | Maklamicin\_biosynthetic\_gene\_cluster | 41 | 39.8475120385 | 683 | 1.2e-195 |
| ADC45586.1 | modular\_polyketide\_synthase | BGC0000093 | Meilingmycin\_biosynthetic\_gene\_cluster | 40 | 42.6163723917 | 682 | 1.6e-195 |
| CBZ41586.1 | putative\_modular\_polyketide\_synthase | BGC0000151 | Stambomycin\_biosynthetic\_gene\_cluster | 44 | 35.7544141252 | 682 | 2.1e-195 |
| ABP55223.1 | beta-ketoacyl\_synthase | BGC0000142 | Salinilactam\_biosynthetic\_gene\_cluster | 41 | 40.8105939005 | 682 | 2.1e-195 |
| AAC46025.1 | polyketide\_synthase\_module\_3 | BGC0000113 | Niddamycin\_biosynthetic\_gene\_cluster | 43 | 36.6372391653 | 681 | 3.5e-195 |
| AAM54076.1 | polyketide\_synthase | BGC0000020 | Ansamitocin\_biosynthetic\_gene\_cluster | 40 | 44.0609951846 | 681 | 3.5e-195 |
| AAF86396.1 | FkbA | BGC0000994 | FK520\_biosynthetic\_gene\_cluster | 40 | 40.8507223114 | 681 | 4.6e-195 |
| CAE14173.1 |  | BGC0000383 | Luminmycin\_biosynthetic\_gene\_cluster | 32 | 62.8812199037 | 680 | 6.1e-195 |
| AAM81586.2 | putative\_type\_I\_polyketide\_synthase | BGC0000047 | Dihydrochalcomycin\_biosynthetic\_gene\_cluster | 35 | 56.0192616372 | 680 | 6.1e-195 |
| ADM46359.1 | polyketide\_synthase | BGC0000106 | Naphthomycin\_biosynthetic\_gene\_cluster | 42 | 39.0850722311 | 680 | 7.9e-195 |
| BAP05590.1 | CalB | BGC0000967 | Calyculin\_biosynthetic\_gene\_cluster | 37 | 46.5890850722 | 680 | 1e-194 |
| ctg1\_orf524 |  | BGC0001199 | Akaeolide\_biosynthetic\_gene\_cluster | 44 | 36.0754414125 | 679 | 1.3e-194 |
| CAE45669.1 | borrelidin\_polyketide\_synthase,\_type\_I | BGC0000031 | Borrelidin\_biosynthetic\_gene\_cluster | 44 | 35.9149277689 | 679 | 1.8e-194 |
| ABB88520.1 | polyketide\_synthase\_type\_I | BGC0000050 | E-837\_biosynthetic\_gene\_cluster | 44 | 35.834670947 | 679 | 1.8e-194 |
| AAF26923.1 | polyketide\_synthase | BGC0000988 | Epothilone\_biosynthetic\_gene\_cluster | 44 | 35.8747993579 | 678 | 2.3e-194 |
| BAC57029.1 | protomycinolide\_IV\_synthase\_2 | BGC0000102 | Mycinamicin\_biosynthetic\_gene\_cluster | 44 | 36.5168539326 | 678 | 2.3e-194 |
| AAF62885.1 | EpoF | BGC0000991 | Epothilone\_biosynthetic\_gene\_cluster | 43 | 35.8747993579 | 678 | 3e-194 |
| AEE88281.1 | CurI | BGC0000976 | Curacin\_biosynthetic\_gene\_cluster | 41 | 37.1990369181 | 678 | 3e-194 |
| AAT70104.1 | CurI | BGC0001165 | Curacin\_biosynthetic\_gene\_cluster | 41 | 37.1990369181 | 678 | 3e-194 |
| AFL48520.1 | laidlomycin\_polyketide\_synthase\_(module\_9) | BGC0000084 | Laidlomycin\_biosynthetic\_gene\_cluster | 42 | 37.72070626 | 678 | 3.9e-194 |
| AAB66506.1 | tylactone\_synthase\_modules\_4\_&\_5 | BGC0000166 | Tylactone\_biosynthetic\_gene\_cluster | 33 | 62.4799357945 | 676 | 8.7e-194 |
| AAF71768.1 | NysK | BGC0000115 | Nystatin\_biosynthetic\_gene\_cluster | 43 | 36.4365971108 | 676 | 8.7e-194 |
| AAK73503.1 | AmphK | BGC0000015 | Amphotericin\_biosynthetic\_gene\_cluster | 42 | 35.9149277689 | 676 | 1.1e-193 |
| BAT51067.1 | type\_I\_polyketide\_synthase | BGC0001296 | Streptazone\_E\_biosynthetic\_gene\_cluster | 41 | 38.0016051364 | 676 | 1.1e-193 |
| ABM21570.1 | CrpB | BGC0000975 | Cryptophycin\_biosynthetic\_gene\_cluster | 39 | 38.8041733547 | 676 | 1.1e-193 |
| AHH99924.1 | PKS\_I | BGC0000002 | Aculeximycin\_biosynthetic\_gene\_cluster | 44 | 36.3964686998 | 676 | 1.5e-193 |
| CDG12864.1 | non-ribosomal\_peptide\_synthetase | BGC0001415 | Althiomycin\_biosynthetic\_gene\_cluster | 33 | 59.1091492777 | 675 | 2.5e-193 |
| AAY28225.1 | HbmAI | BGC0000074 | Herbimycin\_biosynthetic\_gene\_cluster | 39 | 39.0850722311 | 675 | 2.5e-193 |
| ADM46360.1 | polyketide\_synthase | BGC0000106 | Naphthomycin\_biosynthetic\_gene\_cluster | 44 | 36.1958266453 | 675 | 3.3e-193 |
| AEC13080.1 | fosB | BGC0000060 | Fostriecin\_biosynthetic\_gene\_cluster | 42 | 36.7576243981 | 674 | 4.3e-193 |
| AFV30249.1 | polyketide\_synthase | BGC0000075 | Hygrocin\_biosynthetic\_gene\_cluster | 43 | 36.316211878 | 674 | 4.3e-193 |
| ACO94471.1 | polyketide\_synthase\_type\_I | BGC0000029 | BE-14106\_biosynthetic\_gene\_cluster | 42 | 38.6035313002 | 674 | 5.7e-193 |
| ACB46197.1 | polyketide\_synthase | BGC0000989 | Epothilone\_biosynthetic\_gene\_cluster | 43 | 35.8747993579 | 673 | 7.4e-193 |
| ABI93779.1 | GdmPKS | BGC0000068 | Geldanamycin\_biosynthetic\_gene\_cluster | 39 | 41.2118780096 | 673 | 9.7e-193 |
| AEE88282.1 | CurH | BGC0000976 | Curacin\_biosynthetic\_gene\_cluster | 41 | 36.2359550562 | 673 | 1.3e-192 |
| AJW65410.1 | type\_I\_modular\_polyketide\_synthase | BGC0001195 | Nocardiopsin\_biosynthetic\_gene\_cluster | 43 | 36.1556982343 | 673 | 1.3e-192 |
| AAT70103.1 | CurH | BGC0001165 | Curacin\_biosynthetic\_gene\_cluster | 41 | 36.2359550562 | 673 | 1.3e-192 |
| CAQ18828.1 | polyketide\_synthase | BGC0000954 | Ajudazol\_biosynthetic\_gene\_cluster | 43 | 35.9149277689 | 673 | 1.3e-192 |
| AFU82616.1 | polyketide\_synthase | BGC0000998 | Guadinomine\_biosynthetic\_gene\_cluster | 43 | 35.9550561798 | 672 | 1.6e-192 |
| ABP55220.1 | beta-ketoacyl\_synthase | BGC0000142 | Salinilactam\_biosynthetic\_gene\_cluster | 42 | 39.0850722311 | 672 | 1.6e-192 |
| ctg1\_orf522 |  | BGC0001199 | Akaeolide\_biosynthetic\_gene\_cluster | 43 | 36.0754414125 | 671 | 3.7e-192 |
| ACB46488.1 | polyketide\_synthase | BGC0000082 | Kijanimicin\_biosynthetic\_gene\_cluster | 40 | 40.8507223114 | 670 | 8.2e-192 |
| AAO06916.1 | GdmAI | BGC0000066 | Geldanamycin\_biosynthetic\_gene\_cluster | 39 | 39.0850722311 | 669 | 1.4e-191 |
| BAP34763.1 | type\_I\_polyketide\_synthase | BGC0000078 | Incednine\_biosynthetic\_gene\_cluster | 42 | 37.0385232745 | 669 | 1.8e-191 |
| AAO65801.1 | monensin\_polyketide\_synthase\_module\_9 | BGC0000100 | Monensin\_biosynthetic\_gene\_cluster | 42 | 36.3563402889 | 668 | 2.4e-191 |
| ACO94472.1 | polyketide\_synthase\_type\_I | BGC0000029 | BE-14106\_biosynthetic\_gene\_cluster | 43 | 37.7608346709 | 668 | 2.4e-191 |
| CAJ76298.1 | putative\_hybrid\_polyketide-non-ribosomal\_peptide\_synthetase | BGC0000972 | Colibactin\_biosynthetic\_gene\_cluster | 33 | 60.4735152488 | 668 | 2.4e-191 |
| CAE46850.1 | Type\_I\_modular\_polyketide\_synthase | BGC0000103 | Mycolactone\_biosynthetic\_gene\_cluster | 41 | 36.9181380417 | 668 | 2.4e-191 |
| CAD19092.1 | StiH\_protein | BGC0000153 | Stigmatellin\_biosynthetic\_gene\_cluster | 41 | 35.9149277689 | 668 | 3.1e-191 |
| ABV83223.1 | CppK | BGC0000116 | Nystatin-like\_Pseudonocardia\_polyene\_biosynthetic\_gene\_cluster | 44 | 36.1155698234 | 668 | 3.1e-191 |
| ALP32042.1 | CycB | BGC0001293 | Cyclizidine\_biosynthetic\_gene\_cluster | 43 | 36.4365971108 | 668 | 3.1e-191 |
| BAP34733.1 | type\_I\_polyketide\_synthase | BGC0000078 | Incednine\_biosynthetic\_gene\_cluster | 39 | 43.8202247191 | 667 | 5.3e-191 |
| ABB86408.1 | GelA | BGC0000067 | Geldanamycin\_biosynthetic\_gene\_cluster | 39 | 39.0850722311 | 667 | 5.3e-191 |
| AHB82064.1 | polyketide\_synthase | BGC0001231 | Microsclerodermins\_biosynthetic\_gene\_cluster | 43 | 36.1958266453 | 667 | 5.3e-191 |
| BAQ25513.1 | type\_I\_polyketide\_synthase | BGC0001288 | Maklamicin\_biosynthetic\_gene\_cluster | 42 | 36.7576243981 | 667 | 6.9e-191 |
| ABV97155.1 | Acyl\_transferase | BGC0000137 | Rifamycin\_biosynthetic\_gene\_cluster | 44 | 36.3964686998 | 666 | 1.5e-190 |
| AAD03048.1 | type\_I\_polyketide\_synthase | BGC0000041 | Coronafacic\_acid\_biosynthetic\_gene\_cluster | 41 | 36.1958266453 | 665 | 2e-190 |
| AKD43763.1 | HerG | BGC0001349 | Heronamide\_biosynthetic\_gene\_cluster | 43 | 35.9550561798 | 665 | 3.4e-190 |
| ADF88262.1 | mixed\_nonribosomal\_peptide\_synthetase/\_polyketide\_synthase | BGC0000979 | Cylindrospermopsin\_biosynthetic\_gene\_cluster | 36 | 49.3980738363 | 664 | 4.5e-190 |
| ADH04657.1 | TugA | BGC0001342 | Thuggacin\_biosynthetic\_gene\_cluster | 39 | 43.0577849117 | 663 | 7.7e-190 |
| ACO94500.1 | polyketide\_synthase\_type\_I | BGC0000097 | ML-449\_biosynthetic\_gene\_cluster | 42 | 37.7608346709 | 663 | 1e-189 |
| AAT70105.1 | CurJ | BGC0001165 | Curacin\_biosynthetic\_gene\_cluster | 40 | 37.1990369181 | 663 | 1.3e-189 |
| AEE88280.1 | CurJ | BGC0000976 | Curacin\_biosynthetic\_gene\_cluster | 40 | 37.1990369181 | 663 | 1.3e-189 |
| ADF88265.1 | mixed\_nonribosomal\_peptide\_synthetase/\_polyketide\_synthase | BGC0000980 | Cylindrospermopsin\_biosynthetic\_gene\_cluster | 36 | 49.3980738363 | 663 | 1.3e-189 |
| ALA09354.1 | type\_I\_modular\_PKS | BGC0001303 | Lobosamide\_biosynthetic\_gene\_cluster | 41 | 37.5200642055 | 663 | 1.3e-189 |
| AAS98783.1 | JamL | BGC0001001 | Jamaicamide\_biosynthetic\_gene\_cluster | 40 | 36.4365971108 | 662 | 2.2e-189 |
| AGI99497.1 | type\_I\_polyketide\_synthase | BGC0001004 | Lobophorin\_biosynthetic\_gene\_cluster | 39 | 41.1717495987 | 661 | 2.9e-189 |
| AHA12079.1 | polyketide\_synthase\_type\_1 | BGC0001172 | Chlorizidine\_A\_biosynthetic\_gene\_cluster | 41 | 35.8747993579 | 661 | 2.9e-189 |
| ADB12488.1 | EpoA | BGC0000990 | Epothilone\_biosynthetic\_gene\_cluster | 43 | 35.9149277689 | 661 | 3.8e-189 |
| CAD19087.1 | StiC\_protein | BGC0000153 | Stigmatellin\_biosynthetic\_gene\_cluster | 41 | 35.9951845907 | 661 | 5e-189 |
| CAD19090.1 | StiF\_protein | BGC0000153 | Stigmatellin\_biosynthetic\_gene\_cluster | 41 | 37.1187800963 | 660 | 6.5e-189 |
| AIW82282.1 | PuwE | BGC0001125 | Puwainaphycins\_biosynthetic\_gene\_cluster | 40 | 36.9181380417 | 660 | 6.5e-189 |
| CAQ52623.1 | type\_I\_polyketide\_synthase,\_module\_6 | BGC0001066 | Kendomycin\_biosynthetic\_gene\_cluster | 41 | 36.1556982343 | 659 | 1.1e-188 |
| AGC09485.1 | LobS2 | BGC0001183 | Lobophorin\_biosynthetic\_gene\_cluster | 40 | 42.6966292135 | 659 | 1.4e-188 |
| AAC46026.1 | polyketide\_synthase\_modules\_4\_and\_5 | BGC0000113 | Niddamycin\_biosynthetic\_gene\_cluster | 43 | 36.1958266453 | 659 | 1.4e-188 |
| AAN32979.1 | BarE | BGC0000962 | Barbamide\_biosynthetic\_gene\_cluster | 31 | 64.0850722311 | 659 | 1.9e-188 |
| AAF62880.1 | EpoA | BGC0000991 | Epothilone\_biosynthetic\_gene\_cluster | 43 | 35.9149277689 | 659 | 1.9e-188 |
| AAF26919.1 | polyketide\_synthase | BGC0000988 | Epothilone\_biosynthetic\_gene\_cluster | 43 | 35.834670947 | 658 | 2.5e-188 |
| CAQ52622.1 | type\_I\_polyketide\_synthase,\_modules\_4-5 | BGC0001066 | Kendomycin\_biosynthetic\_gene\_cluster | 40 | 39.5666131621 | 658 | 2.5e-188 |
| AMB48442.1 | polyketide\_synthase | BGC0001357 | Carbamidocyclophane\_biosynthetic\_gene\_cluster | 41 | 35.7945425361 | 658 | 3.2e-188 |
| ACB37755.1 | putative\_type\_I\_polyketide\_synthase | BGC0000162 | Tetrocarcin\_A\_biosynthetic\_gene\_cluster | 40 | 39.0449438202 | 658 | 4.2e-188 |
| CBD77748.1 | polyketide\_synthase | BGC0000974 | Crocacin\_biosynthetic\_gene\_cluster | 42 | 35.8747993579 | 658 | 4.2e-188 |
| AEU11006.1 | NpnB | BGC0001029 | Nostophycin\_biosynthetic\_gene\_cluster | 40 | 35.8747993579 | 657 | 5.5e-188 |
| CQR60496.1 | Polyketide\_synthase,\_type\_I,\_modules:\_4,\_5\_and\_6 | BGC0001287 | Chaxamycin\_A\_/\_chaxamycin\_B\_/\_chaxamycin\_C\_/\_chaxamycin\_D | 39 | 42.6565008026 | 657 | 5.5e-188 |
| AID65222.1 | putative\_aspartate\_racemase | BGC0000335 | Cystomanamides\_biosynthetic\_gene\_cluster | 36 | 49.8796147673 | 657 | 7.2e-188 |
| AKD43764.1 | HerF | BGC0001349 | Heronamide\_biosynthetic\_gene\_cluster | 42 | 39.0048154093 | 657 | 7.2e-188 |
| CAL58682.1 | polyketide\_synthase | BGC0000149 | Spirangien\_biosynthetic\_gene\_cluster | 41 | 37.1990369181 | 657 | 7.2e-188 |
| ABB88534.1 | polyketide\_synthase\_type\_I | BGC0000049 | E-492\_/\_E-975\_biosynthetic\_gene\_cluster | 42 | 36.6372391653 | 656 | 9.4e-188 |
| ACB46192.1 | polyketide\_synthase | BGC0000989 | Epothilone\_biosynthetic\_gene\_cluster | 43 | 35.9149277689 | 656 | 1.2e-187 |
| AAO65798.1 | monensin\_polyketide\_synthase\_modules\_3\_and\_4 | BGC0000100 | Monensin\_biosynthetic\_gene\_cluster | 42 | 37.7608346709 | 656 | 1.2e-187 |
| AAT70108.1 | CurM | BGC0001165 | Curacin\_biosynthetic\_gene\_cluster | 41 | 36.9983948636 | 656 | 1.2e-187 |
| AEE88277.1 | CurM | BGC0000976 | Curacin\_biosynthetic\_gene\_cluster | 41 | 36.9983948636 | 656 | 1.2e-187 |
| ACV42478.1 | polyketide\_synthase | BGC0000043 | Curacin\_A\_biosynthetic\_gene\_cluster | 41 | 36.9983948636 | 656 | 1.2e-187 |
| AGC09484.1 | LobS1 | BGC0001183 | Lobophorin\_biosynthetic\_gene\_cluster | 39 | 40.5296950241 | 656 | 1.6e-187 |
| ctg1\_orf521 |  | BGC0001199 | Akaeolide\_biosynthetic\_gene\_cluster | 39 | 39.8475120385 | 655 | 2.1e-187 |
| CAI94713.1 | putative\_polyketide\_synthase | BGC0000141 | Rubradirin\_biosynthetic\_gene\_cluster | 39 | 42.937399679 | 655 | 2.7e-187 |
| AFL48525.1 | laidlomycin\_polyketide\_synthase\_(loading\_module\_and\_module\_1) | BGC0000084 | Laidlomycin\_biosynthetic\_gene\_cluster | 39 | 40.4093097913 | 655 | 2.7e-187 |
| ACB46471.1 | polyketide\_synthase | BGC0000082 | Kijanimicin\_biosynthetic\_gene\_cluster | 40 | 39.6468699839 | 654 | 3.6e-187 |
| AAS98782.1 | JamK | BGC0001001 | Jamaicamide\_biosynthetic\_gene\_cluster | 42 | 34.5906902087 | 654 | 6.1e-187 |
| CAQ18839.1 | hybrid\_polyketide\_synthase/nonribosomal\_polypetide\_synthetase | BGC0000954 | Ajudazol\_biosynthetic\_gene\_cluster | 38 | 43.41894061 | 653 | 1e-186 |
| CAO85896.1 | protein\_modular\_polyketide\_synthase\_NorA' | BGC0000110 | Neoaureothin\_biosynthetic\_gene\_cluster | 39 | 41.6131621188 | 653 | 1e-186 |
| BAQ21940.1 | putative\_Type\_I\_polyketide\_synthase | BGC0001204 | Versipelostatin\_biosynthetic\_gene\_cluster | 39 | 43.1781701445 | 653 | 1e-186 |
| CAA60459.1 | polyketide\_synthase | BGC0001040 | Rapamycin\_biosynthetic\_gene\_cluster | 39 | 42.0545746388 | 653 | 1.4e-186 |
| CBA11583.1 | polyketide\_synthase\_type\_I | BGC0001046 | Streptolydigin\_biosynthetic\_gene\_cluster | 40 | 40.3290529695 | 653 | 1.4e-186 |
| AGI61658.1 | modular\_PKS | BGC0000095\_c3 | Micromonolactam\_biosynthetic\_gene\_cluster | 43 | 35.9149277689 | 653 | 1.4e-186 |
| AGC24270.1 | PrlP | BGC0001038 | Pyralomicin\_biosynthetic\_gene\_cluster | 43 | 35.9951845907 | 652 | 2.3e-186 |
| BAB69195.1 | modular\_polyketide\_synthase | BGC0000117 | Oligomycin\_biosynthetic\_gene\_cluster | 40 | 40.6099518459 | 651 | 3e-186 |
| CAD19086.1 | StiB\_protein | BGC0000153 | Stigmatellin\_biosynthetic\_gene\_cluster | 40 | 35.834670947 | 651 | 5.1e-186 |
| CUW01171.1 | Type\_I\_polyketide\_synthase | BGC0001359 | PM100117\_/\_PM100118\_biosynthetic\_gene\_cluster | 38 | 42.1749598716 | 650 | 6.7e-186 |
| AAC01714.1 | RifE | BGC0000136 | Rifamycin\_biosynthetic\_gene\_cluster | 42 | 37.0786516854 | 650 | 8.8e-186 |
| ACR33077.1 | polyketide\_synthase | BGC0000017 | Anatoxin\_biosynthetic\_gene\_cluster | 38 | 38.2825040128 | 650 | 8.8e-186 |
| ACN69992.1 | polyketide\_synthase | BGC0000079 | Indanomycin\_biosynthetic\_gene\_cluster | 41 | 38.2423756019 | 648 | 3.3e-185 |
| CAD19093.1 | StiJ\_protein | BGC0000153 | Stigmatellin\_biosynthetic\_gene\_cluster | 40 | 36.7174959872 | 648 | 4.3e-185 |
| AEZ54374.1 | PieA1 | BGC0000124 | Piericidin\_A1\_biosynthetic\_gene\_cluster | 39 | 38.8844301766 | 648 | 4.3e-185 |
| AAV97877.1 | OnnI | BGC0001105 | Onnamide\_biosynthetic\_gene\_cluster | 34 | 46.3884430177 | 648 | 4.3e-185 |
| AEU11005.1 | NpnA | BGC0001029 | Nostophycin\_biosynthetic\_gene\_cluster | 41 | 35.9149277689 | 647 | 5.7e-185 |
| CCE88376.1 | polyketide\_synthase | BGC0001034 | Pellasoren\_biosynthetic\_gene\_cluster | 38 | 41.6532905297 | 647 | 7.4e-185 |
| BAE93731.1 | type\_I\_polyketide\_synthase | BGC0000164 | Tetronomycin\_biosynthetic\_gene\_cluster | 41 | 36.3964686998 | 646 | 1.3e-184 |
| IF55\_RS32375 | beta-ketoacyl\_synthase | BGC0001348 | JBIR-100\_biosynthetic\_gene\_cluster | 42 | 35.9951845907 | 645 | 2.2e-184 |
| BAF02927.1 | type\_I\_polyketide\_synthase | BGC0000073 | Halstoctacosanolide\_biosynthetic\_gene\_cluster | 40 | 36.6773675762 | 645 | 2.8e-184 |
| ADZ24997.1 | polyketide\_synthase | BGC0000380 | Leupyrrin\_biosynthetic\_gene\_cluster | 41 | 37.2391653291 | 645 | 2.8e-184 |
| AAP42855.1 | NanA1 | BGC0000105 | Nanchangmycin\_biosynthetic\_gene\_cluster | 38 | 41.4125200642 | 644 | 3.7e-184 |
| AAP42860.1 | NanA6 | BGC0000105 | Nanchangmycin\_biosynthetic\_gene\_cluster | 41 | 38.683788122 | 644 | 3.7e-184 |
| AGC09499.1 | LobS4 | BGC0001183 | Lobophorin\_biosynthetic\_gene\_cluster | 40 | 39.8475120385 | 644 | 3.7e-184 |
| CCA89326.1 | mixed\_trans-AT\_type\_I\_polyketide\_synthase/nonribosomal\_peptide\_synthetase | BGC0001111 | Rhizopodins\_biosynthetic\_gene\_cluster | 36 | 49.8796147673 | 644 | 6.3e-184 |
| AAP42874.1 | NanA8 | BGC0000105 | Nanchangmycin\_biosynthetic\_gene\_cluster | 38 | 41.8138041734 | 643 | 8.2e-184 |
| ACY13414.1 | amino\_acid\_adenylation\_domain\_protein | BGC0001367 | Haliamide\_biosynthetic\_gene\_cluster | 40 | 41.532905297 | 643 | 1.1e-183 |
| ACF35447.1 | MbcAIII | BGC0000090 | Macbecin\_biosynthetic\_gene\_cluster | 40 | 39.3258426966 | 643 | 1.1e-183 |
| ABC84456.1 | NigAI | BGC0000114 | Nigericin\_biosynthetic\_gene\_cluster | 39 | 39.9678972713 | 643 | 1.4e-183 |
| AAF00959.1 | McyD | BGC0001017 | Microcystin\_biosynthetic\_gene\_cluster | 39 | 40.1685393258 | 642 | 1.8e-183 |
| AAM54075.1 | polyketide\_synthase | BGC0000020 | Ansamitocin\_biosynthetic\_gene\_cluster | 38 | 48.3547351525 | 642 | 1.8e-183 |
| ABV83228.1 | CppA | BGC0000116 | Nystatin-like\_Pseudonocardia\_polyene\_biosynthetic\_gene\_cluster | 42 | 35.9149277689 | 642 | 1.8e-183 |
| CAA60460.1 | polyketide\_synthase | BGC0001040 | Rapamycin\_biosynthetic\_gene\_cluster | 40 | 42.4959871589 | 642 | 2.4e-183 |
| BAE93725.1 | type\_I\_polyketide\_synthase | BGC0000164 | Tetronomycin\_biosynthetic\_gene\_cluster | 42 | 36.316211878 | 641 | 3.1e-183 |
| BAE93730.1 | type\_I\_polyketide\_synthase | BGC0000164 | Tetronomycin\_biosynthetic\_gene\_cluster | 39 | 39.4462279294 | 641 | 4.1e-183 |
| ACF35446.1 | MbcAII | BGC0000090 | Macbecin\_biosynthetic\_gene\_cluster | 42 | 36.0754414125 | 641 | 5.3e-183 |
| AAO65796.1 | monensin\_polyketide\_synthase\_loading\_module\_and\_module\_1 | BGC0000100 | Monensin\_biosynthetic\_gene\_cluster | 39 | 38.9646869984 | 641 | 5.3e-183 |
| CAO98879.1 | polyketide\_synthase\_AufD | BGC0000023 | Aurafuron\_biosynthetic\_gene\_cluster | 36 | 45.1444622793 | 640 | 6.9e-183 |
| CAE02602.1 | polyketide\_synthase\_type\_I | BGC0000024 | Aureothin\_biosynthetic\_gene\_cluster | 40 | 38.202247191 | 640 | 6.9e-183 |
| AFI57006.1 | QmnA2 | BGC0000133 | Quartromicin\_biosynthetic\_gene\_cluster | 41 | 36.0754414125 | 640 | 6.9e-183 |
| IF55\_RS36525 | polyketide\_synthase | BGC0001348 | JBIR-100\_biosynthetic\_gene\_cluster | 41 | 36.1155698234 | 640 | 9.1e-183 |
| ABV97152.1 | Beta-ketoacyl\_synthase | BGC0000137 | Rifamycin\_biosynthetic\_gene\_cluster | 39 | 40.1284109149 | 639 | 1.2e-182 |
| WP\_043476516.1 | beta-ketoacyl\_synthase | BGC0001232 | Leucanicidin\_biosynthetic\_gene\_cluster | 41 | 35.8747993579 | 639 | 1.2e-182 |
| AFV96138.1 | polyketide\_synthase | BGC0001064 | Cylindrocyclophane\_biosynthetic\_gene\_cluster | 40 | 35.9149277689 | 638 | 2.6e-182 |
| AAX98187.1 | polyketide\_synthase\_type\_I | BGC0000052 | ECO-02301\_biosynthetic\_gene\_cluster | 41 | 36.3964686998 | 638 | 3.4e-182 |
| AAC01711.1 | RifB | BGC0000136 | Rifamycin\_biosynthetic\_gene\_cluster | 38 | 40.8105939005 | 638 | 4.5e-182 |
| AEC13070.1 | fosD | BGC0000060 | Fostriecin\_biosynthetic\_gene\_cluster | 40 | 36.1556982343 | 637 | 7.7e-182 |
| ADC79638.1 | TamAII | BGC0001052 | Tirandamycin\_biosynthetic\_gene\_cluster | 39 | 41.2118780096 | 637 | 7.7e-182 |
| CAO85893.1 | modular\_polyketide\_synthase\_NorA | BGC0000110 | Neoaureothin\_biosynthetic\_gene\_cluster | 40 | 37.6805778491 | 637 | 7.7e-182 |
| ACR33078.1 | polyketide\_synthase | BGC0000017 | Anatoxin\_biosynthetic\_gene\_cluster | 40 | 37.1589085072 | 636 | 1e-181 |
| AEK75503.1 | type\_1\_polyketide\_synthase | BGC0000001 | Abyssomicin\_biosynthetic\_gene\_cluster | 40 | 40.569823435 | 636 | 1.7e-181 |
| ABC84470.1 | NIGAVIII | BGC0000114 | Nigericin\_biosynthetic\_gene\_cluster | 41 | 36.316211878 | 636 | 1.7e-181 |
| AFP87523.1 | type\_I\_polyketide\_synthase | BGC0001159 | Marinopyrrole\_biosynthetic\_gene\_cluster | 41 | 35.9951845907 | 636 | 1.7e-181 |
| AGI99482.1 | Type\_I\_polyketide\_synthase | BGC0001004 | Lobophorin\_biosynthetic\_gene\_cluster | 40 | 39.8475120385 | 635 | 2.2e-181 |
| AEZ54375.1 | PieA2 | BGC0000124 | Piericidin\_A1\_biosynthetic\_gene\_cluster | 37 | 44.823434992 | 635 | 2.2e-181 |
| AFU82617.1 | polyketide\_synthase | BGC0000998 | Guadinomine\_biosynthetic\_gene\_cluster | 42 | 36.0754414125 | 635 | 2.2e-181 |
| AGZ15474.1 | putative\_type\_I\_polyketide\_synthase | BGC0001036 | Polyoxypeptin\_biosynthetic\_gene\_cluster | 41 | 36.0754414125 | 635 | 2.9e-181 |
| ACB37740.1 | putative\_type\_I\_polyketide\_synthase | BGC0000162 | Tetrocarcin\_A\_biosynthetic\_gene\_cluster | 39 | 42.5762439807 | 634 | 6.5e-181 |
| AKD43765.1 | HerE | BGC0001349 | Heronamide\_biosynthetic\_gene\_cluster | 41 | 37.3595505618 | 634 | 6.5e-181 |
| ACR33079.1 | polyketide\_synthase | BGC0000017 | Anatoxin\_biosynthetic\_gene\_cluster | 40 | 37.0786516854 | 634 | 6.5e-181 |
| ADZ24998.1 | polyketide\_synthase | BGC0000380 | Leupyrrin\_biosynthetic\_gene\_cluster | 41 | 35.834670947 | 633 | 8.5e-181 |
| ABM21569.1 | CrpA | BGC0000975 | Cryptophycin\_biosynthetic\_gene\_cluster | 33 | 50.2808988764 | 633 | 8.5e-181 |
| AGZ15475.1 | putative\_type\_1\_modular\_polyketide\_synthase | BGC0001036 | Polyoxypeptin\_biosynthetic\_gene\_cluster | 40 | 36.3964686998 | 633 | 1.1e-180 |
| ACY06290.1 | type\_I\_polyketide\_synthase | BGC0001042 | Sanglifehrin\_A\_biosynthetic\_gene\_cluster | 38 | 40.5296950241 | 633 | 1.4e-180 |
| ctg1\_orf255 |  | BGC0001200 | Lorneic\_acid\_A\_biosynthetic\_gene\_cluster | 41 | 36.5168539326 | 633 | 1.4e-180 |
| BAB69198.1 | modular\_polyketide\_synthase | BGC0000117 | Oligomycin\_biosynthetic\_gene\_cluster | 40 | 38.7239165329 | 633 | 1.4e-180 |
| AAZ94390.1 | modular\_polyketide\_synthase | BGC0000040 | Concanamycin\_A\_biosynthetic\_gene\_cluster | 39 | 39.5264847512 | 632 | 1.9e-180 |
| AHB82053.1 | polyketide\_synthase | BGC0001019 | Microsclerodermins\_biosynthetic\_gene\_cluster | 40 | 36.3964686998 | 632 | 1.9e-180 |
| AAU04878.1 | polyketide\_synthase | BGC0000365 | Glycopeptidolipid\_biosynthetic\_gene\_cluster | 38 | 41.6131621188 | 632 | 1.9e-180 |
| AIW82279.1 | PuwB | BGC0001125 | Puwainaphycins\_biosynthetic\_gene\_cluster | 41 | 36.3563402889 | 631 | 4.2e-180 |
| BAC76492.1 | lankamycin\_synthase\_LkmAII | BGC0000085 | Lankamycin\_biosynthetic\_gene\_cluster | 39 | 38.5634028892 | 630 | 7.2e-180 |
| ADU86004.1 | putative\_modular\_polyketide\_synthase | BGC0000165 | Tiacumicin\_B\_biosynthetic\_gene\_cluster | 42 | 35.9149277689 | 630 | 7.2e-180 |
| CAQ34928.1 | polyketide\_synthase | BGC0000986 | Dkxanthene\_biosynthetic\_gene\_cluster | 40 | 36.0353130016 | 630 | 7.2e-180 |
| ABC84460.1 | NigAV | BGC0000114 | Nigericin\_biosynthetic\_gene\_cluster | 39 | 39.4060995185 | 630 | 7.2e-180 |
| AAO65806.1 | monensin\_polyketide\_synthase\_modules\_11\_and\_12 | BGC0000100 | Monensin\_biosynthetic\_gene\_cluster | 39 | 40.8507223114 | 630 | 9.4e-180 |
| AAF19810.1 | MtaB | BGC0001024 | Myxothiazol\_biosynthetic\_gene\_cluster | 39 | 38.8041733547 | 629 | 1.2e-179 |
| AAX98190.1 | polyketide\_synthase\_type\_I | BGC0000052 | ECO-02301\_biosynthetic\_gene\_cluster | 41 | 36.2359550562 | 629 | 1.2e-179 |
| AAM54077.1 | polyketide\_synthase | BGC0000020 | Ansamitocin\_biosynthetic\_gene\_cluster | 43 | 36.5168539326 | 629 | 1.6e-179 |
| CAD29793.1 | polyketide\_synthase\_type\_I | BGC0001015 | Microcystin\_biosynthetic\_gene\_cluster | 39 | 40.3290529695 | 629 | 1.6e-179 |
| CAQ34919.1 | polyketide\_synthase | BGC0000986 | Dkxanthene\_biosynthetic\_gene\_cluster | 40 | 35.9951845907 | 629 | 2.1e-179 |
| AAO62585.1 | peptide\_sythetase\_polyketide\_synthase\_fusion\_protein | BGC0001016 | Microcystin\_biosynthetic\_gene\_cluster | 31 | 62.3194221509 | 628 | 3.6e-179 |
| AAZ94391.1 | modular\_polyketide\_synthase | BGC0000040 | Concanamycin\_A\_biosynthetic\_gene\_cluster | 40 | 36.0353130016 | 628 | 4.7e-179 |
| CAQ64688.1 | lasalocid\_modular\_polyketide\_synthase | BGC0000087 | Lasalocid\_biosynthetic\_gene\_cluster | 41 | 35.9951845907 | 628 | 4.7e-179 |
| BAG85028.1 | putative\_polyketide\_synthase | BGC0000086 | Lasalocid\_biosynthetic\_gene\_cluster | 41 | 35.9951845907 | 628 | 4.7e-179 |
| AAO62584.1 | polyketide\_synthase\_type\_1 | BGC0001016 | Microcystin\_biosynthetic\_gene\_cluster | 41 | 36.797752809 | 628 | 4.7e-179 |
| ABX60153.1 | polyketide\_synthase | BGC0000978 | Cylindrospermopsin\_biosynthetic\_gene\_cluster | 40 | 36.0353130016 | 627 | 6.1e-179 |
| ADF88275.1 | polyketide\_synthase | BGC0000981 | Cylindrospermopsin\_biosynthetic\_gene\_cluster | 40 | 36.0754414125 | 627 | 7.9e-179 |
| ADC79618.1 | BafAIII | BGC0000028 | Bafilomycin\_biosynthetic\_gene\_cluster | 39 | 39.7271268058 | 627 | 7.9e-179 |
| AEE88289.1 | CurA | BGC0000976 | Curacin\_biosynthetic\_gene\_cluster | 37 | 40.4895666132 | 626 | 1e-178 |
| AAT70096.1 | CurA | BGC0001165 | Curacin\_biosynthetic\_gene\_cluster | 37 | 40.4895666132 | 626 | 1e-178 |
| AFV30250.1 | polyketide\_synthase | BGC0000075 | Hygrocin\_biosynthetic\_gene\_cluster | 40 | 36.3563402889 | 626 | 1.4e-178 |
| AAF86393.1 | FkbB | BGC0000994 | FK520\_biosynthetic\_gene\_cluster | 40 | 41.2921348315 | 626 | 1.4e-178 |
| AFL48532.1 | laidlomycin\_polyketide\_synthase\_(module\_11\_and\_module\_12) | BGC0000084 | Laidlomycin\_biosynthetic\_gene\_cluster | 38 | 40.3691813804 | 626 | 1.8e-178 |
| AEP40934.1 | polyketide\_synthase\_type\_I | BGC0000021 | Apoptolidin\_biosynthetic\_gene\_cluster | 40 | 36.0353130016 | 626 | 1.8e-178 |
| AAF19813.1 | MtaE | BGC0001024 | Myxothiazol\_biosynthetic\_gene\_cluster | 39 | 36.7174959872 | 625 | 2.3e-178 |
| CAG23957.2 | NRPS/PKS\_protein | BGC0001089 | Bacillaene\_biosynthetic\_gene\_cluster | 34 | 48.0738362761 | 625 | 3e-178 |
| CAN89633.1 | putative\_hybrid\_non-ribosomal\_peptide\_synthetase/polyketide\_synthase | BGC0001070 | Kirromycin\_biosynthetic\_gene\_cluster | 37 | 43.1380417335 | 625 | 3e-178 |
| ADF88280.1 | polyketide\_synthase | BGC0000981 | Cylindrospermopsin\_biosynthetic\_gene\_cluster | 39 | 36.9181380417 | 625 | 3e-178 |
| ABX60162.1 | polyketide\_synthase | BGC0000978 | Cylindrospermopsin\_biosynthetic\_gene\_cluster | 39 | 36.4365971108 | 624 | 3.9e-178 |
| ABY21539.1 | AngAII | BGC0000018 | Angolamycin\_biosynthetic\_gene\_cluster | 39 | 41.1717495987 | 624 | 3.9e-178 |
| ABF85931.1 | non-ribosomal\_peptide\_synthase/polyketide\_synthase\_Ta1 | BGC0001025 | Myxovirescin\_biosynthetic\_gene\_cluster | 33 | 49.7191011236 | 624 | 5.1e-178 |
| AFL48526.1 | laidlomycin\_polyketide\_synthase\_(module\_2) | BGC0000084 | Laidlomycin\_biosynthetic\_gene\_cluster | 41 | 36.316211878 | 624 | 6.7e-178 |
| CAQ64691.1 | lasalocid\_modular\_polyketide\_synthase | BGC0000087 | Lasalocid\_biosynthetic\_gene\_cluster | 41 | 36.3964686998 | 624 | 6.7e-178 |
| AGY62754.1 | EbeB | BGC0000051 | Ebelactone\_biosynthetic\_gene\_cluster | 41 | 36.3563402889 | 623 | 8.8e-178 |
| AEU17898.1 | putative\_type\_I\_PKS | BGC0001072 | Simocyclinone\_biosynthetic\_gene\_cluster | 40 | 37.3996789727 | 623 | 1.5e-177 |
| ADU86003.1 | putative\_modular\_polyketide\_synthase | BGC0000165 | Tiacumicin\_B\_biosynthetic\_gene\_cluster | 39 | 38.6035313002 | 622 | 2e-177 |
| CBD77736.1 | polyketide\_synthase | BGC0000974 | Crocacin\_biosynthetic\_gene\_cluster | 41 | 35.834670947 | 622 | 2e-177 |
| ADC79617.1 | BafAII | BGC0000028 | Bafilomycin\_biosynthetic\_gene\_cluster | 38 | 40.3691813804 | 622 | 2e-177 |
| CAO98849.1 | polyketide\_synthase\_AufF | BGC0000023 | Aurafuron\_biosynthetic\_gene\_cluster | 40 | 36.5168539326 | 622 | 2e-177 |
| CAQ34920.1 | polyketide\_synthase | BGC0000986 | Dkxanthene\_biosynthetic\_gene\_cluster | 40 | 35.8747993579 | 622 | 2.6e-177 |
| ABY21540.1 | AngAIII | BGC0000018 | Angolamycin\_biosynthetic\_gene\_cluster | 40 | 38.3226324238 | 622 | 2.6e-177 |
| ADB23403.1 | polyketide\_synthase\_type\_I | BGC0001062 | TLN-05220\_biosynthetic\_gene\_cluster | 38 | 41.0914927769 | 621 | 4.4e-177 |
| ABC84469.1 | NigAIX | BGC0000114 | Nigericin\_biosynthetic\_gene\_cluster | 40 | 36.2760834671 | 620 | 7.4e-177 |
| AEZ53949.1 | polyketide\_synthase | BGC0000144 | Salinomycin\_biosynthetic\_gene\_cluster | 37 | 41.6532905297 | 620 | 9.7e-177 |
| ADC79619.1 | BafAIV | BGC0000028 | Bafilomycin\_biosynthetic\_gene\_cluster | 39 | 41.4125200642 | 620 | 9.7e-177 |
| BAF85837.1 | modular\_polyketide\_synthase | BGC0000109 | Nemadectin\_biosynthetic\_gene\_cluster | 39 | 39.0449438202 | 619 | 1.3e-176 |
| BAG85031.1 | putative\_polyketide\_synthase | BGC0000086 | Lasalocid\_biosynthetic\_gene\_cluster | 41 | 36.6773675762 | 619 | 1.3e-176 |
| ANC94964.1 | AlmHIII | BGC0001396 | Aldgamycin\_biosynthetic\_gene\_cluster | 39 | 39.2857142857 | 619 | 2.2e-176 |
| AAY42396.1 | HctD | BGC0001000 | Hectochlorin\_biosynthetic\_gene\_cluster | 40 | 36.0353130016 | 618 | 2.8e-176 |
| AAQ82565.1 | FscB | BGC0000061 | FR-008\_biosynthetic\_gene\_cluster | 38 | 39.4060995185 | 618 | 3.7e-176 |
| CAQ18833.1 | polyketide\_synthase | BGC0000954 | Ajudazol\_biosynthetic\_gene\_cluster | 40 | 35.834670947 | 618 | 3.7e-176 |
| BAG85029.1 | putative\_polyketide\_synthase | BGC0000086 | Lasalocid\_biosynthetic\_gene\_cluster | 40 | 36.8378812199 | 618 | 4.8e-176 |
| CAD19091.1 | StiG\_protein | BGC0000153 | Stigmatellin\_biosynthetic\_gene\_cluster | 40 | 36.3964686998 | 618 | 4.8e-176 |
| CAQ64689.1 | lasalocid\_modular\_polyketide\_synthase | BGC0000087 | Lasalocid\_biosynthetic\_gene\_cluster | 40 | 36.8378812199 | 617 | 6.3e-176 |
| ABC84461.1 | NigAVI | BGC0000114 | Nigericin\_biosynthetic\_gene\_cluster | 41 | 36.9181380417 | 617 | 6.3e-176 |
| AAP42873.1 | NanA11 | BGC0000105 | Nanchangmycin\_biosynthetic\_gene\_cluster | 41 | 36.5971107544 | 617 | 8.2e-176 |
| AAT70106.1 | CurK | BGC0001165 | Curacin\_biosynthetic\_gene\_cluster | 38 | 37.8009630819 | 616 | 1.4e-175 |
| BI06\_RS39060 | polyketide\_synthase | BGC0001232 | Leucanicidin\_biosynthetic\_gene\_cluster | 40 | 38.1621187801 | 616 | 1.4e-175 |
| ADF88276.1 | polyketide\_synthase | BGC0000981 | Cylindrospermopsin\_biosynthetic\_gene\_cluster | 40 | 36.1556982343 | 616 | 1.4e-175 |
| AEE88279.1 | CurK | BGC0000976 | Curacin\_biosynthetic\_gene\_cluster | 38 | 37.8009630819 | 616 | 1.4e-175 |
| BI06\_RS39075 | polyketide\_synthase | BGC0001232 | Leucanicidin\_biosynthetic\_gene\_cluster | 39 | 40.2487961477 | 615 | 2.4e-175 |
| CAQ18835.1 | polyketide\_synthase | BGC0000954 | Ajudazol\_biosynthetic\_gene\_cluster | 40 | 35.9550561798 | 615 | 2.4e-175 |
| ABX60152.1 | polyketide\_synthase | BGC0000978 | Cylindrospermopsin\_biosynthetic\_gene\_cluster | 40 | 36.1556982343 | 614 | 7e-175 |
| ADA82585.1 | hybrid\_trans-AT\_polyketide\_synthase\_-\_nonribosomal\_peptide\_synthetase | BGC0001110 | Psymberin\_biosynthetic\_gene\_cluster | 34 | 46.8298555377 | 614 | 7e-175 |
| AAW03327.1 | CtaD | BGC0000982 | Cystothiazole\_A\_biosynthetic\_gene\_cluster | 37 | 43.0176565008 | 613 | 9.1e-175 |
| ADD82940.1 | Bat2 | BGC0001099 | Kalimantacin\_/\_batumin\_biosynthetic\_gene\_cluster | 35 | 47.2712680578 | 611 | 3.5e-174 |
| AEZ54377.1 | PieA4 | BGC0000124 | Piericidin\_A1\_biosynthetic\_gene\_cluster | 39 | 35.834670947 | 611 | 5.9e-174 |
| AAF19814.1 | MtaF | BGC0001024 | Myxothiazol\_biosynthetic\_gene\_cluster | 38 | 36.9983948636 | 611 | 5.9e-174 |
| AAK73513.1 | AmphB | BGC0000015 | Amphotericin\_biosynthetic\_gene\_cluster | 40 | 36.2359550562 | 611 | 5.9e-174 |
| ABB88523.1 | polyketide\_synthase\_type\_I | BGC0000050 | E-837\_biosynthetic\_gene\_cluster | 40 | 37.3996789727 | 610 | 7.7e-174 |
| AAZ94389.1 | modular\_polyketide\_synthase | BGC0000040 | Concanamycin\_A\_biosynthetic\_gene\_cluster | 40 | 36.1556982343 | 610 | 7.7e-174 |
| AHH34186.1 | polyketide\_synthase | BGC0001161 | 1-heptadecene\_biosynthetic\_gene\_cluster | 37 | 39.2857142857 | 610 | 7.7e-174 |
| AGZ15473.1 | putative\_type\_I\_polyketide\_synthase | BGC0001036 | Polyoxypeptin\_biosynthetic\_gene\_cluster | 39 | 37.2391653291 | 610 | 1e-173 |
| AAD03047.1 | type\_I\_polyketide\_synthase | BGC0000041 | Coronafacic\_acid\_biosynthetic\_gene\_cluster | 38 | 39.5666131621 | 610 | 1e-173 |
| AAO65797.1 | monensin\_polyketide\_synthase\_module\_2 | BGC0000100 | Monensin\_biosynthetic\_gene\_cluster | 40 | 35.9550561798 | 609 | 1.7e-173 |
| AAO65807.1 | monensin\_polyketide\_synthase\_module\_10 | BGC0000100 | Monensin\_biosynthetic\_gene\_cluster | 39 | 37.6404494382 | 609 | 2.2e-173 |
| BAE93728.1 | type\_I\_polyketide\_synthase | BGC0000164 | Tetronomycin\_biosynthetic\_gene\_cluster | 40 | 35.9149277689 | 609 | 2.2e-173 |
| AAS98777.1 | JamE | BGC0001001 | Jamaicamide\_biosynthetic\_gene\_cluster | 39 | 36.0754414125 | 608 | 2.9e-173 |
| ctg1\_orf9 |  | BGC0000053 | Elaiophylin\_biosynthetic\_gene\_cluster | 39 | 36.5168539326 | 608 | 3.8e-173 |
| AAW03329.1 | CtaF | BGC0000982 | Cystothiazole\_A\_biosynthetic\_gene\_cluster | 39 | 35.834670947 | 607 | 8.5e-173 |
| AGY62759.1 | EbeG | BGC0000051 | Ebelactone\_biosynthetic\_gene\_cluster | 41 | 36.7576243981 | 607 | 8.5e-173 |
| CAE45670.1 | borrelidin\_polyketide\_synthase,\_type\_I | BGC0000031 | Borrelidin\_biosynthetic\_gene\_cluster | 40 | 35.834670947 | 606 | 1.1e-172 |
| BAC76491.1 | lankamycin\_synthase\_LkmAIII | BGC0000085 | Lankamycin\_biosynthetic\_gene\_cluster | 38 | 40.0080256822 | 606 | 1.1e-172 |
| ABV83229.1 | CppB | BGC0000116 | Nystatin-like\_Pseudonocardia\_polyene\_biosynthetic\_gene\_cluster | 40 | 35.834670947 | 606 | 1.1e-172 |
| AAW03328.1 | CtaE | BGC0000982 | Cystothiazole\_A\_biosynthetic\_gene\_cluster | 39 | 36.7576243981 | 606 | 1.1e-172 |
| AAC68815.1 | FK506\_polyketide\_synthase | BGC0000353 | FK506\_biosynthetic\_gene\_cluster | 39 | 41.974317817 | 606 | 1.5e-172 |
| AAF71775.1 | NysB | BGC0000115 | Nystatin\_biosynthetic\_gene\_cluster | 40 | 36.1958266453 | 606 | 1.5e-172 |
| ABB52544.1 | putative\_type\_I\_polyketide\_synthase | BGC0000047 | Dihydrochalcomycin\_biosynthetic\_gene\_cluster | 39 | 38.0818619583 | 606 | 1.9e-172 |
| CCP20048.1 | divL1\_protein | BGC0001119 | Divergolide\_biosynthetic\_gene\_cluster | 38 | 38.2423756019 | 605 | 2.5e-172 |
| ABX60163.1 | polyketide\_synthase | BGC0000978 | Cylindrospermopsin\_biosynthetic\_gene\_cluster | 39 | 36.4767255217 | 605 | 2.5e-172 |
| ACY13415.1 | KR\_domain\_protein | BGC0001367 | Haliamide\_biosynthetic\_gene\_cluster | 36 | 45.7865168539 | 605 | 2.5e-172 |
| AGC45621.1 | polyketide\_synthase | BGC0001394 | Phenalamide\_biosynthetic\_gene\_cluster | 40 | 36.1155698234 | 605 | 2.5e-172 |
| AAK57189.1 | MxaE | BGC0001022 | Myxalamid\_biosynthetic\_gene\_cluster | 38 | 35.9550561798 | 605 | 3.2e-172 |
| ADZ24996.1 | polyketide\_synthase | BGC0000380 | Leupyrrin\_biosynthetic\_gene\_cluster | 39 | 36.797752809 | 605 | 3.2e-172 |
| ADF88277.1 | polyketide\_synthase | BGC0000981 | Cylindrospermopsin\_biosynthetic\_gene\_cluster | 39 | 36.4767255217 | 605 | 3.2e-172 |
| AFL48533.1 | laidlomycin\_polyketide\_synthase\_(module\_10) | BGC0000084 | Laidlomycin\_biosynthetic\_gene\_cluster | 39 | 35.9951845907 | 604 | 4.2e-172 |
| ctg1\_orf8 |  | BGC0001109 | Pederin\_biosynthetic\_gene\_cluster | 36 | 22.4317817014 | 300 | 1.7e-80 |
| CQR60494.1 | Polyketide\_synthase,\_type\_I,\_module\_8 | BGC0001287 | Chaxamycin\_A\_/\_chaxamycin\_B\_/\_chaxamycin\_C\_/\_chaxamycin\_D | 41 | 35.9149277689 | 604 | 5.5e-172 |
| CAD89777.1 | MelF\_protein | BGC0001010 | Melithiazol\_biosynthetic\_gene\_cluster | 38 | 36.3563402889 | 603 | 9.4e-172 |
| AEZ53948.1 | polyketide\_synthase | BGC0000144 | Salinomycin\_biosynthetic\_gene\_cluster | 39 | 36.5971107544 | 603 | 9.4e-172 |
| CAJ46689.1 | polyketide\_synthase | BGC0000969 | Chondramide\_biosynthetic\_gene\_cluster | 36 | 42.4157303371 | 602 | 1.6e-171 |
| AGC45622.1 | polyketide\_synthase | BGC0001394 | Phenalamide\_biosynthetic\_gene\_cluster | 38 | 35.8747993579 | 602 | 1.6e-171 |
| AMB48441.1 | polyketide\_synthase | BGC0001357 | Carbamidocyclophane\_biosynthetic\_gene\_cluster | 38 | 37.1990369181 | 602 | 2.1e-171 |
| ANC94963.1 | AlmHIV | BGC0001396 | Aldgamycin\_biosynthetic\_gene\_cluster | 39 | 38.1219903692 | 602 | 2.1e-171 |
| AEZ53951.1 | polyketide\_synthase | BGC0000144 | Salinomycin\_biosynthetic\_gene\_cluster | 39 | 35.9550561798 | 602 | 2.1e-171 |
| CAE45671.1 | borrelidin\_polyketide\_synthase,\_type\_I | BGC0000031 | Borrelidin\_biosynthetic\_gene\_cluster | 40 | 36.2760834671 | 601 | 3.6e-171 |
| ALD82523.1 | polyketide\_synthase | BGC0001212 | Nannocystin\_biosynthetic\_gene\_cluster | 40 | 37.8812199037 | 601 | 3.6e-171 |
| ABV97154.1 | Beta-ketoacyl\_synthase | BGC0000137 | Rifamycin\_biosynthetic\_gene\_cluster | 39 | 36.1556982343 | 601 | 3.6e-171 |
| AAS79462.1 | polyketide\_synthase\_subunit | BGC0000035 | Chalcomycin\_biosynthetic\_gene\_cluster | 39 | 38.202247191 | 601 | 6.1e-171 |
| CBD77732.1 | polyketide\_synthase | BGC0000974 | Crocacin\_biosynthetic\_gene\_cluster | 34 | 51.1235955056 | 600 | 8e-171 |
| ABO15860.1 | polyketide\_synthase | BGC0000130 | Pyrrolomycin\_biosynthetic\_gene\_cluster | 40 | 35.9550561798 | 599 | 2.3e-170 |
| CBD77738.1 | polyketide\_synthase | BGC0000974 | Crocacin\_biosynthetic\_gene\_cluster | 41 | 36.0353130016 | 599 | 2.3e-170 |
| AAS98781.1 | JamJ | BGC0001001 | Jamaicamide\_biosynthetic\_gene\_cluster | 37 | 36.9181380417 | 598 | 4e-170 |
| BAD08358.1 | polyketide\_synthase\_modules\_4 | BGC0000167 | Vicenistatin\_biosynthetic\_gene\_cluster | 40 | 37.4799357945 | 597 | 5.2e-170 |
| CAD19088.1 | StiD\_protein | BGC0000153 | Stigmatellin\_biosynthetic\_gene\_cluster | 38 | 36.8378812199 | 597 | 5.2e-170 |
| ABB05102.1 | LipPks1 | BGC0001003 | Lipomycin\_biosynthetic\_gene\_cluster | 37 | 41.8138041734 | 597 | 6.7e-170 |
| ABC84457.1 | NigAII | BGC0000114 | Nigericin\_biosynthetic\_gene\_cluster | 40 | 36.2359550562 | 596 | 1.5e-169 |
| AGC45624.1 | polyketide\_synthase | BGC0001394 | Phenalamide\_biosynthetic\_gene\_cluster | 38 | 36.6773675762 | 596 | 1.5e-169 |
| AIT55262.1 | GulE | BGC0000072 | Gulmirecin\_biosynthetic\_gene\_cluster | 37 | 42.3354735152 | 596 | 2e-169 |
| ADC45515.1 | modular\_polyketide\_synthase | BGC0000093 | Meilingmycin\_biosynthetic\_gene\_cluster | 41 | 35.8747993579 | 595 | 2.6e-169 |
| AHE80994.1 | PieA4 | BGC0001169 | Piericidin\_A1\_biosynthetic\_gene\_cluster | 40 | 36.0754414125 | 595 | 2.6e-169 |
| ACB46486.1 | polyketide\_synthase | BGC0000082 | Kijanimicin\_biosynthetic\_gene\_cluster | 40 | 36.1556982343 | 595 | 3.3e-169 |
| AAK57188.1 | MxaD | BGC0001022 | Myxalamid\_biosynthetic\_gene\_cluster | 39 | 35.9550561798 | 594 | 4.4e-169 |
| AEZ53953.1 | polyketide\_synthase | BGC0000144 | Salinomycin\_biosynthetic\_gene\_cluster | 37 | 38.0016051364 | 594 | 5.7e-169 |
| AAC01713.1 | RifD | BGC0000136 | Rifamycin\_biosynthetic\_gene\_cluster | 39 | 35.9951845907 | 594 | 7.5e-169 |
| CAO98850.1 | polyketide\_synthase\_AufG | BGC0000023 | Aurafuron\_biosynthetic\_gene\_cluster | 39 | 36.797752809 | 592 | 1.7e-168 |
| AEZ53950.1 | polyketide\_synthase | BGC0000144 | Salinomycin\_biosynthetic\_gene\_cluster | 38 | 37.5200642055 | 591 | 4.8e-168 |
| CAD89776.1 | MelE\_protein | BGC0001010 | Melithiazol\_biosynthetic\_gene\_cluster | 38 | 36.9582664526 | 591 | 6.3e-168 |
| AGY62753.1 | EbeA | BGC0000051 | Ebelactone\_biosynthetic\_gene\_cluster | 43 | 33.1460674157 | 590 | 1.1e-167 |
| CCE88381.1 | polyketide\_synthase | BGC0001034 | Pellasoren\_biosynthetic\_gene\_cluster | 40 | 35.7945425361 | 590 | 1.1e-167 |
| AAF00957.1 | McyG | BGC0001017 | Microcystin\_biosynthetic\_gene\_cluster | 32 | 51.5248796148 | 589 | 1.8e-167 |
| ABK32263.1 | AmbH | BGC0000014 | Ambruticin\_biosynthetic\_gene\_cluster | 40 | 35.7945425361 | 587 | 5.3e-167 |
| BAC57030.1 | protomycinolide\_IV\_synthase\_3 | BGC0000102 | Mycinamicin\_biosynthetic\_gene\_cluster | 35 | 47.5922953451 | 587 | 7e-167 |
| AFV96142.1 | polyketide\_synthase | BGC0001064 | Cylindrocyclophane\_biosynthetic\_gene\_cluster | 37 | 37.4799357945 | 587 | 7e-167 |
| AAF00958.1 | McyE | BGC0001017 | Microcystin\_biosynthetic\_gene\_cluster | 38 | 36.316211878 | 587 | 7e-167 |
| CCE88380.1 | polyketide\_synthase | BGC0001034 | Pellasoren\_biosynthetic\_gene\_cluster | 38 | 37.0385232745 | 587 | 9.1e-167 |
| CAD29795.1 | peptide\_synthetase | BGC0001015 | Microcystin\_biosynthetic\_gene\_cluster | 32 | 51.1637239165 | 586 | 2e-166 |
| AAM54078.1 | polyketide\_synthase | BGC0000020 | Ansamitocin\_biosynthetic\_gene\_cluster | 41 | 35.9550561798 | 586 | 2e-166 |
| AAO62582.1 | polyketide\_synthase\_peptide\_sythetase\_fusion\_protein | BGC0001016 | Microcystin\_biosynthetic\_gene\_cluster | 38 | 35.9149277689 | 585 | 2.7e-166 |
| BAG84248.1 | putative\_polyketide\_synthase | BGC0000257 | Prodigiosin\_biosynthetic\_gene\_cluster | 38 | 39.7672552167 | 585 | 3.5e-166 |
| ACB37742.1 | putative\_type\_I\_polyketide\_synthase | BGC0000162 | Tetrocarcin\_A\_biosynthetic\_gene\_cluster | 40 | 36.1556982343 | 585 | 3.5e-166 |
| AGI99495.1 | Type\_I\_polyketide\_synthase | BGC0001004 | Lobophorin\_biosynthetic\_gene\_cluster | 39 | 36.0353130016 | 584 | 5.9e-166 |
| AAP42867.1 | NanA7 | BGC0000105 | Nanchangmycin\_biosynthetic\_gene\_cluster | 39 | 36.1155698234 | 584 | 5.9e-166 |
| AAQ84144.1 | Plm4 | BGC0000123 | Phoslactomycin\_B\_biosynthetic\_gene\_cluster | 40 | 35.3130016051 | 583 | 1e-165 |
| AAQ84146.1 | Plm6 | BGC0000123 | Phoslactomycin\_B\_biosynthetic\_gene\_cluster | 39 | 35.834670947 | 582 | 1.7e-165 |
| BAQ25482.1 | type\_I\_polyketide\_synthase | BGC0001288 | Maklamicin\_biosynthetic\_gene\_cluster | 37 | 42.5762439807 | 582 | 2.2e-165 |
| WP\_063764078.1 | polyketide\_synthase | BGC0001348 | JBIR-100\_biosynthetic\_gene\_cluster | 38 | 37.1187800963 | 581 | 3.8e-165 |
| ABV97153.1 | Beta-ketoacyl\_synthase | BGC0000137 | Rifamycin\_biosynthetic\_gene\_cluster | 38 | 36.0353130016 | 581 | 5e-165 |
| CAD89773.1 | MelB\_protein | BGC0001009 | Melithiazol\_biosynthetic\_gene\_cluster | 37 | 36.2359550562 | 580 | 8.5e-165 |
| AAW03325.1 | CtaB | BGC0000982 | Cystothiazole\_A\_biosynthetic\_gene\_cluster | 37 | 35.9951845907 | 579 | 1.9e-164 |
| ABK32255.1 | AmbA | BGC0000014 | Ambruticin\_biosynthetic\_gene\_cluster | 38 | 35.9951845907 | 578 | 3.2e-164 |
| BAD38874.1 | polyketide\_synthase | BGC0000111 | Neocarzilin\_biosynthetic\_gene\_cluster | 40 | 36.9582664526 | 578 | 3.2e-164 |
| CQR60495.1 | Polyketide\_synthase,\_type\_I,\_module\_7 | BGC0001287 | Chaxamycin\_A\_/\_chaxamycin\_B\_/\_chaxamycin\_C\_/\_chaxamycin\_D | 40 | 36.0353130016 | 576 | 1.2e-163 |
| ABK32287.1 | JerA | BGC0000080 | Jerangolid\_biosynthetic\_gene\_cluster | 38 | 35.9951845907 | 576 | 1.6e-163 |
| AHB82051.1 | polyketide\_synthase | BGC0001019 | Microsclerodermins\_biosynthetic\_gene\_cluster | 36 | 42.1348314607 | 575 | 2.7e-163 |
| AHB82062.1 | polyketide\_synthase | BGC0001231 | Microsclerodermins\_biosynthetic\_gene\_cluster | 35 | 43.0176565008 | 575 | 2.7e-163 |
| AJO72742.1 | Type\_I\_modular\_polyketide\_synthase | BGC0001381 | Brasilinolide\_biosynthetic\_gene\_cluster | 40 | 34.3499197432 | 574 | 4.7e-163 |
| CAO98847.1 | polyketide\_synthase\_AufC | BGC0000023 | Aurafuron\_biosynthetic\_gene\_cluster | 38 | 36.1958266453 | 574 | 8e-163 |
| AAC69332.1 | type\_I\_polyketide\_synthase\_PikAIV | BGC0000094 | Methymycin\_/\_pikromycin\_biosynthetic\_gene\_cluster | 39 | 36.316211878 | 574 | 8e-163 |
| CCE88378.1 | polyketide\_synthase | BGC0001034 | Pellasoren\_biosynthetic\_gene\_cluster | 38 | 35.834670947 | 574 | 8e-163 |
| AAS98784.1 | JamM | BGC0001001 | Jamaicamide\_biosynthetic\_gene\_cluster | 38 | 35.9951845907 | 574 | 8e-163 |
| CCE88379.1 | polyketide\_synthase | BGC0001034 | Pellasoren\_biosynthetic\_gene\_cluster | 39 | 35.8747993579 | 572 | 2.3e-162 |
| CUI25672.1 | Thiolactomycin\_Hybrid\_PKS-NRPS\_system | BGC0001353 | Thiolactomycin\_biosynthetic\_gene\_cluster | 39 | 35.7544141252 | 572 | 2.3e-162 |
| AAP85336.1 | type\_I\_PKS | BGC0000233 | Hedamycin\_biosynthetic\_gene\_cluster | 39 | 36.7576243981 | 571 | 5.2e-162 |
| CAD29794.1 | peptide\_synthetase | BGC0001015 | Microcystin\_biosynthetic\_gene\_cluster | 37 | 36.6773675762 | 571 | 5.2e-162 |
| AJO72735.1 | Type\_I\_modular\_polyketide\_synthase | BGC0001381 | Brasilinolide\_biosynthetic\_gene\_cluster | 38 | 40.0080256822 | 571 | 6.8e-162 |
| CAQ18830.1 | polyketide\_synthase | BGC0000954 | Ajudazol\_biosynthetic\_gene\_cluster | 37 | 37.72070626 | 571 | 6.8e-162 |
| AFP87524.1 | type\_I\_polyketide\_synthase | BGC0001159 | Marinopyrrole\_biosynthetic\_gene\_cluster | 38 | 35.5939004815 | 571 | 6.8e-162 |
| CAQ43076.1 | polyketide\_synthase | BGC0000970 | Chondrochloren\_biosynthetic\_gene\_cluster | 38 | 36.3563402889 | 570 | 8.8e-162 |
| AAK73512.1 | AmphA | BGC0000015 | Amphotericin\_biosynthetic\_gene\_cluster | 39 | 37.9213483146 | 570 | 1.2e-161 |
| AAP42856.1 | NanA2 | BGC0000105 | Nanchangmycin\_biosynthetic\_gene\_cluster | 38 | 36.1958266453 | 570 | 1.2e-161 |
| AGC45619.1 | polyketide\_synthase | BGC0001394 | Phenalamide\_biosynthetic\_gene\_cluster | 37 | 36.1155698234 | 569 | 1.5e-161 |
| AEE88284.1 | CurF | BGC0000976 | Curacin\_biosynthetic\_gene\_cluster | 36 | 37.1589085072 | 568 | 3.4e-161 |
| AEE88283.1 | CurG | BGC0000976 | Curacin\_biosynthetic\_gene\_cluster | 37 | 36.4767255217 | 568 | 4.4e-161 |
| AAT70102.1 | CurG | BGC0001165 | Curacin\_biosynthetic\_gene\_cluster | 37 | 36.4767255217 | 568 | 4.4e-161 |
| AAF86392.1 | FkbC | BGC0000994 | FK520\_biosynthetic\_gene\_cluster | 36 | 40.9711075441 | 567 | 5.7e-161 |
| AHA12078.1 | polyketide\_synthase\_type\_1 | BGC0001172 | Chlorizidine\_A\_biosynthetic\_gene\_cluster | 38 | 36.316211878 | 567 | 5.7e-161 |
| AAT70101.1 | CurF | BGC0001165 | Curacin\_biosynthetic\_gene\_cluster | 36 | 37.1589085072 | 567 | 7.5e-161 |
| AHA38199.1 | GphF | BGC0000069 | Gephyronic\_acid\_biosynthetic\_gene\_cluster | 37 | 39.9678972713 | 566 | 1.7e-160 |
| AAC01712.2 | RifC | BGC0000136 | Rifamycin\_biosynthetic\_gene\_cluster | 39 | 36.1958266453 | 566 | 2.2e-160 |
| AGC45623.1 | polyketide\_synthase | BGC0001394 | Phenalamide\_biosynthetic\_gene\_cluster | 37 | 38.4028892456 | 564 | 4.8e-160 |
| CAJ46690.1 | polyketide\_synthase | BGC0000969 | Chondramide\_biosynthetic\_gene\_cluster | 39 | 35.8747993579 | 564 | 6.3e-160 |
| AHE80991.1 | PieA1 | BGC0001169 | Piericidin\_A1\_biosynthetic\_gene\_cluster | 37 | 40.3691813804 | 564 | 8.3e-160 |
| AAQ90174.1 | polyketide\_synthase\_type\_I | BGC0000128 | Pyoluteorin\_biosynthetic\_gene\_cluster | 38 | 36.1155698234 | 563 | 1.1e-159 |
| CAD19085.1 | StiA\_protein | BGC0000153 | Stigmatellin\_biosynthetic\_gene\_cluster | 35 | 41.2921348315 | 562 | 1.8e-159 |
| AAC38076.1 | polyketide\_synthase\_type\_I | BGC0000127 | Pyoluteorin\_biosynthetic\_gene\_cluster | 38 | 36.0754414125 | 562 | 1.8e-159 |
| AIT55263.1 | GulF | BGC0000072 | Gulmirecin\_biosynthetic\_gene\_cluster | 39 | 34.911717496 | 562 | 1.8e-159 |
| CBD77734.1 | polyketide\_synthase | BGC0000974 | Crocacin\_biosynthetic\_gene\_cluster | 38 | 37.4398073836 | 561 | 4.1e-159 |
| AIT55260.1 | GulC | BGC0000072 | Gulmirecin\_biosynthetic\_gene\_cluster | 37 | 35.8747993579 | 560 | 9.1e-159 |
| CAQ43079.1 | polyketide\_synthase | BGC0000970 | Chondrochloren\_biosynthetic\_gene\_cluster | 38 | 37.0385232745 | 560 | 9.1e-159 |
| CAD19089.1 | StiE\_protein | BGC0000153 | Stigmatellin\_biosynthetic\_gene\_cluster | 39 | 35.9550561798 | 559 | 2.7e-158 |
| CCP20050.1 | divL3\_protein | BGC0001119 | Divergolide\_biosynthetic\_gene\_cluster | 37 | 37.6805778491 | 558 | 3.5e-158 |
| ACR50791.1 | putative\_polyketide\_synthase | BGC0000163 | Tetronasin\_biosynthetic\_gene\_cluster | 36 | 36.1958266453 | 557 | 5.9e-158 |
| CAQ18838.1 | polyketide\_synthase | BGC0000954 | Ajudazol\_biosynthetic\_gene\_cluster | 37 | 40.088282504 | 557 | 1e-157 |
| BAF50727.1 | hybrid\_polyketide\_synthase-non\_ribosomal\_peptide\_synthetase | BGC0001116 | Virginiamycin\_biosynthetic\_gene\_cluster | 35 | 47.4719101124 | 556 | 1.7e-157 |
| ABO15861.1 | polyketide\_synthase | BGC0000130 | Pyrrolomycin\_biosynthetic\_gene\_cluster | 39 | 35.3531300161 | 555 | 2.9e-157 |
| CAD89775.1 | MelD\_protein | BGC0001010 | Melithiazol\_biosynthetic\_gene\_cluster | 37 | 37.8812199037 | 555 | 2.9e-157 |
| CAQ43075.1 | polyketide\_synthase | BGC0000970 | Chondrochloren\_biosynthetic\_gene\_cluster | 33 | 49.1573033708 | 551 | 4.2e-156 |
| AAF19812.1 | MtaD | BGC0001024 | Myxothiazol\_biosynthetic\_gene\_cluster | 38 | 36.7576243981 | 550 | 9.4e-156 |
| AAQ90173.1 | polyketide\_synthase\_type\_I | BGC0000128 | Pyoluteorin\_biosynthetic\_gene\_cluster | 38 | 36.4365971108 | 550 | 9.4e-156 |
| AAK57186.1 | MxaB2 | BGC0001022 | Myxalamid\_biosynthetic\_gene\_cluster | 37 | 36.797752809 | 549 | 1.6e-155 |
| CAG23960.2 | hybrid\_NRPS/PKS | BGC0001089 | Bacillaene\_biosynthetic\_gene\_cluster | 31 | 46.8699839486 | 549 | 2.1e-155 |
| ADH01485.1 | putative\_mixed\_polyketide\_synthase/non-ribosomal\_peptide\_synthetase | BGC0000995 | FR901464\_biosynthetic\_gene\_cluster | 34 | 46.7495987159 | 549 | 2.1e-155 |
| AIC32693.1 | FR9DEF | BGC0001113 | Spliceostatin\_/\_FR901464\_biosynthetic\_gene\_cluster | 34 | 46.7495987159 | 549 | 2.1e-155 |
| AGN11881.1 | TstDEF | BGC0001114 | Thailanstatin\_biosynthetic\_gene\_cluster | 35 | 46.6292134831 | 547 | 1e-154 |
| ALJ49921.1 | TtmH | BGC0001236 | Thiotetroamide\_biosynthetic\_gene\_cluster | 37 | 36.0353130016 | 541 | 4.4e-153 |
| AGS06887.1 | mixed\_type\_I\_polyketide\_synthase/non-ribosomal\_peptide\_synthetase,\_PedF-like\_protein | BGC0001092\_c2 | Diaphorin\_biosynthetic\_gene\_cluster | 31 | 47.3113964687 | 541 | 5.7e-153 |
| CUI25744.1 | Polyketide\_synthase/Nonribosomal\_peptide\_synthase | BGC0001354 | U-68204\_biosynthetic\_gene\_cluster | 38 | 36.0353130016 | 540 | 1.3e-152 |
| AIT55264.1 | GulA | BGC0000072 | Gulmirecin\_biosynthetic\_gene\_cluster | 37 | 38.2423756019 | 539 | 1.7e-152 |
| AIT55261.1 | GulD | BGC0000072 | Gulmirecin\_biosynthetic\_gene\_cluster | 38 | 36.9582664526 | 539 | 1.7e-152 |
| CAD15508.1 | probable\_polyketide\_synthase\_protein | BGC0001014 | Micacocidin\_biosynthetic\_gene\_cluster | 36 | 41.8940609952 | 538 | 4.9e-152 |
| CAQ43077.1 | polyketide\_synthase | BGC0000970 | Chondrochloren\_biosynthetic\_gene\_cluster | 37 | 36.9983948636 | 537 | 1.1e-151 |
| ACN64831.1 | PokM1 | BGC0001061 | Polyketomycin\_biosynthetic\_gene\_cluster | 36 | 36.2359550562 | 537 | 1.1e-151 |
| CUI25727.1 | Polyketide\_synthase/Nonribosomal\_peptide\_synthase | BGC0001355 | U-68204\_biosynthetic\_gene\_cluster | 37 | 35.834670947 | 536 | 1.8e-151 |
| CAD17792.1 | probable\_non\_ribosomal\_peptide\_synthetase\_protein | BGC0001363 | Ralsolamycin\_biosynthetic\_gene\_cluster | 33 | 49.7993579454 | 535 | 4.1e-151 |
| CUI25702.1 | Tu3010\_Hybrid\_PKS-NRPS\_system | BGC0001352 | Thiotetronate\_Tu\_3010\_biosynthetic\_gene\_cluster | 37 | 37.27929374 | 534 | 7e-151 |
| AAZ77673.1 | ChlB1 | BGC0000036 | Chlorothricin\_biosynthetic\_gene\_cluster | 36 | 36.2359550562 | 533 | 1.2e-150 |
| CAO98852.1 | polyketide\_synthase\_AufI | BGC0000023 | Aurafuron\_biosynthetic\_gene\_cluster | 35 | 37.9614767255 | 532 | 2.7e-150 |
| CAQ43078.1 | polyketide\_synthase | BGC0000970 | Chondrochloren\_biosynthetic\_gene\_cluster | 38 | 36.0353130016 | 527 | 8.6e-149 |
| AMK48247.1 | 6-methylsalicylic\_acid\_synthase | BGC0001351\_c2 | Cahuitamycins\_biosynthetic\_gene\_cluster | 36 | 36.0353130016 | 526 | 1.9e-148 |
| ABC36203.1 | thiotemplate\_mechanism\_natural\_product\_synthetase | BGC0001102 | Malleilactone\_biosynthetic\_gene\_cluster | 30 | 64.8073836276 | 525 | 3.3e-148 |
| AEH42474.1 | polyketide\_synthase | BGC0000032 | Calcimycin\_biosynthetic\_gene\_cluster | 39 | 33.7881219904 | 524 | 5.6e-148 |
| CCE88377.1 | non-ribosomal\_peptide\_synthetase/polyketide\_synthase | BGC0001034 | Pellasoren\_biosynthetic\_gene\_cluster | 36 | 39.165329053 | 524 | 9.5e-148 |
| CBW54671.1 | polyketide\_synthase/non\_ribosomal\_peptide\_synthetase | BGC0000971 | Cinnabaramide\_biosynthetic\_gene\_cluster | 36 | 37.27929374 | 523 | 1.6e-147 |
| ABY66019.1 | 6-methylsalicylic\_acid\_synthase | BGC0001008 | Maduropeptin\_biosynthetic\_gene\_cluster | 36 | 35.6741573034 | 520 | 1e-146 |
| AAK57190.1 | MxaF | BGC0001022 | Myxalamid\_biosynthetic\_gene\_cluster | 35 | 38.8041733547 | 520 | 1e-146 |
| ALJ49910.1 | TlmH | BGC0001237 | Thiolactomycin\_biosynthetic\_gene\_cluster | 36 | 36.1556982343 | 519 | 2.3e-146 |
| AIT55259.1 | GulB | BGC0000072 | Gulmirecin\_biosynthetic\_gene\_cluster | 38 | 33.3065810594 | 517 | 6.8e-146 |
| AAC38075.1 | polyketide\_synthase\_type\_I | BGC0000127 | Pyoluteorin\_biosynthetic\_gene\_cluster | 37 | 36.3964686998 | 515 | 2.6e-145 |
| AHA38200.1 | GphG | BGC0000069 | Gephyronic\_acid\_biosynthetic\_gene\_cluster | 39 | 32.7046548957 | 513 | 1.3e-144 |
| ABI91470.1 | beta-ketoacyl\_synthase | BGC0001094 | Enacyloxin\_biosynthetic\_gene\_cluster | 34 | 39.4462279294 | 512 | 3.7e-144 |
| BAQ21947.1 | putative\_type\_I\_polyketide\_synthase | BGC0001204 | Versipelostatin\_biosynthetic\_gene\_cluster | 45 | 25.6420545746 | 512 | 3.7e-144 |
| ADU85988.1 | putative\_iterative\_type\_I\_polyketide\_synthase | BGC0000165 | Tiacumicin\_B\_biosynthetic\_gene\_cluster | 35 | 36.2359550562 | 511 | 6.4e-144 |
| AGC24271.1 | PrlQ | BGC0001038 | Pyralomicin\_biosynthetic\_gene\_cluster | 36 | 36.2359550562 | 508 | 3.2e-143 |
| AAF26920.1 | polyketide\_synthase | BGC0000988 | Epothilone\_biosynthetic\_gene\_cluster | 37 | 36.2359550562 | 508 | 5.4e-143 |
| AAM70355.1 | CalO5 | BGC0000033 | Calicheamicin\_biosynthetic\_gene\_cluster | 36 | 36.1155698234 | 508 | 5.4e-143 |
| CAE02606.1 | polyketide\_synthase\_type\_I | BGC0000024 | Aureothin\_biosynthetic\_gene\_cluster | 40 | 30.7383627608 | 507 | 9.2e-143 |
| AHB82063.1 | polyketide\_synthase | BGC0001231 | Microsclerodermins\_biosynthetic\_gene\_cluster | 41 | 29.3739967897 | 507 | 1.2e-142 |
| WP\_042799407.1 | polyketide\_synthase | BGC0001283 | Arsenopolyketides\_biosynthetic\_gene\_cluster | 36 | 36.0754414125 | 506 | 1.6e-142 |
| CAF05651.1 | TubF\_protein | BGC0001053 | Tubulysin\_biosynthetic\_gene\_cluster | 34 | 48.0738362761 | 506 | 2e-142 |
| AHA38202.1 | GphI | BGC0000069 | Gephyronic\_acid\_biosynthetic\_gene\_cluster | 38 | 36.1556982343 | 505 | 3.5e-142 |
| ADB12490.1 | EpoC | BGC0000990 | Epothilone\_biosynthetic\_gene\_cluster | 36 | 36.2359550562 | 505 | 4.6e-142 |
| ABL86391.1 | hybrid\_polyketide\_synthase\_and\_nonribosomal\_peptide\_synthetase | BGC0000999 | Heat-stable\_antifungal\_factor\_biosynthetic\_gene\_cluster | 36 | 36.0754414125 | 504 | 5.9e-142 |
| AAF62882.1 | EpoC | BGC0000991 | Epothilone\_biosynthetic\_gene\_cluster | 36 | 36.2359550562 | 503 | 1.7e-141 |
| EYT83439.1 | beta-ketoacyl\_synthase | BGC0001213 | Nataxazole\_biosynthetic\_gene\_cluster | 36 | 35.0722311396 | 502 | 2.3e-141 |
| BAO98805.1 | putative\_polyketide\_synthase | BGC0001002 | Jawsamycin\_biosynthetic\_gene\_cluster | 37 | 33.3868378812 | 502 | 3.9e-141 |
| ABO15888.1 | polyketide\_synthase | BGC0000132 | Pyrrolomycin\_biosynthetic\_gene\_cluster | 39 | 30.4173354735 | 498 | 4.3e-140 |
| ACB46194.1 | polyketide\_synthase | BGC0000989 | Epothilone\_biosynthetic\_gene\_cluster | 36 | 36.2359550562 | 497 | 7.3e-140 |
| AGC09486.1 | LobS3 | BGC0001183 | Lobophorin\_biosynthetic\_gene\_cluster | 37 | 36.5569823435 | 497 | 9.5e-140 |
| BAG17643.1 | putative\_NRPS-type-I\_PKS\_fusion\_protein | BGC0001043 | SGR\_PTMs\_biosynthetic\_gene\_cluster | 35 | 36.3563402889 | 497 | 1.2e-139 |
| ACM79805.1 | ZmaA | BGC0001059 | Zwittermycin\_A\_biosynthetic\_gene\_cluster | 32 | 40.5296950241 | 494 | 6.2e-139 |
| CAQ34929.1 | putative\_polyketide\_synthase | BGC0000986 | Dkxanthene\_biosynthetic\_gene\_cluster | 35 | 35.9149277689 | 493 | 1.4e-138 |
| AGC65516.1 | TtbA | BGC0001050 | Thalassospiramide\_biosynthetic\_gene\_cluster | 34 | 46.5489566613 | 493 | 1.8e-138 |
| CAQ18832.1 | polyketide\_synthase | BGC0000954 | Ajudazol\_biosynthetic\_gene\_cluster | 35 | 36.1556982343 | 493 | 1.8e-138 |
| AEP40932.1 | polyketide\_synthase\_type\_I | BGC0000021 | Apoptolidin\_biosynthetic\_gene\_cluster | 40 | 29.5746388443 | 492 | 2.3e-138 |
| AHA38201.1 | GphH | BGC0000069 | Gephyronic\_acid\_biosynthetic\_gene\_cluster | 37 | 34.5906902087 | 492 | 3.1e-138 |
| EFL02193.1 | amino\_acid\_adenylation\_domain-containing\_protein | BGC0000996 | Frontalamides\_biosynthetic\_gene\_cluster | 34 | 39.2455858748 | 491 | 6.8e-138 |
| AGZ15472.1 | putative\_modular\_polyketide\_synthase | BGC0001036 | Polyoxypeptin\_biosynthetic\_gene\_cluster | 35 | 35.8747993579 | 490 | 8.9e-138 |
| ADH04680.1 | hybrid\_polyketide\_synthase/non-ribosomal\_peptide\_synthetase | BGC0001344 | Tubulysin\_biosynthetic\_gene\_cluster | 37 | 36.316211878 | 490 | 8.9e-138 |
| BAC76476.1 | multifunctional\_polyketide-peptide\_synthase\_LkcA | BGC0001100 | Lankacidin\_biosynthetic\_gene\_cluster | 32 | 47.191011236 | 490 | 8.9e-138 |
| EGJ35088.1 | polyketide\_synthase\_module | BGC0001163 | 1-heptadecene\_biosynthetic\_gene\_cluster | 31 | 49.9197431782 | 487 | 9.8e-137 |
| AAM77986.1 | iterative\_type\_I\_polyketide\_synthase | BGC0000112 | Neocarzinostatin\_biosynthetic\_gene\_cluster | 35 | 34.7913322632 | 487 | 1.3e-136 |
| CCM44338.1 | putative\_polyketide\_synthase | BGC0001056 | Zeamine\_biosynthetic\_gene\_cluster | 36 | 36.3563402889 | 486 | 1.7e-136 |
| AHB82052.1 | polyketide\_synthase | BGC0001019 | Microsclerodermins\_biosynthetic\_gene\_cluster | 40 | 27.5682182986 | 485 | 3.7e-136 |
| AAY89049.1 | polyketide\_synthase | BGC0001069 | Chivosazole\_biosynthetic\_gene\_cluster | 35 | 36.1155698234 | 485 | 3.7e-136 |
| AHB82065.1 | polyketide\_synthase | BGC0001231 | Microsclerodermins\_biosynthetic\_gene\_cluster | 40 | 28.3306581059 | 485 | 4.9e-136 |
| AEH42473.1 | polyketide\_synthase | BGC0000032 | Calcimycin\_biosynthetic\_gene\_cluster | 35 | 36.2359550562 | 484 | 6.4e-136 |
| ABP73645.1 | SalA | BGC0000145 | Salinosporamide\_A\_biosynthetic\_gene\_cluster | 35 | 38.0016051364 | 484 | 6.4e-136 |
| ACZ65476.1 | type\_I\_modular\_polyketide\_synthase | BGC0000140 | RK-682\_biosynthetic\_gene\_cluster | 37 | 34.3900481541 | 483 | 1.4e-135 |
| ABP53498.1 | beta-ketoacyl\_synthase | BGC0001041 | Salinosporamide\_biosynthetic\_gene\_cluster | 35 | 38.0016051364 | 483 | 1.4e-135 |
| ADH04682.1 | polyketide\_synthase | BGC0001344 | Tubulysin\_biosynthetic\_gene\_cluster | 36 | 36.0754414125 | 482 | 2.4e-135 |
| AHB82057.1 | polyketide\_synthase | BGC0001019 | Microsclerodermins\_biosynthetic\_gene\_cluster | 35 | 36.1958266453 | 480 | 1.2e-134 |
| AHB82070.1 | polyketide\_synthase | BGC0001231 | Microsclerodermins\_biosynthetic\_gene\_cluster | 36 | 36.4767255217 | 479 | 2e-134 |
| AAF15892.2 | NosB | BGC0001028 | Nostopeptolide\_biosynthetic\_gene\_cluster | 35 | 36.4767255217 | 479 | 2.7e-134 |
| AGY62756.1 | EbeD | BGC0000051 | Ebelactone\_biosynthetic\_gene\_cluster | 43 | 24.6789727127 | 478 | 3.5e-134 |
| AAS98787.1 | JamP | BGC0001001 | Jamaicamide\_biosynthetic\_gene\_cluster | 34 | 35.834670947 | 470 | 1.6e-131 |
| ADA69241.1 | cis-AT\_polyketide\_synthase | BGC0001071 | Nosperin\_biosynthetic\_gene\_cluster | 34 | 36.3964686998 | 469 | 2.1e-131 |
| AHE80993.1 | PieA3 | BGC0001169 | Piericidin\_A1\_biosynthetic\_gene\_cluster | 40 | 27.0866773676 | 468 | 4.7e-131 |
| AHB82054.1 | polyketide\_synthase | BGC0001019 | Microsclerodermins\_biosynthetic\_gene\_cluster | 39 | 28.5313001605 | 468 | 6.2e-131 |
| CAF05649.1 | TubD\_protein | BGC0001053 | Tubulysin\_biosynthetic\_gene\_cluster | 36 | 35.9951845907 | 468 | 6.2e-131 |
| AEZ54376.1 | PieA3 | BGC0000124 | Piericidin\_A1\_biosynthetic\_gene\_cluster | 41 | 27.0064205457 | 467 | 1.4e-130 |
| CAJ76291.1 | putative\_polyketide\_synthase | BGC0000972 | Colibactin\_biosynthetic\_gene\_cluster | 35 | 36.4767255217 | 465 | 5.2e-130 |
| ABX37384.1 | Beta-ketoacyl\_synthase | BGC0000984 | Delftibactin\_biosynthetic\_gene\_cluster | 35 | 36.797752809 | 464 | 6.8e-130 |
| AFV52200.1 | polyketide\_synthase\_module | BGC0000081 | Kedarcidin\_biosynthetic\_gene\_cluster | 35 | 37.6003210273 | 464 | 6.8e-130 |
| ABY83164.1 | Azi26 | BGC0000960 | Azinomycin\_B\_biosynthetic\_gene\_cluster | 33 | 35.4333868379 | 463 | 2e-129 |
| CCC55921.1 | non-ribosomal\_peptide\_synthetase/polyketide\_synthase\_hybrid\_protein | BGC0000973 | Collismycin\_A\_biosynthetic\_gene\_cluster | 35 | 36.5168539326 | 463 | 2e-129 |
| AEH42487.1 | polyketide\_synthase | BGC0000032 | Calcimycin\_biosynthetic\_gene\_cluster | 35 | 33.3868378812 | 462 | 2.6e-129 |
| ABL74938.1 | TlmVIII | BGC0001048 | Tallysomycin\_biosynthetic\_gene\_cluster | 35 | 36.5569823435 | 462 | 4.4e-129 |
| AAP42872.1 | NanA9 | BGC0000105 | Nanchangmycin\_biosynthetic\_gene\_cluster | 41 | 26.5650080257 | 461 | 7.5e-129 |
| AAS98200.1 | MSAS-type\_polyketide\_synthase | BGC0001273 | Asperlactone\_biosynthetic\_gene\_cluster | 32 | 35.6340288925 | 457 | 1.1e-127 |
| ctg12\_orf1 |  | BGC0000095 | Micromonolactam\_biosynthetic\_gene\_cluster | 42 | 25.0 | 449 | 2.3e-125 |
| AFD30954.1 | CrmA | BGC0000966 | Caerulomycin\_A\_biosynthetic\_gene\_cluster | 35 | 36.5569823435 | 445 | 4.3e-124 |
| AFL48521.1 | beta-ketosynthase | BGC0000084 | Laidlomycin\_biosynthetic\_gene\_cluster | 40 | 26.4446227929 | 445 | 5.6e-124 |
| CAO98848.1 | polyketide\_synthase\_AufE | BGC0000023 | Aurafuron\_biosynthetic\_gene\_cluster | 38 | 29.4943820225 | 444 | 7.3e-124 |
| AKA54627.1 | PKS | BGC0001216 | Splenocin\_biosynthetic\_gene\_cluster | 33 | 37.4398073836 | 442 | 2.8e-123 |
| AHD05619.1 | putative\_polyketide\_synthase\_subunit | BGC0001033 | Paenilamicin\_biosynthetic\_gene\_cluster | 33 | 35.9951845907 | 442 | 2.8e-123 |
| ABS90476.1 | NRPS | BGC0001106 | Oxazolomycin\_biosynthetic\_gene\_cluster | 43 | 24.2776886035 | 437 | 1.2e-121 |
| BAD38873.1 | polyketide\_synthase | BGC0000111 | Neocarzilin\_biosynthetic\_gene\_cluster | 34 | 36.0353130016 | 436 | 1.5e-121 |
| AHD05615.1 | putative\_non-ribosomal\_peptide\_ligase/\_polyketide\_synthase\_hybrid | BGC0001033 | Paenilamicin\_biosynthetic\_gene\_cluster | 33 | 36.1958266453 | 435 | 3.4e-121 |
| AAO65790.1 | ketosynthase-like\_protein | BGC0000100 | Monensin\_biosynthetic\_gene\_cluster | 38 | 27.9293739968 | 435 | 3.4e-121 |
| WP\_010639241.1 | beta-ketoacyl\_synthase | BGC0000958 | Antimycin\_biosynthetic\_gene\_cluster | 33 | 37.0385232745 | 435 | 4.4e-121 |
| AAG02357.1 | polyketide\_synthase | BGC0000963 | Bleomycin\_biosynthetic\_gene\_cluster | 34 | 38.1621187801 | 435 | 5.8e-121 |
| AIG62146.1 | 6-methylsalicylic\_acid\_synthase | BGC0000120 | Patulin\_biosynthetic\_gene\_cluster | 31 | 36.4365971108 | 434 | 9.9e-121 |
| EHA19289.1 | hypothetical\_protein | BGC0001124 | Pyranonigrin\_E\_biosynthetic\_gene\_cluster | 34 | 33.8683788122 | 431 | 6.4e-120 |
| ACD39774.1 | reducing\_polyketide\_synthase | BGC0000134 | Radicicol\_biosynthetic\_gene\_cluster | 32 | 37.4799357945 | 430 | 1.4e-119 |
| AHB38498.1 | EpxE | BGC0000346 | Epoxomicin\_biosynthetic\_gene\_cluster | 36 | 33.1460674157 | 430 | 1.4e-119 |
| AGC95324.1 | CurS1 | BGC0000045 | Dehydrocurvularin\_biosynthetic\_gene\_cluster | 32 | 38.202247191 | 429 | 2.4e-119 |
| ACR50795.1 | putative\_polyketide\_synthase | BGC0000163 | Tetronasin\_biosynthetic\_gene\_cluster | 37 | 30.3370786517 | 423 | 1.7e-117 |
| AFR69333.1 | polyketide\_synthase\_SpiC1 | BGC0001045 | Spiruchostatin\_biosynthetic\_gene\_cluster | 45 | 21.1075441413 | 423 | 1.7e-117 |
| AHB82058.1 | non\_ribosomal\_peptide\_synthetase | BGC0001019 | Microsclerodermins\_biosynthetic\_gene\_cluster | 41 | 24.5987158909 | 423 | 2.3e-117 |
| CBJ89764.1 | Polyketide\_synthase\_involved\_in\_xenocoumacin\_synthesis | BGC0001054 | Xenocoumacin\_biosynthetic\_gene\_cluster | 33 | 36.1556982343 | 422 | 3.9e-117 |
| CBJ89766.1 | Polyketide\_synthase\_involved\_in\_xenocoumacin\_synthesis | BGC0001054 | Xenocoumacin\_biosynthetic\_gene\_cluster | 32 | 37.3996789727 | 421 | 5.1e-117 |
| BAV32159.1 | polyketide\_synthase | BGC0001373 | Sordarin\_biosynthetic\_gene\_cluster | 32 | 34.3499197432 | 421 | 6.6e-117 |
| ABA02240.1 | polyketide\_synthase | BGC0000098 | Monacolin\_K\_biosynthetic\_gene\_cluster | 32 | 37.9213483146 | 418 | 4.3e-116 |
| BAK26562.1 | PKS-NRPS\_hybrid | BGC0000977 | Cyclopiazonic\_acid\_biosynthetic\_gene\_cluster | 33 | 37.6003210273 | 418 | 5.6e-116 |
| AHV78252.1 | ResS1 | BGC0001246 | Trans-resorcylide\_biosynthetic\_gene\_cluster | 33 | 35.2728731942 | 417 | 9.6e-116 |
| EAU32819.1 | 6-methylsalicylic\_acid\_synthase | BGC0000160 | Terreic\_acid\_biosynthetic\_gene\_cluster | 31 | 35.2327447833 | 416 | 2.1e-115 |
| BAA20102.2 | 6-methylsalicylic\_acid\_synthase | BGC0001276 | 6-methylsalicyclic\_acid\_biosynthetic\_gene\_cluster | 30 | 36.2760834671 | 416 | 2.1e-115 |
| ACZ57548.1 | polyketide\_synthase | BGC0000046 | Depudecin\_biosynthetic\_gene\_cluster | 31 | 36.1958266453 | 413 | 2.4e-114 |
| EHA28244.1 | hypothetical\_protein | BGC0001143 | Azanigerone\_biosynthetic\_gene\_cluster | 32 | 37.8812199037 | 412 | 4e-114 |
| ACD39758.1 | reducing\_polyketide\_synthase | BGC0000076 | Hypothemycin\_biosynthetic\_gene\_cluster | 31 | 36.6372391653 | 411 | 5.2e-114 |
| EAL89230.2 | LovB-like\_polyketide\_synthase,\_putative | BGC0000129 | Pyripyropene\_biosynthetic\_gene\_cluster | 32 | 37.27929374 | 411 | 6.9e-114 |
| ACD39767.1 | reducing\_polyketide\_synthase | BGC0000077 | Hypothemycin\_biosynthetic\_gene\_cluster | 31 | 36.6372391653 | 411 | 6.9e-114 |
| AAO23333.1 | NcpA | BGC0000397 | Nostocyclopeptide\_biosynthetic\_gene\_cluster | 40 | 23.9165329053 | 411 | 8.9e-114 |
| AHV78245.1 | LasS1 | BGC0001245 | Lasiodiplodin\_biosynthetic\_gene\_cluster | 32 | 35.7945425361 | 410 | 1.5e-113 |
| AFP73394.1 | FusA | BGC0001268 | Fusarin\_biosynthetic\_gene\_cluster | 34 | 34.3097913323 | 408 | 4.4e-113 |
| CBF90097.1 | hypothetical\_protein\_similar\_to\_polyketide\_synthase\_(Broad) | BGC0001271 | Emericellin\_biosynthetic\_gene\_cluster | 32 | 36.6773675762 | 408 | 4.4e-113 |
| AHB82071.1 | non\_ribosomal\_peptide\_synthetase | BGC0001231 | Microsclerodermins\_biosynthetic\_gene\_cluster | 41 | 23.9967897271 | 408 | 4.4e-113 |
| AGO59040.1 | PtaA | BGC0000121 | Pestheic\_acid\_biosynthetic\_gene\_cluster | 32 | 36.6773675762 | 406 | 1.7e-112 |
| AAN59953.1 | polyketide\_synthase\_1 | BGC0001258 | 1,8-dihydroxynaphthalene\_biosynthetic\_gene\_cluster | 31 | 36.797752809 | 406 | 1.7e-112 |
| EHA22196.1 | polyketide\_synthase | BGC0000170 | Yanuthone\_D\_biosynthetic\_gene\_cluster | 31 | 33.4269662921 | 406 | 1.7e-112 |
| ctg1\_orf0002 |  | BGC0001068 | Pyripyropene\_A\_biosynthetic\_gene\_cluster | 32 | 36.6773675762 | 406 | 1.7e-112 |
| CAJ87591.1 | putative\_peptide/polyketide\_synthetase | BGC0001055 | Yersiniabactin\_biosynthetic\_gene\_cluster | 34 | 36.1958266453 | 406 | 2.2e-112 |
| AAZ95017.1 | polyketide\_synthase | BGC0000048 | Dothistromin\_biosynthetic\_gene\_cluster | 29 | 43.4991974318 | 405 | 3.8e-112 |
| EAL89339.1 | polyketide\_synthase,\_putative | BGC0001403 | Trypacidin\_biosynthetic\_gene\_cluster | 31 | 38.1219903692 | 405 | 4.9e-112 |
| ABI91465.1 | beta-ketoacyl\_synthase | BGC0001094 | Enacyloxin\_biosynthetic\_gene\_cluster | 37 | 30.0561797753 | 404 | 8.4e-112 |
| ABC36687.1 | polyketide\_synthase | BGC0000964 | Burkholdac\_biosynthetic\_gene\_cluster | 43 | 20.7062600321 | 403 | 1.4e-111 |
| AAF17280.1 | NosC | BGC0001028 | Nostopeptolide\_biosynthetic\_gene\_cluster | 37 | 24.8796147673 | 403 | 2.4e-111 |
| AAV66110.2 | fusaridione\_A\_synthetase | BGC0000992 | Fusaridione\_A\_biosynthetic\_gene\_cluster | 33 | 34.5906902087 | 403 | 2.4e-111 |
| BAJ14522.1 | polyketide\_synthase | BGC0001253\_c4 | ACT-Toxin\_I\_biosynthetic\_gene\_cluster | 33 | 33.9085072231 | 402 | 3.2e-111 |
| ABP57747.1 | DepC | BGC0000993 | FK228\_biosynthetic\_gene\_cluster | 44 | 21.1476725522 | 402 | 4.2e-111 |
| AAB08104.3 | polyketide\_synthase | BGC0000155\_c3 | T-toxin\_biosynthetic\_gene\_cluster | 30 | 37.8009630819 | 402 | 4.2e-111 |
| AAP33839.2 | ochratoxin\_A\_polyketide\_synthase | BGC0001030 | Ochratoxin\_A\_biosynthetic\_gene\_cluster | 33 | 30.176565008 | 401 | 5.4e-111 |
| AEF33079.1 | polyketide\_synthase | BGC0001039 | Pyridomycin\_biosynthetic\_gene\_cluster | 32 | 36.9181380417 | 401 | 7.1e-111 |
| AAD34559.1 | polyketide\_synthase | BGC0000089 | Lovastatin\_biosynthetic\_gene\_cluster | 32 | 38.0818619583 | 401 | 7.1e-111 |
| AAT28740.1 | FUSS | BGC0000064 | Fusarin\_biosynthetic\_gene\_cluster | 33 | 34.0288924559 | 400 | 1.2e-110 |
| CAA73127.1 | HMWP1\_protein | BGC0000467 | Yersiniabactin\_biosynthetic\_gene\_cluster | 33 | 36.1958266453 | 399 | 2.1e-110 |
| EAW09117.1 | hybrid\_NRPS/PKS\_enzyme,\_putative | BGC0000983 | Cytochalasin\_biosynthetic\_gene\_cluster | 32 | 34.7110754414 | 399 | 2.1e-110 |
| BAE93739.1 | type\_I\_polyketide\_synthase-related\_protein | BGC0000164 | Tetronomycin\_biosynthetic\_gene\_cluster | 37 | 27.1268057785 | 398 | 4.6e-110 |
| BAB69698.1 | iturin\_A\_synthetase\_A | BGC0001098 | Iturin\_biosynthetic\_gene\_cluster | 32 | 33.8683788122 | 398 | 7.8e-110 |
| AAX35547.1 | polyketide\_syntase\_2 | BGC0001275 | 6-methylsalicyclic\_acid\_biosynthetic\_gene\_cluster | 30 | 35.2327447833 | 396 | 1.7e-109 |
| ANI24100.1 | nonribosomal\_peptide\_synthetase | BGC0001235 | Antalid\_biosynthetic\_gene\_cluster | 41 | 23.9967897271 | 396 | 3e-109 |
| EWG54266.1 | hypothetical\_protein | BGC0001190 | Fusaric\_acid\_biosynthetic\_gene\_cluster | 30 | 38.0016051364 | 396 | 3e-109 |
| AKL78824.1 | GLPKS3 | BGC0001187 | Xenolozoyenone\_biosynthetic\_gene\_cluster | 31 | 35.7945425361 | 396 | 3e-109 |
| ACS68554.1 | hybrid\_PKS-NRPS\_protein | BGC0001026 | NG-391\_biosynthetic\_gene\_cluster | 33 | 34.7110754414 | 395 | 3.9e-109 |
| CBF87072.1 | polyketide\_synthase,\_putative\_(Eurofung) | BGC0001290 | Emericellamide\_A\_/\_emericellamide\_B\_biosynthetic\_gene\_cluster | 31 | 37.4799357945 | 395 | 5.1e-109 |
| ABS74181.1 | BmyA | BGC0001090 | Bacillomycin\_biosynthetic\_gene\_cluster | 32 | 33.8683788122 | 394 | 6.6e-109 |
| CBF74114.1 | Conidial\_yellow\_pigment\_biosynthesis\_polyketide\_synthase\_(PKS)(EC\_2.3.1.-)\_[Source:UniProtKB/Swiss-Prot;Acc:Q03149] | BGC0000107 | Naphthopyrone\_biosynthetic\_gene\_cluster | 32 | 36.797752809 | 394 | 8.7e-109 |
| EAA65604.1 | hypothetical\_protein | BGC0000022 | Asperfuranone\_biosynthetic\_gene\_cluster | 29 | 40.3691813804 | 394 | 1.1e-108 |
| EAU38791.1 | hypothetical\_protein | BGC0000161 | Terrein\_biosynthetic\_gene\_cluster | 31 | 36.9181380417 | 394 | 1.1e-108 |
| CAB15186.3 | siderophore\_2,3-dihydroxybenzoate-glycine-threonine\_trimeric\_ester\_bacillibactin\_synthetase | BGC0000309 | Bacillibactin\_biosynthetic\_gene\_cluster | 41 | 23.1540930979 | 393 | 1.5e-108 |
| AGO86662.1 | equisetin\_synthetase | BGC0001255 | Equisetin\_biosynthetic\_gene\_cluster | 33 | 34.430176565 | 393 | 1.9e-108 |
| ABB90283.1 | polyketide\_synthase | BGC0001057 | Zearalenone\_biosynthetic\_gene\_cluster | 31 | 38.4028892456 | 392 | 4.3e-108 |
| ABA02239.1 | polyketide\_synthase | BGC0000098 | Monacolin\_K\_biosynthetic\_gene\_cluster | 31 | 37.8812199037 | 391 | 5.6e-108 |
| AAS89999.1 | PksA | BGC0000007 | Aflatoxin\_biosynthetic\_gene\_cluster | 31 | 36.7174959872 | 391 | 9.6e-108 |
| AHB38509.1 | EpnH | BGC0000345 | Eponemycin\_biosynthetic\_gene\_cluster | 33 | 34.911717496 | 390 | 1.3e-107 |
| ABD65958.1 | nonribosomal\_peptide\_synthetase | BGC0000341 | Enduracidin\_biosynthetic\_gene\_cluster | 41 | 23.6757624398 | 390 | 1.3e-107 |
| EED53479.1 | polyketide\_synthase,\_putative | BGC0001304 | Aflavarin\_biosynthetic\_gene\_cluster | 31 | 36.8378812199 | 390 | 1.6e-107 |
| ABU63483.1 | polyketide\_synthase | BGC0001272 | Elsinochrome\_B\_/\_elsinochrome\_C\_biosynthetic\_gene\_cluster | 30 | 39.1252006421 | 388 | 4.8e-107 |
| AAS92537.1 | PKS1 | BGC0001044 | Sirodesmin\_biosynthetic\_gene\_cluster | 30 | 36.797752809 | 388 | 4.8e-107 |
| AAS90093.1 | PksA | BGC0000006 | Aflatoxin\_biosynthetic\_gene\_cluster | 31 | 36.4365971108 | 388 | 4.8e-107 |
| AAS90022.1 | PksA | BGC0000008 | Aflatoxin\_biosynthetic\_gene\_cluster | 31 | 36.4365971108 | 388 | 6.2e-107 |
| CBD77749.1 | non-ribosomal\_peptide\_synthetase | BGC0000974 | Crocacin\_biosynthetic\_gene\_cluster | 41 | 23.6356340289 | 388 | 8.1e-107 |
| ABB90282.1 | polyketide\_synthase | BGC0001057 | Zearalenone\_biosynthetic\_gene\_cluster | 31 | 37.7608346709 | 388 | 8.1e-107 |
| BAE71314.1 | polyketide\_synthase | BGC0000004 | Aflatoxin\_biosynthetic\_gene\_cluster | 31 | 36.4365971108 | 388 | 8.1e-107 |
| CCA89328.1 | mixed\_trans-AT\_type\_I\_polyketide\_synthase/nonribosomal\_peptide\_synthetase | BGC0001111 | Rhizopodins\_biosynthetic\_gene\_cluster | 37 | 25.4414125201 | 386 | 2.4e-106 |
| CCE67070.1 | polyketide\_synthase | BGC0001242 | Fusarubin\_biosynthetic\_gene\_cluster | 30 | 38.1621187801 | 386 | 2.4e-106 |
| ACH72912.1 | AflC | BGC0000011 | Aflatoxin/sterigmatocystin\_biosynthetic\_gene\_cluster | 32 | 36.5971107544 | 386 | 2.4e-106 |
| AHF21228.1 | TriD | BGC0000449 | Tridecaptin\_biosynthetic\_gene\_cluster | 38 | 23.8764044944 | 386 | 3.1e-106 |
| AKQ22699.1 | malonyl\_CoA-acyl\_carrier\_protein\_transacylase | BGC0001186 | Misakinolide\_biosynthetic\_gene\_cluster | 35 | 27.5280898876 | 386 | 3.1e-106 |
| EAA59563.1 | hypothetical\_protein | BGC0000057 | F9775\_biosynthetic\_gene\_cluster | 31 | 37.5200642055 | 385 | 5.3e-106 |
| AAS90047.1 | PksA | BGC0000009 | Aflatoxin\_biosynthetic\_gene\_cluster | 30 | 36.4365971108 | 383 | 1.5e-105 |
| AAF08795.1 | MycA | BGC0001103 | Mycosubtilin\_biosynthetic\_gene\_cluster | 32 | 33.7881219904 | 383 | 2e-105 |
| AAC49191.1 | putative\_polyketide\_synthase | BGC0000152 | Sterigmatocystin\_biosynthetic\_gene\_cluster | 31 | 36.7174959872 | 383 | 2e-105 |
| CAQ34917.1 | polyketide\_synthase | BGC0000986 | Dkxanthene\_biosynthetic\_gene\_cluster | 37 | 26.9261637239 | 383 | 2e-105 |
| ADN43685.1 | DmbS | BGC0001136 | Desmethylbassianin\_biosynthetic\_gene\_cluster | 32 | 35.9951845907 | 380 | 1.3e-104 |
| AGE25480.1 | amino\_acid\_adenylation\_protein | BGC0001208\_c2 | Poaeamide\_biosynthetic\_gene\_cluster | 38 | 26.0032102729 | 380 | 1.7e-104 |
| AAZ55900.1 | non-ribosomal\_peptide\_synthase:Amino\_acid\_adenylation | BGC0000359 | Fuscachelin\_biosynthetic\_gene\_cluster | 40 | 24.077046549 | 379 | 2.2e-104 |
| BAC20566.1 | polyketide\_synthase | BGC0000039 | Compactin\_biosynthetic\_gene\_cluster | 30 | 37.2391653291 | 379 | 2.9e-104 |
| AKA59448.1 | polyketide\_synthase | BGC0001203 | Clarexpoxcin\_biosynthetic\_gene\_cluster | 34 | 32.8651685393 | 379 | 3.8e-104 |
| AKC54422.1 | fumosorinone\_biosynthesis\_polyketide\_synthase | BGC0001218 | Fumosorinone\_biosynthetic\_gene\_cluster | 32 | 35.4333868379 | 378 | 4.9e-104 |
| AHZ20774.1 | non-ribosomal\_peptide\_synthase | BGC0000369 | Hassallidins\_biosynthetic\_gene\_cluster | 38 | 23.6356340289 | 378 | 4.9e-104 |
| AFN27480.1 | pks\_BonA | BGC0000173 | Bongkrekic\_acid\_biosynthetic\_gene\_cluster | 41 | 21.5088282504 | 378 | 6.4e-104 |
| CAL69597.1 | nonribosomal\_peptide\_synthase | BGC0001049 | Tenellin\_biosynthetic\_gene\_cluster | 32 | 35.7544141252 | 377 | 1.1e-103 |
| AHZ34240.1 | CipC | BGC0001389 | Cichopeptin\_biosynthetic\_gene\_cluster | 40 | 23.3948635634 | 377 | 1.4e-103 |
| BAH43766.1 | tyrocidine\_synthetase\_III | BGC0000452 | Tyrocidine\_biosynthetic\_gene\_cluster | 37 | 24.8394863563 | 377 | 1.4e-103 |
| AEG64697.1 | LpmC | BGC0000379 | Laspartomycin\_biosynthetic\_gene\_cluster | 40 | 23.3547351525 | 377 | 1.4e-103 |
| KPN93065.1 | NunD | BGC0001416 | Nunapeptin\_/\_nunamycin\_biosynthetic\_gene\_cluster | 40 | 23.2744783307 | 376 | 2.4e-103 |
| AEI70245.1 | nonribosomal\_peptide\_synthetase\_NRPS | BGC0000401 | Paenibactin\_biosynthetic\_gene\_cluster | 41 | 23.4751203852 | 374 | 7.1e-103 |
| ANQ43349.1 | non-ribosomal\_peptide\_synthetase | BGC0001347\_c2 | Poaeamide\_B\_biosynthetic\_gene\_cluster | 38 | 26.0032102729 | 373 | 2.1e-102 |
| AHV78253.1 | ResS2 | BGC0001246 | Trans-resorcylide\_biosynthetic\_gene\_cluster | 30 | 39.0048154093 | 372 | 3.5e-102 |
| AEA30272.1 | peptide\_synthetase | BGC0000429 | Skyllamycin\_biosynthetic\_gene\_cluster | 38 | 25.3611556982 | 372 | 4.6e-102 |
| AKA59437.1 | polyketide\_synthase | BGC0001202 | Landepoxcin\_biosynthetic\_gene\_cluster | 33 | 33.1460674157 | 371 | 1e-101 |
| ADI59533.1 | CorK | BGC0001091 | Corallopyronin\_biosynthetic\_gene\_cluster | 36 | 26.9261637239 | 370 | 1.3e-101 |
| ACD39753.1 | non-reducing\_polyketide\_synthase | BGC0000076 | Hypothemycin\_biosynthetic\_gene\_cluster | 30 | 36.5168539326 | 369 | 3e-101 |
| ACD39770.1 | non-reducing\_polyketide\_synthase | BGC0000134 | Radicicol\_biosynthetic\_gene\_cluster | 30 | 36.5168539326 | 368 | 5.1e-101 |
| AAN85523.1 | polyketide\_synthase | BGC0001101 | Leinamycin\_biosynthetic\_gene\_cluster | 39 | 22.7126805778 | 368 | 6.7e-101 |
| ACD39762.1 | non-reducing\_polyketide\_synthase | BGC0000077 | Hypothemycin\_biosynthetic\_gene\_cluster | 30 | 36.5168539326 | 368 | 6.7e-101 |
| ABB76806.1 | polyketide\_synthase\_2 | BGC0000155\_c2 | T-toxin\_biosynthetic\_gene\_cluster | 30 | 33.948635634 | 366 | 2.5e-100 |
| CBJ89760.1 | Polyketide\_synthase\_involved\_in\_xenocoumacin\_synthesis | BGC0001054 | Xenocoumacin\_biosynthetic\_gene\_cluster | 32 | 33.4269662921 | 366 | 3.3e-100 |
| AHV78247.1 | LasS2 | BGC0001245 | Lasiodiplodin\_biosynthetic\_gene\_cluster | 31 | 36.2359550562 | 365 | 5.6e-100 |
| EAQ86385.1 | hypothetical\_protein | BGC0001405 | Chaetoviridin\_/\_chaetomugilin\_biosynthetic\_gene\_cluster | 32 | 33.1059390048 | 364 | 7.4e-100 |
| ABC34108.1 | JamP | BGC0000961 | Bactobolin\_biosynthetic\_gene\_cluster | 33 | 33.1861958266 | 364 | 7.4e-100 |
| ABI91467.1 | beta-ketoacyl\_synthase | BGC0001094 | Enacyloxin\_biosynthetic\_gene\_cluster | 38 | 22.632423756 | 364 | 9.6e-100 |
| WP\_028678149.1 | non-ribosomal\_peptide\_synthetase | BGC0001228 | Retimycin\_biosynthetic\_gene\_cluster | 40 | 23.0738362761 | 364 | 1.3e-99 |
| AJF34464.1 | Txo2 | BGC0001207 | Teixobactin\_biosynthetic\_gene\_cluster | 39 | 24.4783306581 | 364 | 1.3e-99 |
| KPN93064.1 | NupB | BGC0001416 | Nunapeptin\_/\_nunamycin\_biosynthetic\_gene\_cluster | 38 | 26.4847512039 | 364 | 1.3e-99 |
| ABH06369.2 | MassC | BGC0000389 | Massetolide\_A\_biosynthetic\_gene\_cluster | 36 | 26.3242375602 | 363 | 1.6e-99 |
| ABA23460.1 | Amino\_acid\_adenylation | BGC0000427 | Shinorine\_biosynthetic\_gene\_cluster | 37 | 24.5585874799 | 363 | 1.6e-99 |
| CCT67991.1 | bikaverin\_cluster-polyketide\_synthase | BGC0000030 | Bikaverin\_biosynthetic\_gene\_cluster | 29 | 37.1589085072 | 363 | 2.8e-99 |
| ALD82521.1 | polyketide\_synthase | BGC0001212 | Nannocystin\_biosynthetic\_gene\_cluster | 43 | 20.304975923 | 363 | 2.8e-99 |
| CCH26295.1 | putative\_polyketide\_synthase | BGC0001158\_c6 | Bikaverin\_biosynthetic\_gene\_cluster | 29 | 36.9983948636 | 363 | 2.8e-99 |
| ADN68476.1 | SorA | BGC0000184 | Sorangicin\_biosynthetic\_gene\_cluster | 38 | 21.9903691814 | 362 | 3.6e-99 |
| AHD05679.1 | putative\_non-ribosomal\_peptide\_ligase/\_polyketide\_synthase\_hybrid | BGC0000402 | Paenilarvins\_biosynthetic\_gene\_cluster | 37 | 24.3980738363 | 362 | 3.6e-99 |
| BAD97694.1 | Aft9-1 | BGC0000003 | AF-toxin\_biosynthetic\_gene\_cluster | 31 | 33.6276083467 | 362 | 4.8e-99 |
| BAV19379.1 | polyketide\_synthase | BGC0001390 | LL-Z1272beta\_biosynthetic\_gene\_cluster | 31 | 34.3900481541 | 362 | 4.8e-99 |
| CAY48788.1 | putative\_non-ribosomal\_peptide\_synthetase | BGC0001312 | Viscosin\_biosynthetic\_gene\_cluster | 40 | 22.9133226324 | 361 | 6.2e-99 |
| BAH04162.1 | trsJ | BGC0000450 | Triostin\_A\_biosynthetic\_gene\_cluster | 40 | 22.9935794543 | 361 | 8.1e-99 |
| ALA62323.1 | methylphloroacetophenone\_synthase | BGC0001309 | Usnic\_acid\_biosynthetic\_gene\_cluster | 29 | 39.2455858748 | 360 | 1.8e-98 |
| ABX37383.1 | amino\_acid\_adenylation\_domain\_protein | BGC0000984 | Delftibactin\_biosynthetic\_gene\_cluster | 40 | 23.2343499197 | 360 | 1.8e-98 |
| AEO14744.1 | NdaB | BGC0000396 | Nodularin\_biosynthetic\_gene\_cluster | 36 | 23.6757624398 | 359 | 2.4e-98 |
| ABI91469.1 | beta-ketoacyl\_synthase | BGC0001094 | Enacyloxin\_biosynthetic\_gene\_cluster | 38 | 23.3547351525 | 359 | 3.1e-98 |
| AEA30274.1 | peptide\_synthetase | BGC0000429 | Skyllamycin\_biosynthetic\_gene\_cluster | 40 | 23.113964687 | 359 | 4e-98 |
| AHI59109.1 | locillomycin\_synthase\_B | BGC0001005 | Locillomycin\_biosynthetic\_gene\_cluster | 36 | 24.3178170144 | 358 | 5.3e-98 |
| AHD05678.1 | nonribosomal\_peptide\_ligase\_subunit | BGC0000402 | Paenilarvins\_biosynthetic\_gene\_cluster | 37 | 24.077046549 | 358 | 6.9e-98 |
| KPN90376.1 | NupC | BGC0001416 | Nunapeptin\_/\_nunamycin\_biosynthetic\_gene\_cluster | 37 | 26.5248796148 | 357 | 1.5e-97 |
| AAO23334.1 | NcpB | BGC0000397 | Nostocyclopeptide\_biosynthetic\_gene\_cluster | 36 | 24.7592295345 | 356 | 2.6e-97 |
| AEF33078.1 | dimodular\_nonribosomal\_peptide\_synthetase | BGC0001039 | Pyridomycin\_biosynthetic\_gene\_cluster | 40 | 23.5152487961 | 356 | 2.6e-97 |
| AJF34463.1 | Txo1 | BGC0001207 | Teixobactin\_biosynthetic\_gene\_cluster | 38 | 24.3178170144 | 356 | 2.6e-97 |
| ABW17376.1 | PsoB | BGC0000411 | Putisolvin\_biosynthetic\_gene\_cluster | 37 | 26.2439807384 | 356 | 3.4e-97 |
| AHZ34241.1 | CipD | BGC0001389 | Cichopeptin\_biosynthetic\_gene\_cluster | 37 | 24.3178170144 | 356 | 3.4e-97 |
| AAY32964.1 | DszA | BGC0001093 | Disorazole\_biosynthetic\_gene\_cluster | 35 | 26.4847512039 | 355 | 4.5e-97 |
| CAJ96468.1 | Non-ribosomal\_peptide\_synthetase\_modules\_and\_related\_proteins | BGC0000330 | Cupriachelin\_biosynthetic\_gene\_cluster | 38 | 23.5152487961 | 355 | 5.8e-97 |
| EFE73313.1 | nonribosomal\_peptide\_synthetase | BGC0000431 | Stenothricin\_biosynthetic\_gene\_cluster | 38 | 26.2439807384 | 355 | 5.8e-97 |
| AAK57184.1 | MxaA | BGC0001022 | Myxalamid\_biosynthetic\_gene\_cluster | 37 | 24.8394863563 | 355 | 5.8e-97 |
| ERM18799.1 | polyketide\_synthase | BGC0000172 | Basiliskamides\_biosynthetic\_gene\_cluster | 36 | 24.2375601926 | 354 | 7.6e-97 |
| ABC35522.1 | thiotemplate\_mechanism\_natural\_product\_synthetase | BGC0000186 | Thailandamide\_biosynthetic\_gene\_cluster | 37 | 24.2375601926 | 354 | 7.6e-97 |
| AET98906.1 | putative\_non-ribosomal\_peptide\_synthetase | BGC0000415 | Quinomycin\_biosynthetic\_gene\_cluster | 39 | 22.9935794543 | 354 | 9.9e-97 |
| ABH06368.2 | MassB | BGC0000389 | Massetolide\_A\_biosynthetic\_gene\_cluster | 40 | 23.9566613162 | 354 | 1.3e-96 |
| AGE25479.1 | putative\_non-ribosomal\_peptide\_synthetase | BGC0001208\_c2 | Poaeamide\_biosynthetic\_gene\_cluster | 38 | 23.7961476726 | 352 | 2.9e-96 |
| AFH75320.1 | nonribosomal\_peptide\_synthetase | BGC0000425 | Sessilin\_biosynthetic\_gene\_cluster | 36 | 26.2439807384 | 352 | 2.9e-96 |
| CAK15814.1 | putative\_non-ribosomal\_peptide\_synthetase,\_terminal\_component | BGC0000344 | Entolysin\_biosynthetic\_gene\_cluster | 37 | 26.0433386838 | 352 | 2.9e-96 |
| BAH43764.1 | tyrocidine\_synthetase\_I | BGC0000452 | Tyrocidine\_biosynthetic\_gene\_cluster | 35 | 23.7158908507 | 352 | 2.9e-96 |
| AEH59100.1 | amino\_acid\_adenylation\_domain-containing\_protein/NRPS | BGC0000385 | Lysobactin\_biosynthetic\_gene\_cluster | 39 | 23.9165329053 | 352 | 4.9e-96 |
| ADY00130.1 | polyketide\_synthase | BGC0000104 | Mycophenolic\_acid\_biosynthetic\_gene\_cluster | 29 | 39.2857142857 | 351 | 6.4e-96 |
| AFH75330.1 | nonribosomal\_peptide\_synthetase | BGC0000398 | Orfamide\_biosynthetic\_gene\_cluster | 37 | 26.6853932584 | 351 | 8.4e-96 |
| AGM14933.1 | xantholysin\_synthetase\_B | BGC0000463 | Xantholysin\_biosynthetic\_gene\_cluster | 36 | 26.0032102729 | 351 | 1.1e-95 |
| AFJ14794.1 | PlpE | BGC0000403 | Pelgipeptin\_biosynthetic\_gene\_cluster | 35 | 25.1203852327 | 351 | 1.1e-95 |
| ALD83687.1 | tAT\_polyketide\_synthase | BGC0001300 | Anthracimycin\_biosynthetic\_gene\_cluster | 35 | 24.1573033708 | 350 | 1.4e-95 |
| AHZ34243.1 | CipF | BGC0001389 | Cichopeptin\_biosynthetic\_gene\_cluster | 37 | 26.3643659711 | 350 | 1.4e-95 |
| ALD83702.1 | tAT\_polyketide\_synthase | BGC0001299 | Chlorotonil\_biosynthetic\_gene\_cluster | 36 | 26.6853932584 | 350 | 1.9e-95 |
| ANQ43348.1 | non-ribosomal\_peptide\_synthetase | BGC0001347\_c2 | Poaeamide\_B\_biosynthetic\_gene\_cluster | 39 | 23.7158908507 | 350 | 1.9e-95 |
| AAY37655.1 | Amino\_acid\_adenylation | BGC0000437 | Syringomycin\_biosynthetic\_gene\_cluster | 38 | 25.3611556982 | 350 | 1.9e-95 |
| AHH53506.1 | non-ribosomal\_peptide\_synthetase | BGC0000439 | Taromycin\_biosynthetic\_gene\_cluster | 38 | 24.2776886035 | 350 | 1.9e-95 |
| CAM56770.1 |  | BGC0000354 | Friulimicin\_biosynthetic\_gene\_cluster | 38 | 24.3579454254 | 349 | 2.4e-95 |
| CAN89632.1 | putative\_polyketide\_synthase | BGC0001070 | Kirromycin\_biosynthetic\_gene\_cluster | 41 | 21.4285714286 | 349 | 3.2e-95 |
| ALD83688.1 | tAT\_polyketide\_synthase | BGC0001300 | Anthracimycin\_biosynthetic\_gene\_cluster | 38 | 21.3081861958 | 349 | 4.2e-95 |
| AAM12913.2 | MmpD | BGC0000182 | Mupirocin\_biosynthetic\_gene\_cluster | 37 | 21.6693418941 | 349 | 4.2e-95 |
| AAO62587.1 | peptide\_sythetase | BGC0001016 | Microcystin\_biosynthetic\_gene\_cluster | 36 | 24.5585874799 | 348 | 5.5e-95 |
| ALV82356.1 | CDA\_peptide\_synthetase\_I | BGC0001370 | Lipopeptide\_8D1-1\_/\_lipopeptide\_8D1-2\_biosynthetic\_gene\_cluster | 39 | 24.518459069 | 348 | 5.5e-95 |
| AAX31557.1 | peptide\_synthetase\_1 | BGC0000336 | Daptomycin\_biosynthetic\_gene\_cluster | 39 | 23.4751203852 | 348 | 7.1e-95 |
| CTQ34882.1 | AtcE;\_polyketide\_synthase,\_modules\_5-7 | BGC0001301 | Anthracimycin\_biosynthetic\_gene\_cluster | 34 | 28.7319422151 | 348 | 7.1e-95 |
| ACY01391.1 | AT-less\_polyketide\_synthase | BGC0000177 | Dorrigocin\_/\_migrastatin\_biosynthetic\_gene\_cluster | 36 | 26.0032102729 | 348 | 7.1e-95 |
| ABX37382.1 | amino\_acid\_adenylation\_domain\_protein | BGC0000984 | Delftibactin\_biosynthetic\_gene\_cluster | 36 | 26.0834670947 | 348 | 7.1e-95 |
| ABF89568.1 | polyketide\_synthase | BGC0001025 | Myxovirescin\_biosynthetic\_gene\_cluster | 36 | 25.0401284109 | 348 | 7.1e-95 |
| AAO72425.1 | syringopeptin\_synthetase\_C | BGC0000438 | Syringopeptin\_biosynthetic\_gene\_cluster | 38 | 25.3611556982 | 347 | 9.3e-95 |
| CCA89329.1 | trans-AT\_type\_I\_polyketide\_synthase | BGC0001111 | Rhizopodins\_biosynthetic\_gene\_cluster | 37 | 23.2343499197 | 347 | 1.2e-94 |
| BAD38875.1 | polyketide\_synthase | BGC0000111 | Neocarzilin\_biosynthetic\_gene\_cluster | 37 | 23.1942215088 | 347 | 1.2e-94 |
| CDM36726.1 | Beta-ketoacyl\_synthase | BGC0001360 | Mycophenolic\_acid\_biosynthetic\_gene\_cluster | 29 | 38.6436597111 | 347 | 1.2e-94 |
| ABS75232.1 | DhbF | BGC0001185 | Bacillibactin\_biosynthetic\_gene\_cluster | 37 | 23.9165329053 | 347 | 1.6e-94 |
| ABM63529.1 | BryX | BGC0000174 | Bryostatin\_biosynthetic\_gene\_cluster | 32 | 28.6115569823 | 347 | 1.6e-94 |
| AMH40443.1 | PKS | BGC0001350 | Phormidolide\_biosynthetic\_gene\_cluster | 35 | 25.2407704655 | 347 | 1.6e-94 |
| KPN93063.1 | NupA | BGC0001416 | Nunapeptin\_/\_nunamycin\_biosynthetic\_gene\_cluster | 36 | 26.2439807384 | 346 | 2.1e-94 |
| AHH53507.1 | non-ribosomal\_peptide\_synthetase | BGC0000439 | Taromycin\_biosynthetic\_gene\_cluster | 38 | 23.3547351525 | 346 | 2.1e-94 |
| AAX31558.1 | peptide\_synthetase\_2 | BGC0000336 | Daptomycin\_biosynthetic\_gene\_cluster | 37 | 23.7158908507 | 346 | 2.7e-94 |
| AAY37647.1 | Amino\_acid\_adenylation | BGC0000437 | Syringomycin\_biosynthetic\_gene\_cluster | 38 | 25.3611556982 | 346 | 2.7e-94 |
| AFX60311.1 | polyketide\_synthase | BGC0001031 | Oocydin\_A\_biosynthetic\_gene\_cluster | 35 | 25.922953451 | 346 | 2.7e-94 |
| AAO62588.1 | peptide\_sythetase | BGC0001016 | Microcystin\_biosynthetic\_gene\_cluster | 36 | 24.2375601926 | 346 | 2.7e-94 |
| AHZ34238.1 | CipA | BGC0001389 | Cichopeptin\_biosynthetic\_gene\_cluster | 35 | 26.4446227929 | 346 | 2.7e-94 |
| AHZ34242.1 | CipE | BGC0001389 | Cichopeptin\_biosynthetic\_gene\_cluster | 35 | 26.5248796148 | 346 | 3.5e-94 |
| ADN68479.1 | SorD | BGC0000184 | Sorangicin\_biosynthetic\_gene\_cluster | 40 | 21.5489566613 | 344 | 7.9e-94 |
| CAK15815.1 | putative\_non\_ribosomal\_peptide\_synthetase | BGC0000344 | Entolysin\_biosynthetic\_gene\_cluster | 39 | 23.7158908507 | 344 | 7.9e-94 |
| CCT72377.1 | probable\_polyketide\_synthase | BGC0001305 | Fujikurins\_biosynthetic\_gene\_cluster | 36 | 28.0898876404 | 344 | 7.9e-94 |
| AGC45618.1 | non-ribosomal\_peptide\_synthetase | BGC0001394 | Phenalamide\_biosynthetic\_gene\_cluster | 37 | 23.7158908507 | 344 | 7.9e-94 |
| AAY32965.1 | DszB | BGC0001093 | Disorazole\_biosynthetic\_gene\_cluster | 37 | 22.3113964687 | 344 | 1e-93 |
| CAG23977.1 | polyketide\_synthase\_type\_I | BGC0000176 | Difficidin\_biosynthetic\_gene\_cluster | 33 | 26.9261637239 | 344 | 1e-93 |
| BAE93740.1 | type\_I\_polyketide\_synthase-related\_protein | BGC0000164 | Tetronomycin\_biosynthetic\_gene\_cluster | 36 | 26.886035313 | 344 | 1.3e-93 |
| CTQ34881.1 | AtcD;\_polyketide\_synthase,\_modules\_1-4 | BGC0001301 | Anthracimycin\_biosynthetic\_gene\_cluster | 36 | 23.0738362761 | 344 | 1.3e-93 |
| ACY01401.1 | AT-less\_polyketide\_synthase | BGC0000083 | Lactimidomycin\_biosynthetic\_gene\_cluster | 38 | 23.3146067416 | 343 | 1.8e-93 |
| BAH43870.1 | putative\_linear\_pentadecapeptide\_gramicidin\_synthetase\_LgrB | BGC0000367 | Gramicidin\_biosynthetic\_gene\_cluster | 36 | 23.9566613162 | 343 | 1.8e-93 |
| ADI59534.1 | CorL | BGC0001091 | Corallopyronin\_biosynthetic\_gene\_cluster | 37 | 21.6693418941 | 343 | 1.8e-93 |
| AIW82284.1 | PuwG | BGC0001125 | Puwainaphycins\_biosynthetic\_gene\_cluster | 35 | 23.9967897271 | 343 | 1.8e-93 |
| AAF00962.1 | McyC | BGC0001017 | Microcystin\_biosynthetic\_gene\_cluster | 34 | 24.1974317817 | 343 | 2.3e-93 |
| CAE15637.1 |  | BGC0001128 | Luminmide\_biosynthetic\_gene\_cluster | 36 | 26.4847512039 | 342 | 3e-93 |
| CAN89631.1 | putative\_polyketide\_synthase | BGC0001070 | Kirromycin\_biosynthetic\_gene\_cluster | 36 | 24.9598715891 | 342 | 3e-93 |
| AEP18656.1 | WAPS1 | BGC0000461 | WAP-8294A2\_(lotilibcin)\_biosynthetic\_gene\_cluster | 40 | 23.9165329053 | 342 | 3e-93 |
| ALD83686.1 | tAT\_polyketide\_synthase | BGC0001300 | Anthracimycin\_biosynthetic\_gene\_cluster | 37 | 22.191011236 | 342 | 3e-93 |
| ALV86867.1 | Tlo21 | BGC0001406 | Telomycin\_biosynthetic\_gene\_cluster | 37 | 24.6789727127 | 342 | 3e-93 |
| CAD29798.1 | peptide\_synthetase | BGC0001015 | Microcystin\_biosynthetic\_gene\_cluster | 34 | 24.1573033708 | 342 | 3.9e-93 |
| WP\_020635019.1 | hypothetical\_protein | BGC0001211 | Albachelin\_biosynthetic\_gene\_cluster | 38 | 24.7993579454 | 342 | 5.1e-93 |
| AFX60309.1 | polyketide\_synthase | BGC0001031 | Oocydin\_A\_biosynthetic\_gene\_cluster | 33 | 24.9598715891 | 342 | 5.1e-93 |
| CCJ67636.1 | TaaA | BGC0000447 | Tolaasin\_biosynthetic\_gene\_cluster | 37 | 23.9165329053 | 341 | 6.7e-93 |
| ADA82581.1 | trans-AT\_polyketide\_synthase | BGC0001110 | Psymberin\_biosynthetic\_gene\_cluster | 39 | 20.0240770465 | 341 | 8.7e-93 |
| AHZ34239.1 | CipB | BGC0001389 | Cichopeptin\_biosynthetic\_gene\_cluster | 34 | 26.5248796148 | 341 | 8.7e-93 |
| ABW17377.1 | PsoC | BGC0000411 | Putisolvin\_biosynthetic\_gene\_cluster | 37 | 24.036918138 | 341 | 8.7e-93 |
| AGN11882.1 | TstGH | BGC0001114 | Thailanstatin\_biosynthetic\_gene\_cluster | 37 | 23.1540930979 | 341 | 1.1e-92 |
| AFJ14793.1 | PlpD | BGC0000403 | Pelgipeptin\_biosynthetic\_gene\_cluster | 37 | 24.4382022472 | 341 | 1.1e-92 |
| AAY91420.2 | non-ribosomal\_peptide\_synthetase\_OfaB | BGC0000399 | Orfamide\_biosynthetic\_gene\_cluster | 38 | 24.2375601926 | 340 | 1.5e-92 |
| BAE98155.1 | putative\_non-ribosomal\_peptide\_synthetase | BGC0000339 | Echinomycin\_biosynthetic\_gene\_cluster | 38 | 22.8330658106 | 340 | 1.5e-92 |
| ACA09733.2 | fusaricidin\_synthetase | BGC0000358 | Fusaricidin\_biosynthetic\_gene\_cluster | 36 | 23.2343499197 | 340 | 1.5e-92 |
| ACZ55945.1 | non-ribosomal\_peptide\_synthetase | BGC0000302 | Anabaenopeptin\_biosynthetic\_gene\_cluster | 35 | 24.1573033708 | 340 | 1.5e-92 |
| AIC32694.1 | FR9GH | BGC0001113 | Spliceostatin\_/\_FR901464\_biosynthetic\_gene\_cluster | 35 | 25.0401284109 | 340 | 1.9e-92 |
| CCJ67637.1 | TaaB | BGC0000447 | Tolaasin\_biosynthetic\_gene\_cluster | 37 | 23.6757624398 | 340 | 1.9e-92 |
| AAS98786.1 | JamO | BGC0001001 | Jamaicamide\_biosynthetic\_gene\_cluster | 35 | 23.9967897271 | 340 | 1.9e-92 |
| AKC91857.1 | nonribosomal\_peptide\_synthetase | BGC0001414 | Griselimycin\_biosynthetic\_gene\_cluster | 37 | 23.9566613162 | 340 | 1.9e-92 |
| ABQ96384.2 | fusaricidin\_synthetase | BGC0001152 | Fusaricidin\_biosynthetic\_gene\_cluster | 36 | 23.3547351525 | 340 | 1.9e-92 |
| BAI63288.1 | putative\_non-ribosomal\_peptide\_synthetase | BGC0000434 | SW-163\_biosynthetic\_gene\_cluster | 39 | 22.9133226324 | 340 | 1.9e-92 |
| ADH01489.1 | type\_I\_polyketide\_synthase | BGC0001096 | FR901464\_biosynthetic\_gene\_cluster | 35 | 25.0401284109 | 340 | 1.9e-92 |
| AFX60334.1 | polyketide\_synthase | BGC0001032 | Oocydin\_A\_biosynthetic\_gene\_cluster | 35 | 24.6388443018 | 339 | 2.5e-92 |
| CCC21123.1 | type-I\_polyketide\_synthases | BGC0000171 | 9-methylstreptimidone\_biosynthetic\_gene\_cluster | 35 | 25.1605136437 | 339 | 2.5e-92 |
| ALV86866.1 | Tlo20 | BGC0001406 | Telomycin\_biosynthetic\_gene\_cluster | 38 | 23.9566613162 | 339 | 2.5e-92 |
| BAB69699.1 | iturin\_A\_synthetase\_B | BGC0001098 | Iturin\_biosynthetic\_gene\_cluster | 37 | 24.2776886035 | 339 | 2.5e-92 |
| ADD82941.1 | Bat3 | BGC0001099 | Kalimantacin\_/\_batumin\_biosynthetic\_gene\_cluster | 38 | 21.3884430177 | 339 | 2.5e-92 |
| AAV97870.1 | OnnB | BGC0001105 | Onnamide\_biosynthetic\_gene\_cluster | 35 | 23.7560192616 | 339 | 3.3e-92 |
| AMH40422.1 | PKS | BGC0001350 | Phormidolide\_biosynthetic\_gene\_cluster | 36 | 21.3884430177 | 339 | 3.3e-92 |
| AAY91421.3 | non-ribosomal\_peptide\_synthetase\_OfaC | BGC0000399 | Orfamide\_biosynthetic\_gene\_cluster | 36 | 26.3643659711 | 338 | 5.6e-92 |
| CAJ57409.1 | polyketide\_synthase\_type\_I | BGC0000176 | Difficidin\_biosynthetic\_gene\_cluster | 37 | 21.5088282504 | 338 | 5.6e-92 |
| AFX60332.1 | polyketide\_synthase | BGC0001032 | Oocydin\_A\_biosynthetic\_gene\_cluster | 33 | 25.2407704655 | 338 | 5.6e-92 |
| BAH43765.1 | tyrocidine\_synthetase\_II | BGC0000452 | Tyrocidine\_biosynthetic\_gene\_cluster | 34 | 24.077046549 | 338 | 7.4e-92 |
| AEH59109.1 | keto-hydroxyglutarate-aldolase/polyketide\_synthase | BGC0000385 | Lysobactin\_biosynthetic\_gene\_cluster | 38 | 22.5521669342 | 338 | 7.4e-92 |
| ALD83703.1 | tAT\_polyketide\_synthase | BGC0001299 | Chlorotonil\_biosynthetic\_gene\_cluster | 39 | 20.7062600321 | 338 | 7.4e-92 |
| CAG23964.1 | polyketide\_synthase\_type\_I | BGC0000181 | Macrolactin\_biosynthetic\_gene\_cluster | 34 | 26.0032102729 | 337 | 9.6e-92 |
| AFH75321.1 | nonribosomal\_peptide\_synthetase | BGC0000425 | Sessilin\_biosynthetic\_gene\_cluster | 37 | 23.3547351525 | 337 | 9.6e-92 |
| AGE11899.1 | nonribosomal\_peptide\_synthetase | BGC0000366 | Gobichelin\_biosynthetic\_gene\_cluster | 39 | 24.1171749599 | 337 | 9.6e-92 |
| AEZ51516.1 | PmxA | BGC0001153 | Polymyxin\_biosynthetic\_gene\_cluster | 37 | 23.7158908507 | 337 | 9.6e-92 |
| AHH25592.1 | NRPS | BGC0000957 | Ansatrienin\_(mycotrienin)\_biosynthetic\_gene\_cluster | 39 | 23.3146067416 | 337 | 1.3e-91 |
| AAF00961.1 | McyB | BGC0001017 | Microcystin\_biosynthetic\_gene\_cluster | 35 | 24.2375601926 | 337 | 1.3e-91 |
| AEU11003.1 | NpnC | BGC0001029 | Nostophycin\_biosynthetic\_gene\_cluster | 36 | 23.6356340289 | 337 | 1.6e-91 |
| ALV82384.1 | CDA\_peptide\_synthetase\_II | BGC0001370 | Lipopeptide\_8D1-1\_/\_lipopeptide\_8D1-2\_biosynthetic\_gene\_cluster | 38 | 23.3547351525 | 336 | 2.1e-91 |
| AGM16413.1 | paenibacterin\_synthetase\_B | BGC0000400 | Paenibacterin\_biosynthetic\_gene\_cluster | 35 | 24.5585874799 | 336 | 2.8e-91 |
| AAZ03552.1 | McnC | BGC0000332 | Cyanopeptolin\_biosynthetic\_gene\_cluster | 36 | 24.2776886035 | 336 | 2.8e-91 |
| BAP05597.1 | CalI | BGC0000967 | Calyculin\_biosynthetic\_gene\_cluster | 35 | 24.7592295345 | 336 | 2.8e-91 |
| AAC06348.1 | bacitracin\_synthetase\_3 | BGC0000310 | Bacitracin\_biosynthetic\_gene\_cluster | 34 | 25.5216693419 | 336 | 2.8e-91 |
| CAN89634.1 | putative\_polyketide\_synthase | BGC0001070 | Kirromycin\_biosynthetic\_gene\_cluster | 35 | 26.8057784912 | 336 | 2.8e-91 |
| CAD29799.1 | microcystin\_synthetase | BGC0001015 | Microcystin\_biosynthetic\_gene\_cluster | 34 | 24.1974317817 | 336 | 2.8e-91 |
| AMH40421.1 | PKS | BGC0001350 | Phormidolide\_biosynthetic\_gene\_cluster | 34 | 26.2439807384 | 336 | 2.8e-91 |
| ADN68477.1 | SorB | BGC0000184 | Sorangicin\_biosynthetic\_gene\_cluster | 38 | 21.227929374 | 336 | 3.7e-91 |
| AIJ04684.1 | polyketide\_synthase | BGC0001383 | Macrolactin\_biosynthetic\_gene\_cluster | 34 | 23.9566613162 | 336 | 3.7e-91 |
| AAU39359.1 | lichenysin\_synthase\_LchAA | BGC0000381 | Lichenysin\_biosynthetic\_gene\_cluster | 36 | 25.6821829856 | 336 | 3.7e-91 |
| ALD83704.1 | tAT\_polyketide\_synthase | BGC0001299 | Chlorotonil\_biosynthetic\_gene\_cluster | 38 | 21.6693418941 | 336 | 3.7e-91 |
| ABS74180.1 | BmyB | BGC0001090 | Bacillomycin\_biosynthetic\_gene\_cluster | 36 | 24.2776886035 | 335 | 4.8e-91 |
| ACM79812.1 | ZmaQ | BGC0001059 | Zwittermycin\_A\_biosynthetic\_gene\_cluster | 33 | 24.3980738363 | 335 | 6.2e-91 |
| CAN93347.1 | Polyketide\_synthase | BGC0000179 | Etnangien\_biosynthetic\_gene\_cluster | 40 | 21.4686998395 | 335 | 6.2e-91 |
| BAH43869.1 | linear\_pentadecapeptide\_gramicidin\_synthetase\_LgrA | BGC0000367 | Gramicidin\_biosynthetic\_gene\_cluster | 35 | 23.9566613162 | 335 | 6.2e-91 |
| AGM16414.1 | paenibacterin\_synthetase\_C | BGC0000400 | Paenibacterin\_biosynthetic\_gene\_cluster | 34 | 25.0 | 335 | 6.2e-91 |
| BAH43871.1 | truncated\_linear\_pentadecapeptide\_gramicidin\_synthetase\_LgrC | BGC0000367 | Gramicidin\_biosynthetic\_gene\_cluster | 36 | 23.6757624398 | 334 | 8.1e-91 |
| AIJ04681.1 | polyketide\_synthase | BGC0001383 | Macrolactin\_biosynthetic\_gene\_cluster | 34 | 26.0032102729 | 334 | 8.1e-91 |
| ACA97576.1 | PmxA | BGC0000408 | Polymyxin\_biosynthetic\_gene\_cluster | 36 | 23.7158908507 | 334 | 8.1e-91 |
| ERM18797.1 | polyketide\_synthase | BGC0000172 | Basiliskamides\_biosynthetic\_gene\_cluster | 32 | 27.2873194222 | 334 | 8.1e-91 |
| CAG23967.1 | polyketide\_synthase\_type\_I | BGC0000181 | Macrolactin\_biosynthetic\_gene\_cluster | 34 | 23.9566613162 | 334 | 1.1e-90 |
| CDG17980.1 | Putative\_Ornithine\_racemase\_(fragment) | BGC0000464 | Xenoamicins\_biosynthetic\_gene\_cluster | 34 | 26.0834670947 | 334 | 1.1e-90 |
| BAH22765.1 | nonribosomal\_peptide\_synthetase | BGC0001018 | Micropeptin\_biosynthetic\_gene\_cluster | 35 | 24.5987158909 | 334 | 1.4e-90 |
| AAZ03554.1 | McnE | BGC0000332 | Cyanopeptolin\_biosynthetic\_gene\_cluster | 35 | 24.5987158909 | 333 | 1.8e-90 |
| AFO59866.1 | ChxE | BGC0000175 | Cycloheximide\_/\_actiphenol\_biosynthetic\_gene\_cluster | 35 | 27.1268057785 | 333 | 1.8e-90 |
| CAJ14037.1 | peptide\_synthetase\_PhsB | BGC0000406 | Phosphinothricin\_biosynthetic\_gene\_cluster | 38 | 22.8330658106 | 333 | 1.8e-90 |
| AIW82283.1 | PuwF | BGC0001125 | Puwainaphycins\_biosynthetic\_gene\_cluster | 36 | 24.5585874799 | 333 | 2.4e-90 |
| ABC34832.1 | polyketide\_synthase | BGC0000186 | Thailandamide\_biosynthetic\_gene\_cluster | 39 | 20.7865168539 | 333 | 2.4e-90 |
| ABI26078.1 | OciB | BGC0000331 | Cyanopeptin\_biosynthetic\_gene\_cluster | 35 | 24.5987158909 | 333 | 2.4e-90 |
| AED90002.1 | non-ribosomal\_peptide\_synthetase\_ThaA | BGC0000443 | Thanamycin\_biosynthetic\_gene\_cluster | 36 | 24.1974317817 | 332 | 3.1e-90 |
| AMK48226.1 | nonribosomal\_peptide\_synthetase | BGC0001351 | Cahuitamycins\_biosynthetic\_gene\_cluster | 38 | 24.6789727127 | 332 | 3.1e-90 |
| ADI59532.1 | CorJ | BGC0001091 | Corallopyronin\_biosynthetic\_gene\_cluster | 35 | 26.6853932584 | 332 | 3.1e-90 |
| CAE53350.1 | non-ribosomal\_peptide\_synthetase | BGC0000440 | Teicoplanin\_biosynthetic\_gene\_cluster | 38 | 23.5152487961 | 332 | 3.1e-90 |
| AIU36103.1 | LglD | BGC0000180 | Legioliulin\_biosynthetic\_gene\_cluster | 35 | 23.2343499197 | 332 | 3.1e-90 |
| AFK83820.1 | PtzD | BGC0001107\_c2 | Patellazole\_biosynthetic\_gene\_cluster | 38 | 20.8667736758 | 332 | 4e-90 |
| ABW17375.1 | PsoA | BGC0000411 | Putisolvin\_biosynthetic\_gene\_cluster | 39 | 24.077046549 | 332 | 4e-90 |
| AKC91849.1 | nonribosomal\_peptide\_synthetase | BGC0001414 | Griselimycin\_biosynthetic\_gene\_cluster | 38 | 24.1573033708 | 332 | 4e-90 |
| CAJ46691.1 | non\_ribosomal\_peptide\_synthase | BGC0000969 | Chondramide\_biosynthetic\_gene\_cluster | 36 | 23.7158908507 | 332 | 4e-90 |
| ABX37385.1 | amino\_acid\_adenylation\_domain\_protein | BGC0000984 | Delftibactin\_biosynthetic\_gene\_cluster | 38 | 23.2744783307 | 332 | 5.3e-90 |
| AAY32966.1 | DszC | BGC0001093 | Disorazole\_biosynthetic\_gene\_cluster | 36 | 25.3611556982 | 332 | 5.3e-90 |
| CTQ34883.1 | AtcF;\_polyketide\_synthase,\_modules\_8-10 | BGC0001301 | Anthracimycin\_biosynthetic\_gene\_cluster | 37 | 21.5890850722 | 331 | 6.9e-90 |
| CCJ67640.1 | TaaE | BGC0000447 | Tolaasin\_biosynthetic\_gene\_cluster | 35 | 24.4783306581 | 331 | 6.9e-90 |
| BAC67536.1 | arthrofactin\_synthetase\_C | BGC0000305 | Arthrofactin\_biosynthetic\_gene\_cluster | 36 | 24.5585874799 | 331 | 6.9e-90 |
| AFB35635.1 | EsmB4 | BGC0000935 | Esmeraldin\_biosynthetic\_gene\_cluster | 35 | 27.0866773676 | 331 | 6.9e-90 |
| AJM89735.1 | PmxA | BGC0001192 | Colistin\_biosynthetic\_gene\_cluster | 36 | 23.595505618 | 331 | 6.9e-90 |
| AFH75329.1 | nonribosomal\_peptide\_synthetase | BGC0000398 | Orfamide\_biosynthetic\_gene\_cluster | 38 | 24.077046549 | 331 | 9e-90 |
| AGM16412.1 | paenibacterin\_synthetase\_A | BGC0000400 | Paenibacterin\_biosynthetic\_gene\_cluster | 33 | 26.404494382 | 331 | 9e-90 |
| CCJ67639.1 | TaaD | BGC0000447 | Tolaasin\_biosynthetic\_gene\_cluster | 36 | 23.9566613162 | 331 | 9e-90 |
| ctg1\_orf6 |  | BGC0001109 | Pederin\_biosynthetic\_gene\_cluster | 36 | 22.7528089888 | 331 | 9e-90 |
| AAS47564.1 | mixed\_type\_I\_polyketide\_synthase/nonribosomal\_peptide\_synthetase | BGC0001108 | Pederin\_biosynthetic\_gene\_cluster | 36 | 22.7528089888 | 331 | 9e-90 |
| AKQ22698.1 | malonyl\_CoA-acyl\_carrier\_protein\_transacylase | BGC0001186 | Misakinolide\_biosynthetic\_gene\_cluster | 38 | 20.9069020867 | 331 | 1.2e-89 |
| CAG15009.1 | peptide\_synthetase,\_module\_1-2 | BGC0000441 | Teicoplanin\_biosynthetic\_gene\_cluster | 38 | 23.5152487961 | 330 | 1.5e-89 |
| AHJ31215.1 | Long-chain-fatty-acid--CoA\_ligase | BGC0000430 | Spumigin\_biosynthetic\_gene\_cluster | 35 | 24.7191011236 | 330 | 1.5e-89 |
| AAU34203.1 | mannopeptimycin\_peptide\_synthetase\_MppB | BGC0000388 | Mannopeptimycin\_biosynthetic\_gene\_cluster | 37 | 24.2375601926 | 330 | 1.5e-89 |
| AAC06346.1 | bacitracin\_synthetase\_1 | BGC0000310 | Bacitracin\_biosynthetic\_gene\_cluster | 34 | 24.3579454254 | 330 | 1.5e-89 |
| AIW82277.1 | PuwA | BGC0001125 | Puwainaphycins\_biosynthetic\_gene\_cluster | 35 | 24.2776886035 | 330 | 1.5e-89 |
| AAU34202.1 | mannopeptimycin\_peptide\_synthetase\_MppA | BGC0000388 | Mannopeptimycin\_biosynthetic\_gene\_cluster | 37 | 23.1942215088 | 330 | 1.5e-89 |
| CBK62724.1 |  | BGC0001115 | Thiomarinol\_biosynthetic\_gene\_cluster | 34 | 25.3611556982 | 330 | 2e-89 |
| ADN68482.1 | SorG | BGC0000184 | Sorangicin\_biosynthetic\_gene\_cluster | 38 | 22.150882825 | 330 | 2e-89 |
| ABI26077.1 | OciA | BGC0000331 | Cyanopeptin\_biosynthetic\_gene\_cluster | 35 | 24.9598715891 | 330 | 2e-89 |
| CDG17985.1 | Putative\_Ornithine\_racemase\_(fragment) | BGC0000464 | Xenoamicins\_biosynthetic\_gene\_cluster | 35 | 26.404494382 | 330 | 2e-89 |
| AAF08796.1 | MycB | BGC0001103 | Mycosubtilin\_biosynthetic\_gene\_cluster | 35 | 23.3547351525 | 329 | 2.6e-89 |
| AAY89051.1 | hybrid\_nonribosomal\_peptide\_synthetase/polyketide\_synthase | BGC0001069 | Chivosazole\_biosynthetic\_gene\_cluster | 37 | 25.0 | 329 | 2.6e-89 |
| ALG65317.1 | Cal19 | BGC0001297 | WS9326\_biosynthetic\_gene\_cluster | 38 | 23.6757624398 | 329 | 2.6e-89 |
| CAN93349.1 | polyketide\_synthase | BGC0000179 | Etnangien\_biosynthetic\_gene\_cluster | 38 | 22.2311396469 | 329 | 4.5e-89 |
| ACM68684.1 | AerB | BGC0000298 | Aeruginosin\_biosynthetic\_gene\_cluster | 34 | 23.9165329053 | 328 | 5.8e-89 |
| AAF08797.1 | MycC | BGC0001103 | Mycosubtilin\_biosynthetic\_gene\_cluster | 35 | 23.1540930979 | 328 | 7.6e-89 |
| AFH75322.1 | nonribosomal\_peptide\_synthetase | BGC0000425 | Sessilin\_biosynthetic\_gene\_cluster | 35 | 24.077046549 | 328 | 7.6e-89 |
| ACR13065.1 | modular\_polyketide\_synthase,\_type\_I\_PKS | BGC0000185 | Tartrolon\_biosynthetic\_gene\_cluster | 37 | 21.4686998395 | 328 | 7.6e-89 |
| BAD55611.1 | putative\_non-ribosomal\_peptide\_synthetase | BGC0001027 | Nocobactin\_NA\_biosynthetic\_gene\_cluster | 36 | 25.2808988764 | 328 | 7.6e-89 |
| AFX60336.1 | polyketide\_synthase | BGC0001032 | Oocydin\_A\_biosynthetic\_gene\_cluster | 37 | 21.3483146067 | 328 | 7.6e-89 |
| CCJ67648.1 | JagD | BGC0001127 | Jagaricin\_biosynthetic\_gene\_cluster | 37 | 24.3178170144 | 327 | 1e-88 |
| ERM18798.1 | polyketide\_synthase | BGC0000172 | Basiliskamides\_biosynthetic\_gene\_cluster | 38 | 20.5457463884 | 327 | 1e-88 |
| ABF92489.1 | mixed\_type\_I\_polyketide\_synthase\_-\_peptide\_synthetase | BGC0001025 | Myxovirescin\_biosynthetic\_gene\_cluster | 34 | 25.7624398074 | 327 | 1e-88 |
| BAC67534.2 | arthrofactin\_synthetase\_A | BGC0000305 | Arthrofactin\_biosynthetic\_gene\_cluster | 37 | 24.036918138 | 327 | 1e-88 |
| ADA69237.1 | trans-AT\_polyketide\_synthase | BGC0001071 | Nosperin\_biosynthetic\_gene\_cluster | 37 | 21.9101123596 | 327 | 1e-88 |
| AJM89738.1 | PmxE | BGC0001192 | Colistin\_biosynthetic\_gene\_cluster | 37 | 23.9566613162 | 327 | 1.3e-88 |
| AGN71604.1 | conidial\_yellow\_pigment\_biosynthesis\_polyketide\_synthase | BGC0000027 | Azaphilone\_biosynthetic\_gene\_cluster | 29 | 37.3996789727 | 327 | 1.7e-88 |
| AAM80539.1 | StaA | BGC0000290 | A47934\_biosynthetic\_gene\_cluster | 38 | 23.6757624398 | 327 | 1.7e-88 |
| CAE02631.1 | surfactin\_synthetase\_B | BGC0000433 | Surfactin\_biosynthetic\_gene\_cluster | 35 | 24.1171749599 | 327 | 1.7e-88 |
| AMH40423.1 | PKS | BGC0001350 | Phormidolide\_biosynthetic\_gene\_cluster | 35 | 24.6789727127 | 327 | 1.7e-88 |
| AAF99707.2 | syringopeptin\_synthetase | BGC0000438 | Syringopeptin\_biosynthetic\_gene\_cluster | 35 | 26.2038523274 | 327 | 1.7e-88 |
| AHI59108.1 | locillomycin\_synthase\_A | BGC0001005 | Locillomycin\_biosynthetic\_gene\_cluster | 34 | 24.1171749599 | 326 | 2.2e-88 |
| ABV79985.1 | ApnA | BGC0000301 | Anabaenopeptin\_biosynthetic\_gene\_cluster | 33 | 24.3178170144 | 326 | 2.9e-88 |
| ACR50796.1 | putative\_polyketide\_synthase | BGC0000163 | Tetronasin\_biosynthetic\_gene\_cluster | 36 | 24.4783306581 | 326 | 2.9e-88 |
| AGZ15458.1 | putative\_non-ribosomal\_peptide\_synthetase | BGC0001036 | Polyoxypeptin\_biosynthetic\_gene\_cluster | 38 | 23.6757624398 | 326 | 2.9e-88 |
| CAL69893.1 | RhiE\_protein | BGC0001112 | Rhizoxins\_biosynthetic\_gene\_cluster | 34 | 24.7191011236 | 326 | 3.8e-88 |
| AIU36104.1 | LglE | BGC0000180 | Legioliulin\_biosynthetic\_gene\_cluster | 36 | 21.7897271268 | 326 | 3.8e-88 |
| ABM63527.1 | BryB | BGC0000174 | Bryostatin\_biosynthetic\_gene\_cluster | 36 | 21.9502407705 | 325 | 4.9e-88 |
| BAC76474.1 | type\_I\_polyketide\_synthase\_LkcC | BGC0001100 | Lankacidin\_biosynthetic\_gene\_cluster | 33 | 27.4077046549 | 325 | 4.9e-88 |
| CBX87032.1 | BcBOA9\_protein | BGC0001243\_c3 | Botryenalol\_/\_botrydial\_/\_dihydrobotrydial\_/\_botryendial\_/ | 29 | 35.2327447833 | 325 | 4.9e-88 |
| AAY89050.1 | polyketide\_synthase | BGC0001069 | Chivosazole\_biosynthetic\_gene\_cluster | 38 | 21.5489566613 | 325 | 4.9e-88 |
| CAJ21198.2 | non-ribosomal\_peptide\_synthetase | BGC0000297 | Aeruginoside\_biosynthetic\_gene\_cluster | 34 | 24.5585874799 | 325 | 4.9e-88 |
| CAG23958.2 | polyketide\_synthase | BGC0001089 | Bacillaene\_biosynthetic\_gene\_cluster | 35 | 23.113964687 | 325 | 6.5e-88 |
| CAN93352.1 | polyketide\_synthase | BGC0000179 | Etnangien\_biosynthetic\_gene\_cluster | 36 | 22.2311396469 | 325 | 6.5e-88 |
| AAU39361.1 | lichenysin\_synthase\_LchAC | BGC0000381 | Lichenysin\_biosynthetic\_gene\_cluster | 36 | 23.4751203852 | 324 | 8.4e-88 |
| AEW31021.1 | plipastatin\_synthetase | BGC0000407 | Plipastatin\_biosynthetic\_gene\_cluster | 34 | 23.5553772071 | 324 | 8.4e-88 |
| AEC04363.1 | polyketide\_synthase | BGC0000178 | Elansolid\_biosynthetic\_gene\_cluster | 37 | 21.4285714286 | 324 | 8.4e-88 |
| AEC04357.1 | polyketide\_synthase | BGC0000178 | Elansolid\_biosynthetic\_gene\_cluster | 35 | 22.8731942215 | 324 | 1.1e-87 |
| AFJ23819.1 | WLIP\_synthetase\_A | BGC0000462 | WLIP\_biosynthetic\_gene\_cluster | 39 | 23.1942215088 | 324 | 1.1e-87 |
| CBJ90082.1 | Non\_Ribosomal\_peptide\_synthetase\_(-succinylbenzoate--CoA\_ligase) | BGC0001132 | Xenotetrapeptide\_biosynthetic\_gene\_cluster | 33 | 26.2841091493 | 324 | 1.1e-87 |
| CBG75492.1 | putative\_NRPS/siderophore\_biosynthesis\_protein | BGC0000423 | Scabichelin\_biosynthetic\_gene\_cluster | 37 | 25.8426966292 | 324 | 1.1e-87 |
| AFP87549.1 | NrpS | BGC0001135 | Bicornutin\_biosynthetic\_gene\_cluster | 38 | 23.9165329053 | 324 | 1.1e-87 |
| BAP05594.1 | CalF | BGC0000967 | Calyculin\_biosynthetic\_gene\_cluster | 36 | 22.8330658106 | 324 | 1.4e-87 |
| AGS06823.1 | type\_I\_polyketide\_synthase,\_PedI-like\_protein | BGC0001092 | Diaphorin\_biosynthetic\_gene\_cluster | 32 | 25.2808988764 | 324 | 1.4e-87 |
| CDG17987.1 | Putative\_Ornithine\_racemase\_(fragment) | BGC0000464 | Xenoamicins\_biosynthetic\_gene\_cluster | 34 | 26.6051364366 | 323 | 1.9e-87 |
| AEZ51520.1 | PmxE | BGC0001153 | Polymyxin\_biosynthetic\_gene\_cluster | 37 | 23.9165329053 | 323 | 1.9e-87 |
| ABD14711.1 | CesA | BGC0000320 | Cereulide\_biosynthetic\_gene\_cluster | 34 | 24.077046549 | 323 | 1.9e-87 |
| AAM12909.2 | MmpA | BGC0000182 | Mupirocin\_biosynthetic\_gene\_cluster | 33 | 27.1669341894 | 323 | 2.5e-87 |
| ACA97580.1 | PmxE | BGC0000408 | Polymyxin\_biosynthetic\_gene\_cluster | 37 | 23.9165329053 | 323 | 2.5e-87 |
| AFJ14795.1 | PlpF | BGC0000403 | Pelgipeptin\_biosynthetic\_gene\_cluster | 35 | 24.2375601926 | 323 | 2.5e-87 |
| ABC33986.1 | polyketide\_synthase,\_putative | BGC0000186 | Thailandamide\_biosynthetic\_gene\_cluster | 37 | 22.1107544141 | 323 | 2.5e-87 |
| BAH22764.1 | nonribosomal\_peptide\_synthetase | BGC0001018 | Micropeptin\_biosynthetic\_gene\_cluster | 34 | 24.6388443018 | 322 | 3.2e-87 |
| ABI26079.1 | OciC | BGC0000331 | Cyanopeptin\_biosynthetic\_gene\_cluster | 34 | 24.1573033708 | 322 | 4.2e-87 |
| AFN27482.1 | pks\_BonC | BGC0000173 | Bongkrekic\_acid\_biosynthetic\_gene\_cluster | 37 | 22.8731942215 | 322 | 4.2e-87 |
| CAD91212.1 | putative\_non-ribosomal\_peptide\_synthetase,\_modules\_4-6 | BGC0000289 | A40926\_biosynthetic\_gene\_cluster | 37 | 23.8764044944 | 322 | 4.2e-87 |
| AFX60313.1 | polyketide\_synthase | BGC0001031 | Oocydin\_A\_biosynthetic\_gene\_cluster | 38 | 22.191011236 | 322 | 5.5e-87 |
| CAJ76290.1 | putative\_non-ribosomal\_peptide\_synthase | BGC0000972 | Colibactin\_biosynthetic\_gene\_cluster | 35 | 23.113964687 | 322 | 5.5e-87 |
| ADJ63842.1 | non-ribosomal\_peptide\_synthetase\_protein | BGC0000424 | Serobactins\_biosynthetic\_gene\_cluster | 38 | 23.6757624398 | 322 | 5.5e-87 |
| AIE77059.1 | peptide\_synthetase | BGC0000418 | Ristocetin\_biosynthetic\_gene\_cluster | 37 | 23.3547351525 | 322 | 5.5e-87 |
| ABW00331.1 | amino\_acid\_adenylation\_domain | BGC0000333 | Cyclomarin\_biosynthetic\_gene\_cluster | 37 | 23.9566613162 | 322 | 5.5e-87 |
| ADN68483.1 | SorH | BGC0000184 | Sorangicin\_biosynthetic\_gene\_cluster | 40 | 21.0272873194 | 321 | 7.1e-87 |
| AKQ22697.1 | malonyl\_CoA-acyl\_carrier\_protein\_transacylase | BGC0001186 | Misakinolide\_biosynthetic\_gene\_cluster | 33 | 23.9967897271 | 321 | 7.1e-87 |
| AIG79241.1 | Hypothetical\_protein | BGC0000419 | Ristomycin\_A\_biosynthetic\_gene\_cluster | 37 | 23.0738362761 | 321 | 7.1e-87 |
| ADD82939.1 | Bat1 | BGC0001099 | Kalimantacin\_/\_batumin\_biosynthetic\_gene\_cluster | 38 | 20.746388443 | 321 | 7.1e-87 |
| AHI59107.1 | locillomycin\_synthase\_D | BGC0001005 | Locillomycin\_biosynthetic\_gene\_cluster | 30 | 34.430176565 | 321 | 7.1e-87 |
| ABS74207.1 | FenC | BGC0001095 | Fengycin\_biosynthetic\_gene\_cluster | 35 | 24.3178170144 | 321 | 9.3e-87 |
| CBK62733.1 |  | BGC0001115 | Thiomarinol\_biosynthetic\_gene\_cluster | 37 | 20.9871589085 | 321 | 9.3e-87 |
| AHI59110.1 | locillomycin\_synthase\_C | BGC0001005 | Locillomycin\_biosynthetic\_gene\_cluster | 33 | 25.2407704655 | 321 | 1.2e-86 |
| AGE26255.1 | putative\_non-ribosomal\_peptide\_synthetase | BGC0001208 | Poaeamide\_biosynthetic\_gene\_cluster | 38 | 23.3948635634 | 321 | 1.2e-86 |
| AAF15891.2 | NosA | BGC0001028 | Nostopeptolide\_biosynthetic\_gene\_cluster | 34 | 24.518459069 | 321 | 1.2e-86 |
| ABF88102.1 | polyketide\_synthase | BGC0001025 | Myxovirescin\_biosynthetic\_gene\_cluster | 38 | 20.6661316212 | 320 | 1.6e-86 |
| CAL69889.1 | RhiB\_protein | BGC0001112 | Rhizoxins\_biosynthetic\_gene\_cluster | 33 | 25.7223113965 | 320 | 1.6e-86 |
| AHH34189.1 | polyketide\_synthase | BGC0001162 | 1-heptadecene\_biosynthetic\_gene\_cluster | 34 | 24.9197431782 | 320 | 2.1e-86 |
| ACM79806.1 | ZmaB | BGC0001059 | Zwittermycin\_A\_biosynthetic\_gene\_cluster | 35 | 23.9566613162 | 320 | 2.1e-86 |
| AAZ23077.1 | peptide\_synthetase | BGC0000291 | A54145\_biosynthetic\_gene\_cluster | 37 | 24.4382022472 | 319 | 2.7e-86 |
| AAY93445.1 | non-ribosomal\_peptide\_synthetase\_PvdL | BGC0000413 | Pyoverdine\_biosynthetic\_gene\_cluster | 36 | 23.2744783307 | 319 | 2.7e-86 |
| AAY37654.1 | Amino\_acid\_adenylation | BGC0000437 | Syringomycin\_biosynthetic\_gene\_cluster | 34 | 26.1637239165 | 319 | 2.7e-86 |
| ALK27915.1 | non-ribosomal\_peptide\_synthase | BGC0001233 | Feglymycin\_biosynthetic\_gene\_cluster | 37 | 23.2744783307 | 319 | 2.7e-86 |
| ABS90475.1 | PKS | BGC0001106 | Oxazolomycin\_biosynthetic\_gene\_cluster | 34 | 26.9662921348 | 319 | 2.7e-86 |
| ACY01400.1 | AT-less\_polyketide\_synthase | BGC0000083 | Lactimidomycin\_biosynthetic\_gene\_cluster | 36 | 22.2311396469 | 319 | 3.5e-86 |
| AKQ22696.1 | malonyl\_CoA-acyl\_carrier\_protein\_transacylase | BGC0001186 | Misakinolide\_biosynthetic\_gene\_cluster | 33 | 26.0433386838 | 319 | 3.5e-86 |
| AAN85522.1 | hybrid\_nonribosomal\_peptide\_synthetase\_/\_polyketide\_synthase | BGC0001101 | Leinamycin\_biosynthetic\_gene\_cluster | 35 | 26.6452648475 | 319 | 3.5e-86 |
| AEW31020.1 | plipastatin\_synthetase | BGC0000407 | Plipastatin\_biosynthetic\_gene\_cluster | 33 | 24.036918138 | 319 | 3.5e-86 |
| AAO72424.1 | syringopeptin\_synthetase\_B | BGC0000438 | Syringopeptin\_biosynthetic\_gene\_cluster | 34 | 26.1637239165 | 319 | 4.6e-86 |
| CAN93351.1 | polyketide\_synthase | BGC0000179 | Etnangien\_biosynthetic\_gene\_cluster | 35 | 23.1540930979 | 319 | 4.6e-86 |
| ABI22132.1 | putative\_non-ribosomal\_peptide\_synthetase | BGC0000422 | Saframycin\_A\_biosynthetic\_gene\_cluster | 37 | 21.9502407705 | 319 | 4.6e-86 |
| ALD82526.1 | non-ribosomal\_peptide\_synthase | BGC0001212 | Nannocystin\_biosynthetic\_gene\_cluster | 36 | 23.8764044944 | 319 | 4.6e-86 |
| ABS74206.1 | FenD | BGC0001095 | Fengycin\_biosynthetic\_gene\_cluster | 34 | 24.3579454254 | 318 | 6e-86 |
| AEC04361.1 | polyketide\_synthase | BGC0000178 | Elansolid\_biosynthetic\_gene\_cluster | 36 | 21.3884430177 | 318 | 6e-86 |
| CAA11795.1 | PCZA363.4 | BGC0000322 | Chloroeremomycin\_biosynthetic\_gene\_cluster | 37 | 23.3948635634 | 318 | 6e-86 |
| AGD80623.1 | non-ribosomal\_peptide\_synthetase | BGC0000394 | Naphthyridinomycin\_biosynthetic\_gene\_cluster | 36 | 24.3579454254 | 318 | 7.9e-86 |
| AAU39360.1 | lichenysin\_synthase\_LchAB | BGC0000381 | Lichenysin\_biosynthetic\_gene\_cluster | 34 | 23.7560192616 | 318 | 7.9e-86 |
| AEC14347.1 | nonribosomal\_peptide\_synthetase | BGC0000377 | Koranimine\_biosynthetic\_gene\_cluster | 35 | 23.5553772071 | 318 | 7.9e-86 |
| AEP18655.1 | WAPS2 | BGC0000461 | WAP-8294A2\_(lotilibcin)\_biosynthetic\_gene\_cluster | 37 | 24.2375601926 | 318 | 7.9e-86 |
| AEI58866.1 | peptide\_synthetase | BGC0000455 | Vancomycin\_biosynthetic\_gene\_cluster | 35 | 24.5585874799 | 318 | 7.9e-86 |
| CAN93348.1 | polyketide\_synthase | BGC0000179 | Etnangien\_biosynthetic\_gene\_cluster | 33 | 28.0497592295 | 317 | 1e-85 |
| CAG23978.1 | polyketide\_synthase\_type\_I | BGC0000176 | Difficidin\_biosynthetic\_gene\_cluster | 36 | 22.0304975923 | 317 | 1e-85 |
| ABC34675.1 | polyketide\_synthase,\_putative | BGC0000186 | Thailandamide\_biosynthetic\_gene\_cluster | 36 | 23.113964687 | 317 | 1e-85 |
| CAJ96470.1 | Non-ribosomal\_peptide\_synthetase | BGC0000330 | Cupriachelin\_biosynthetic\_gene\_cluster | 37 | 23.3948635634 | 317 | 1e-85 |
| AFN27483.1 | pks\_BonD | BGC0000173 | Bongkrekic\_acid\_biosynthetic\_gene\_cluster | 36 | 22.0304975923 | 317 | 1.3e-85 |
| ACG60781.1 | PKS(KS/AT/MT/KR/ACP) | BGC0001058 | Zorbamycin\_biosynthetic\_gene\_cluster | 34 | 27.6083467095 | 317 | 1.3e-85 |
| AAF17281.1 | NosD | BGC0001028 | Nostopeptolide\_biosynthetic\_gene\_cluster | 35 | 24.5585874799 | 317 | 1.3e-85 |
| XP\_001220460.1 | hypothetical\_protein | BGC0001182 | Chaetoglobosins\_biosynthetic\_gene\_cluster | 32 | 29.9357945425 | 317 | 1.3e-85 |
| ACZ55942.1 | non-ribosomal\_peptide\_synthetase | BGC0000302 | Anabaenopeptin\_biosynthetic\_gene\_cluster | 33 | 23.9566613162 | 317 | 1.3e-85 |
| ANQ43347.1 | non-ribosomal\_peptide\_synthetase | BGC0001347 | Poaeamide\_B\_biosynthetic\_gene\_cluster | 38 | 23.3547351525 | 317 | 1.8e-85 |
| AHZ34233.1 | CifB | BGC0000323 | Cichofactin\_biosynthetic\_gene\_cluster | 36 | 25.6420545746 | 316 | 2.3e-85 |
| AOA33123.1 | nonribosomal\_peptide\_synthetase | BGC0001346 | Bananamides\_biosynthetic\_gene\_cluster | 36 | 25.481540931 | 316 | 2.3e-85 |
| AHD05677.1 | nonribosomal\_peptide\_ligase\_subunit | BGC0000402 | Paenilarvins\_biosynthetic\_gene\_cluster | 35 | 24.2776886035 | 316 | 2.3e-85 |
| AAC06347.1 | bacitracin\_synthetase\_2 | BGC0000310 | Bacitracin\_biosynthetic\_gene\_cluster | 34 | 24.3980738363 | 316 | 3e-85 |
| CBZ42143.1 | putative\_non-ribosomal\_peptide\_synthetase | BGC0001117 | Himastatin\_biosynthetic\_gene\_cluster | 37 | 24.2776886035 | 316 | 3.9e-85 |
| CAE02630.1 | surfactin\_synthetase\_A | BGC0000433 | Surfactin\_biosynthetic\_gene\_cluster | 36 | 23.6757624398 | 316 | 3.9e-85 |
| AGM14934.1 | xantholysin\_synthetase\_C | BGC0000463 | Xantholysin\_biosynthetic\_gene\_cluster | 35 | 26.3242375602 | 316 | 3.9e-85 |
| CAG23968.1 | polyketide\_synthase\_type\_I | BGC0000181 | Macrolactin\_biosynthetic\_gene\_cluster | 37 | 20.9069020867 | 316 | 3.9e-85 |
| ABL74936.1 | TlmX | BGC0001048 | Tallysomycin\_biosynthetic\_gene\_cluster | 36 | 23.9967897271 | 315 | 5.1e-85 |
| AAP92491.1 | nonribosomal\_peptide\_synthetase | BGC0000458 | Viomycin\_biosynthetic\_gene\_cluster | 36 | 25.481540931 | 315 | 5.1e-85 |
| CBZ42146.1 | putative\_non-ribosomal\_peptide\_synthetase | BGC0001117 | Himastatin\_biosynthetic\_gene\_cluster | 38 | 23.1942215088 | 315 | 6.7e-85 |
| EFL06865.1 | hypothetical\_protein | BGC0000300 | Amychelin\_biosynthetic\_gene\_cluster | 36 | 23.3948635634 | 315 | 6.7e-85 |
| AED90003.1 | non-ribosomal\_peptide\_synthetase\_ThaB | BGC0000443 | Thanamycin\_biosynthetic\_gene\_cluster | 35 | 24.8394863563 | 315 | 6.7e-85 |
| BAC76471.1 | type\_I\_polyketide\_synthase\_LkcF | BGC0001100 | Lankacidin\_biosynthetic\_gene\_cluster | 36 | 22.9133226324 | 314 | 1.1e-84 |
| AIJ04686.1 | polyketide\_synthase | BGC0001383 | Macrolactin\_biosynthetic\_gene\_cluster | 37 | 20.9069020867 | 314 | 1.5e-84 |
| AHZ34232.1 | CifA | BGC0000323 | Cichofactin\_biosynthetic\_gene\_cluster | 38 | 24.4783306581 | 314 | 1.5e-84 |
| ALK27916.1 | non-ribosomal\_peptide\_synthase | BGC0001233 | Feglymycin\_biosynthetic\_gene\_cluster | 36 | 23.7560192616 | 313 | 1.9e-84 |
| AEA30273.1 | peptide\_synthetase | BGC0000429 | Skyllamycin\_biosynthetic\_gene\_cluster | 38 | 23.113964687 | 313 | 1.9e-84 |
| EWS95122.1 | hypothetical\_protein | BGC0000306 | Arylomycin\_biosynthetic\_gene\_cluster | 36 | 23.9967897271 | 313 | 1.9e-84 |
| AGN74893.1 | nonribosomal\_peptide\_synthetase/polyketide\_synthase\_hybrid\_protein | BGC0000459 | Griseoviridin\_/\_viridogrisein\_biosynthetic\_gene\_cluster | 33 | 27.5682182986 | 313 | 1.9e-84 |
| BAP05589.1 | CalA | BGC0000967 | Calyculin\_biosynthetic\_gene\_cluster | 37 | 21.5088282504 | 313 | 1.9e-84 |
| sln9 | nonribosomal\_peptide\_synthetase | BGC0001230 | Salinamides\_biosynthetic\_gene\_cluster | 37 | 22.9935794543 | 313 | 1.9e-84 |
| ACY06292.1 | modular\_polyketide\_synthase | BGC0001042 | Sanglifehrin\_A\_biosynthetic\_gene\_cluster | 31 | 34.1492776886 | 313 | 1.9e-84 |
| AAO56328.1 | non-ribosomal\_peptide\_synthetase\_SyfA | BGC0000435 | Syringafactin\_biosynthetic\_gene\_cluster | 37 | 24.2776886035 | 313 | 2.5e-84 |
| BAH22763.1 | nonribosomal\_peptide\_synthetase | BGC0001018 | Micropeptin\_biosynthetic\_gene\_cluster | 33 | 25.2006420546 | 313 | 2.5e-84 |
| BAP16697.1 | nonribosomal\_peptide\_synthetase | BGC0000376 | JBIR-34,\_JBIR-35\_biosynthetic\_gene\_cluster | 37 | 22.9935794543 | 313 | 2.5e-84 |
| AGN74876.1 | nonribosomal\_peptide\_synthetase | BGC0000459 | Griseoviridin\_/\_viridogrisein\_biosynthetic\_gene\_cluster | 35 | 25.1203852327 | 313 | 2.5e-84 |
| ABI91468.1 | beta-ketoacyl\_synthase | BGC0001094 | Enacyloxin\_biosynthetic\_gene\_cluster | 39 | 20.5457463884 | 312 | 3.3e-84 |
| AGZ03650.1 | SevA | BGC0000426 | Sevadicin\_biosynthetic\_gene\_cluster | 33 | 24.4382022472 | 312 | 3.3e-84 |
| AFN27481.1 | pks\_BonB | BGC0000173 | Bongkrekic\_acid\_biosynthetic\_gene\_cluster | 36 | 21.227929374 | 312 | 4.3e-84 |
| AEH41793.1 | HrmO | BGC0000374 | Hormaomycin\_biosynthetic\_gene\_cluster | 37 | 23.9165329053 | 312 | 5.7e-84 |
| ALV82388.1 | CDA\_peptide\_synthetase\_III | BGC0001370 | Lipopeptide\_8D1-1\_/\_lipopeptide\_8D1-2\_biosynthetic\_gene\_cluster | 37 | 23.0337078652 | 312 | 5.7e-84 |
| ALK27914.1 | non-ribosomal\_peptide\_synthase | BGC0001233 | Feglymycin\_biosynthetic\_gene\_cluster | 37 | 23.3948635634 | 311 | 7.4e-84 |
| AAY89053.1 | polyketide\_synthase | BGC0001069 | Chivosazole\_biosynthetic\_gene\_cluster | 34 | 24.1974317817 | 311 | 7.4e-84 |
| AFX60341.1 | polyketide\_synthase | BGC0001032 | Oocydin\_A\_biosynthetic\_gene\_cluster | 32 | 26.7656500803 | 311 | 7.4e-84 |
| ABR67744.1 | CmnA | BGC0000316 | Capreomycin\_biosynthetic\_gene\_cluster | 35 | 25.3611556982 | 311 | 9.6e-84 |
| BAP05595.1 | CalG | BGC0000967 | Calyculin\_biosynthetic\_gene\_cluster | 34 | 26.3242375602 | 311 | 9.6e-84 |
| CAG23983.1 | polyketide\_synthase\_type\_I | BGC0000176 | Difficidin\_biosynthetic\_gene\_cluster | 37 | 20.6661316212 | 311 | 9.6e-84 |
| AHZ20773.1 | non-ribosomal\_peptide\_synthase | BGC0000369 | Hassallidins\_biosynthetic\_gene\_cluster | 35 | 24.077046549 | 311 | 1.3e-83 |
| AGN74892.1 | nonribosomal\_peptide\_synthetase/polyketide\_synthase\_hybrid\_protein | BGC0000459 | Griseoviridin\_/\_viridogrisein\_biosynthetic\_gene\_cluster | 33 | 27.1669341894 | 310 | 1.6e-83 |
| ACZ55946.1 | non-ribosomal\_peptide\_synthetase | BGC0000302 | Anabaenopeptin\_biosynthetic\_gene\_cluster | 33 | 25.1203852327 | 310 | 2.1e-83 |
| BAP27942.1 | nonribosomal\_peptide\_synthetase | BGC0001085 | Teleocidin\_B\_biosynthetic\_gene\_cluster | 37 | 24.2375601926 | 310 | 2.1e-83 |
| AHD05617.1 | putative\_non-ribosomal\_peptide\_ligase\_domain\_protein | BGC0001033 | Paenilamicin\_biosynthetic\_gene\_cluster | 34 | 24.3579454254 | 310 | 2.1e-83 |
| ABS90478.1 | PKS | BGC0001106 | Oxazolomycin\_biosynthetic\_gene\_cluster | 35 | 28.0497592295 | 310 | 2.1e-83 |
| AEH59099.1 | amino\_acid\_adenylation\_domain-containing\_protein/NRPS | BGC0000385 | Lysobactin\_biosynthetic\_gene\_cluster | 37 | 23.3547351525 | 309 | 2.8e-83 |
| AFO85453.1 | non-ribosomal\_peptide\_synthetase | BGC0000391 | Methylpendolmycin/pendolmycin\_biosynthetic\_gene\_cluster | 37 | 23.7560192616 | 309 | 2.8e-83 |
| AAK81825.1 | peptide\_synthetase | BGC0000326 | Complestatin\_biosynthetic\_gene\_cluster | 36 | 23.3948635634 | 309 | 2.8e-83 |
| AGS77307.1 | NRPS\_modules\_1-2 | BGC0001178 | UK-68,597\_biosynthetic\_gene\_cluster | 35 | 26.3242375602 | 309 | 2.8e-83 |
| CAL69890.1 | RhiC\_protein | BGC0001112 | Rhizoxins\_biosynthetic\_gene\_cluster | 38 | 20.6260032103 | 309 | 2.8e-83 |
| CAM02313.1 | putative\_non-ribosomal\_peptide\_synthetase | BGC0000349 | Erythrochelin\_biosynthetic\_gene\_cluster | 37 | 23.8362760835 | 309 | 3.7e-83 |
| AAO56329.1 | non-ribosomal\_peptide\_synthetase\_SyfB | BGC0000435 | Syringafactin\_biosynthetic\_gene\_cluster | 34 | 26.3643659711 | 309 | 3.7e-83 |
| CAB38518.1 | CDA\_peptide\_synthetase\_I | BGC0000315 | Calcium-dependent\_antibiotic\_biosynthetic\_gene\_cluster | 35 | 25.3210272873 | 309 | 3.7e-83 |
| AFJ23825.1 | WLIP\_synthetase\_B | BGC0000462\_c2 | WLIP\_biosynthetic\_gene\_cluster | 34 | 25.7223113965 | 309 | 4.8e-83 |
| CAD55498.1 | CDA\_peptide\_synthetase\_III | BGC0000315 | Calcium-dependent\_antibiotic\_biosynthetic\_gene\_cluster | 37 | 23.7961476726 | 308 | 6.3e-83 |
| AAK81826.1 | peptide\_synthetase | BGC0000326 | Complestatin\_biosynthetic\_gene\_cluster | 36 | 23.8362760835 | 308 | 6.3e-83 |
| AAZ03551.1 | McnB | BGC0000332 | Cyanopeptolin\_biosynthetic\_gene\_cluster | 33 | 25.1605136437 | 308 | 8.2e-83 |
| ADN68480.1 | SorE | BGC0000184 | Sorangicin\_biosynthetic\_gene\_cluster | 38 | 21.6693418941 | 308 | 8.2e-83 |
| AGE11898.1 | nonribosomal\_peptide\_synthetase | BGC0000366 | Gobichelin\_biosynthetic\_gene\_cluster | 38 | 22.7929373997 | 308 | 8.2e-83 |
| CAG29031.1 | nonribosomal\_peptide\_synthetase\_(modules\_1\_and\_2) | BGC0001023 | Myxochromide\_biosynthetic\_gene\_cluster | 34 | 23.4349919743 | 308 | 8.2e-83 |
| CAQ71828.1 | non\_ribosomal\_peptide\_synthase,\_antibiotic\_synthesis;\_contains\_3\_condensation\_domains,\_2\_AMP-acid\_ligases\_II\_domains,\_2\_PP-binding,\_Phosphopantetheine\_attachment\_site | BGC0001189 | Taiwachelin\_biosynthetic\_gene\_cluster | 36 | 24.3579454254 | 308 | 8.2e-83 |
| AAM80537.1 | StaC | BGC0000290 | A47934\_biosynthetic\_gene\_cluster | 35 | 23.1942215088 | 308 | 8.2e-83 |
| ACY01402.1 | AT-less\_polyketide\_synthase | BGC0000083 | Lactimidomycin\_biosynthetic\_gene\_cluster | 35 | 23.6757624398 | 307 | 1.1e-82 |
| ABK32260.1 | AmbF | BGC0000014 | Ambruticin\_biosynthetic\_gene\_cluster | 36 | 22.9534510433 | 307 | 1.1e-82 |
| AOA33121.1 | nonribosomal\_peptide\_synthetase | BGC0001346 | Bananamides\_biosynthetic\_gene\_cluster | 35 | 23.9566613162 | 307 | 1.4e-82 |
| ABG94125.1 | non-ribosomal\_peptide\_synthetase | BGC0000417 | Rhodochelin\_biosynthetic\_gene\_cluster | 35 | 23.8362760835 | 307 | 1.4e-82 |
| AAY91419.3 | non-ribosomal\_peptide\_synthetase\_OfaA | BGC0000399 | Orfamide\_biosynthetic\_gene\_cluster | 36 | 24.2375601926 | 307 | 1.4e-82 |
| AEI58865.1 | peptide\_synthetase | BGC0000455 | Vancomycin\_biosynthetic\_gene\_cluster | 34 | 25.4012841091 | 307 | 1.4e-82 |
| EFE73312.1 | nonribosomal\_peptide\_synthetase | BGC0000431 | Stenothricin\_biosynthetic\_gene\_cluster | 38 | 24.2375601926 | 307 | 1.4e-82 |
| CAL69891.1 | RhiD\_protein | BGC0001112 | Rhizoxins\_biosynthetic\_gene\_cluster | 35 | 21.6292134831 | 307 | 1.4e-82 |
| ABS74179.1 | BmyC | BGC0001090 | Bacillomycin\_biosynthetic\_gene\_cluster | 34 | 23.8764044944 | 307 | 1.8e-82 |
| AID65224.1 | nonribosomal\_peptide\_synthetase | BGC0000335 | Cystomanamides\_biosynthetic\_gene\_cluster | 35 | 25.2808988764 | 307 | 1.8e-82 |
| CDG17982.1 | Non-ribosomal\_peptide\_synthetase | BGC0000464 | Xenoamicins\_biosynthetic\_gene\_cluster | 33 | 24.7191011236 | 307 | 1.8e-82 |
| WP\_052390334.1 | non-ribosomal\_peptide\_synthetase | BGC0001308\_c2 | Simocyclinone\_D9\_/\_simocyclinone\_D10\_/\_simocyclinone\_D11 | 35 | 23.9967897271 | 307 | 1.8e-82 |
| AIJ04680.1 | polyketide\_synthase | BGC0001383 | Macrolactin\_biosynthetic\_gene\_cluster | 32 | 25.0802568218 | 306 | 2.4e-82 |
| AJM89734.1 | PmxB | BGC0001192 | Colistin\_biosynthetic\_gene\_cluster | 34 | 23.9566613162 | 306 | 2.4e-82 |
| CAD17793.1 | probable\_non\_ribosomal\_peptide\_synthetase\_protein | BGC0001363 | Ralsolamycin\_biosynthetic\_gene\_cluster | 34 | 24.7592295345 | 306 | 2.4e-82 |
| BAB69700.1 | iturin\_A\_synthetase\_C | BGC0001098 | Iturin\_biosynthetic\_gene\_cluster | 33 | 24.1171749599 | 306 | 3.1e-82 |
| AAK81824.1 | peptide\_synthetase | BGC0000326 | Complestatin\_biosynthetic\_gene\_cluster | 35 | 24.518459069 | 306 | 3.1e-82 |
| ADH01487.1 | polyketide\_synthase | BGC0001096 | FR901464\_biosynthetic\_gene\_cluster | 37 | 21.5489566613 | 305 | 4.1e-82 |
| AAM80538.1 | StaB | BGC0000290 | A47934\_biosynthetic\_gene\_cluster | 35 | 23.595505618 | 305 | 4.1e-82 |
| KPN90369.1 | NunE | BGC0001416 | Nunapeptin\_/\_nunamycin\_biosynthetic\_gene\_cluster | 35 | 24.7592295345 | 305 | 4.1e-82 |
| AAY93355.1 | non-ribosomal\_peptide\_synthetase\_PvdJ | BGC0000413 | Pyoverdine\_biosynthetic\_gene\_cluster | 35 | 24.5585874799 | 305 | 5.3e-82 |
| CAA11794.1 | PCZA363.3 | BGC0000322 | Chloroeremomycin\_biosynthetic\_gene\_cluster | 35 | 24.5585874799 | 305 | 5.3e-82 |
| ADG27358.1 | peptide\_synthetase | BGC0000296 | Actinomycin\_biosynthetic\_gene\_cluster | 36 | 23.6356340289 | 305 | 5.3e-82 |
| ABM63528.1 | BryC | BGC0000174 | Bryostatin\_biosynthetic\_gene\_cluster | 37 | 21.9101123596 | 305 | 5.3e-82 |
| AFX60340.1 | polyketide\_synthase | BGC0001032 | Oocydin\_A\_biosynthetic\_gene\_cluster | 35 | 22.1107544141 | 305 | 6.9e-82 |
| AFX60317.1 | polyketide\_synthase | BGC0001031 | Oocydin\_A\_biosynthetic\_gene\_cluster | 35 | 21.9903691814 | 305 | 6.9e-82 |
| AFK83819.1 | PtzC | BGC0001107 | Patellazole\_biosynthetic\_gene\_cluster | 34 | 24.4783306581 | 305 | 6.9e-82 |
| ALE27513.1 | putative\_polyketide\_synthase | BGC0001292 | A33853\_biosynthetic\_gene\_cluster | 29 | 36.9181380417 | 305 | 6.9e-82 |
| AAY93356.2 | non-ribosomal\_peptide\_synthetase\_PvdI | BGC0000413 | Pyoverdine\_biosynthetic\_gene\_cluster | 35 | 24.4382022472 | 305 | 6.9e-82 |
| EWS95124.1 | hypothetical\_protein | BGC0000306 | Arylomycin\_biosynthetic\_gene\_cluster | 37 | 22.8731942215 | 305 | 6.9e-82 |
| CAG15011.1 | peptide\_synthetase,\_module\_4-6 | BGC0000441 | Teicoplanin\_biosynthetic\_gene\_cluster | 37 | 23.8764044944 | 304 | 9e-82 |
| CAY48789.1 | putative\_non-ribosomal\_peptide\_synthetase | BGC0001312 | Viscosin\_biosynthetic\_gene\_cluster | 36 | 23.3146067416 | 304 | 9e-82 |
| ABD65957.1 | nonribosomal\_peptide\_synthetase | BGC0000341 | Enduracidin\_biosynthetic\_gene\_cluster | 36 | 23.7158908507 | 304 | 9e-82 |
| CAE14178.1 |  | BGC0001180 | Isopropylstilbene\_biosynthetic\_gene\_cluster | 35 | 21.0674157303 | 304 | 1.2e-81 |
| CBL93730.1 | NRPS | BGC0000360 | GE81112\_biosynthetic\_gene\_cluster | 36 | 23.9165329053 | 304 | 1.2e-81 |
| ACA97577.1 | PmxB | BGC0000408 | Polymyxin\_biosynthetic\_gene\_cluster | 34 | 23.5553772071 | 304 | 1.2e-81 |
| CAF05648.1 | TubC\_protein | BGC0001053 | Tubulysin\_biosynthetic\_gene\_cluster | 36 | 23.2343499197 | 304 | 1.2e-81 |
| ACM79810.1 | ZmaO | BGC0001059 | Zwittermycin\_A\_biosynthetic\_gene\_cluster | 32 | 24.3178170144 | 304 | 1.2e-81 |
| CAQ34921.1 | nonribosomal\_peptide\_synthetase | BGC0000986 | Dkxanthene\_biosynthetic\_gene\_cluster | 35 | 24.8796147673 | 304 | 1.5e-81 |
| AJV88375.1 | MfnC | BGC0001214 | Marformycins\_biosynthetic\_gene\_cluster | 36 | 24.3178170144 | 303 | 2e-81 |
| AEG64698.1 | LpmD | BGC0000379 | Laspartomycin\_biosynthetic\_gene\_cluster | 36 | 24.036918138 | 303 | 2.6e-81 |
| AHB82056.1 | non\_ribosomal\_peptide\_synthetase | BGC0001019 | Microsclerodermins\_biosynthetic\_gene\_cluster | 37 | 24.9197431782 | 302 | 4.5e-81 |
| AIE77057.1 | peptide\_synthetase | BGC0000418 | Ristocetin\_biosynthetic\_gene\_cluster | 36 | 23.8764044944 | 302 | 4.5e-81 |
| AJW76710.1 | DsaH | BGC0001196 | Desotamide\_biosynthetic\_gene\_cluster | 33 | 23.9967897271 | 302 | 4.5e-81 |
| AAG02364.1 | peptide\_synthetase\_NRPS2-1 | BGC0000963 | Bleomycin\_biosynthetic\_gene\_cluster | 34 | 25.2808988764 | 302 | 4.5e-81 |
| AHD05620.1 | putative\_polyketide\_synthase\_subunit | BGC0001033 | Paenilamicin\_biosynthetic\_gene\_cluster | 31 | 26.8459069021 | 302 | 5.9e-81 |
| CAE53351.1 | non-ribosomal\_peptide\_synthetase | BGC0000440 | Teicoplanin\_biosynthetic\_gene\_cluster | 35 | 23.3146067416 | 302 | 5.9e-81 |
| AGC09528.1 | NRPS | BGC0001183 | Lobophorin\_biosynthetic\_gene\_cluster | 39 | 20.8266452648 | 302 | 5.9e-81 |
| CAG15010.1 | peptide\_synthetase,\_module\_3 | BGC0000441 | Teicoplanin\_biosynthetic\_gene\_cluster | 35 | 23.3146067416 | 301 | 7.6e-81 |
| ACU71638.1 | amino\_acid\_adenylation\_domain\_protein | BGC0001154 | Cacibiocin\_biosynthetic\_gene\_cluster | 36 | 23.2744783307 | 301 | 7.6e-81 |
| AEZ51517.1 | PmxB | BGC0001153 | Polymyxin\_biosynthetic\_gene\_cluster | 34 | 23.5553772071 | 301 | 7.6e-81 |
| AEC14346.1 | nonribosomal\_peptide\_synthetase | BGC0000377 | Koranimine\_biosynthetic\_gene\_cluster | 32 | 23.2343499197 | 301 | 1e-80 |
| AGS77309.1 | NRPS\_modules\_4-6 | BGC0001178 | UK-68,597\_biosynthetic\_gene\_cluster | 36 | 23.6757624398 | 301 | 1e-80 |
| AEW31022.1 | plipastatin\_synthetase | BGC0000407 | Plipastatin\_biosynthetic\_gene\_cluster | 35 | 25.0401284109 | 301 | 1e-80 |
| KFH48607.1 | N-like\_protein | BGC0000317 | Cephalosporin\_biosynthetic\_gene\_cluster | 34 | 24.5987158909 | 301 | 1e-80 |
| CCJ67638.1 | TaaC | BGC0000447 | Tolaasin\_biosynthetic\_gene\_cluster | 33 | 24.4382022472 | 301 | 1e-80 |
| ABS74208.1 | FenB | BGC0001095 | Fengycin\_biosynthetic\_gene\_cluster | 34 | 23.8362760835 | 300 | 1.3e-80 |
| BAP05591.1 | CalC | BGC0000967 | Calyculin\_biosynthetic\_gene\_cluster | 37 | 21.6693418941 | 300 | 1.3e-80 |
| AAS47562.1 | mixed\_type\_I\_polyketide\_synthase\_-\_peptide\_synthetase | BGC0001108 | Pederin\_biosynthetic\_gene\_cluster | 36 | 22.4317817014 | 300 | 1.7e-80 |
| AEC04362.1 | polyketide\_synthase | BGC0000178 | Elansolid\_biosynthetic\_gene\_cluster | 36 | 21.227929374 | 300 | 1.7e-80 |
| AFH75328.1 | nonribosomal\_peptide\_synthetase | BGC0000398 | Orfamide\_biosynthetic\_gene\_cluster | 35 | 23.7158908507 | 300 | 2.2e-80 |
| AID65225.1 | nonribosomal\_peptide\_synthetase | BGC0000335 | Cystomanamides\_biosynthetic\_gene\_cluster | 33 | 27.0866773676 | 300 | 2.2e-80 |
| AJW76711.1 | DsaI | BGC0001196 | Desotamide\_biosynthetic\_gene\_cluster | 37 | 23.3146067416 | 300 | 2.2e-80 |
| CDG12865.1 | non-ribosomal\_peptide\_synthetase | BGC0001415 | Althiomycin\_biosynthetic\_gene\_cluster | 33 | 23.2343499197 | 299 | 2.9e-80 |
| EYT83433.1 | hypothetical\_protein | BGC0001213 | Nataxazole\_biosynthetic\_gene\_cluster | 28 | 36.5971107544 | 299 | 3.8e-80 |
| CAE53352.1 | non-ribosomal\_peptide\_synthetase | BGC0000440 | Teicoplanin\_biosynthetic\_gene\_cluster | 36 | 23.8764044944 | 299 | 3.8e-80 |
| AAN32981.1 | BarG | BGC0000962 | Barbamide\_biosynthetic\_gene\_cluster | 33 | 23.9967897271 | 299 | 5e-80 |
| ADH01488.1 | polyketide\_synthase | BGC0001096 | FR901464\_biosynthetic\_gene\_cluster | 34 | 23.9566613162 | 299 | 5e-80 |
| AHZ20784.1 | non-ribosomal\_peptide\_synthase | BGC0000369 | Hassallidins\_biosynthetic\_gene\_cluster | 32 | 24.4382022472 | 299 | 5e-80 |
| ABV56585.1 | KtzE | BGC0000378 | Kutznerides\_biosynthetic\_gene\_cluster | 35 | 23.7158908507 | 299 | 5e-80 |
| CAB38517.1 | CDA\_peptide\_synthetase\_II | BGC0000315 | Calcium-dependent\_antibiotic\_biosynthetic\_gene\_cluster | 35 | 24.6388443018 | 299 | 5e-80 |
| CAE02633.1 | surfactin\_synthetase\_C | BGC0000433 | Surfactin\_biosynthetic\_gene\_cluster | 34 | 23.595505618 | 298 | 8.5e-80 |
| CBJ79915.1 | putative\_Phenylalanine\_racemase\_(ATP-hydrolyzing) | BGC0001133 | Taxlllaid\_biosynthetic\_gene\_cluster | 36 | 23.595505618 | 298 | 8.5e-80 |
| ADA69239.2 | trans-AT\_hybrid\_polyketide\_synthase-NRPS | BGC0001071 | Nosperin\_biosynthetic\_gene\_cluster | 32 | 24.6388443018 | 298 | 8.5e-80 |
| CAN89656.1 | putative\_non-ribosomal\_peptide\_synthetase | BGC0001070 | Kirromycin\_biosynthetic\_gene\_cluster | 35 | 24.8796147673 | 297 | 1.1e-79 |
| CAQ71829.1 | non\_ribosomal\_peptide\_synthase,\_antibiotic\_synthesis;\_contains\_4\_condensation\_domains,\_3\_AMP-acid\_ligases\_II\_domains,\_3\_PP-binding,\_Phosphopantetheine\_attachment\_site\_and\_a\_putative\_thioesterase\_domain | BGC0001189 | Taiwachelin\_biosynthetic\_gene\_cluster | 35 | 23.5152487961 | 297 | 1.1e-79 |
| AAZ55898.1 | amino\_acid\_adenylation | BGC0000359 | Fuscachelin\_biosynthetic\_gene\_cluster | 35 | 23.7961476726 | 297 | 1.1e-79 |
| AEI58879.1 | peptide\_synthetase | BGC0000455 | Vancomycin\_biosynthetic\_gene\_cluster | 37 | 23.113964687 | 297 | 1.4e-79 |
| BAO84866.1 | putative\_non-ribosomal\_peptide\_synthetase | BGC0000414 | Quinocarcin\_biosynthetic\_gene\_cluster | 34 | 23.7961476726 | 297 | 1.4e-79 |
| BAC67535.1 | arthrofactin\_synthetase\_B | BGC0000305 | Arthrofactin\_biosynthetic\_gene\_cluster | 34 | 25.963081862 | 297 | 1.9e-79 |
| ADN68478.1 | SorC | BGC0000184 | Sorangicin\_biosynthetic\_gene\_cluster | 34 | 22.632423756 | 297 | 1.9e-79 |
| CAJ77696.1 | MPS2\_protein | BGC0000363 | Glycopeptidolipid\_biosynthetic\_gene\_cluster | 35 | 23.3547351525 | 297 | 1.9e-79 |
| AFX60318.1 | polyketide\_synthase | BGC0001031 | Oocydin\_A\_biosynthetic\_gene\_cluster | 33 | 23.1540930979 | 296 | 2.5e-79 |
| AAW03326.1 | CtaC | BGC0000982 | Cystothiazole\_A\_biosynthetic\_gene\_cluster | 33 | 24.3178170144 | 296 | 2.5e-79 |
| AOA33122.1 | nonribosomal\_peptide\_synthetase | BGC0001346 | Bananamides\_biosynthetic\_gene\_cluster | 35 | 23.8764044944 | 296 | 2.5e-79 |
| CBW45749.1 | hybrid\_PKS/NRPS\_(PKSI) | BGC0000952\_c3 | Pristinamycin\_biosynthetic\_gene\_cluster | 32 | 26.886035313 | 296 | 2.5e-79 |
| CAD15512.1 | probable\_polyketide\_synthase\_protein | BGC0001014 | Micacocidin\_biosynthetic\_gene\_cluster | 37 | 22.3113964687 | 296 | 3.2e-79 |
| BAP05593.1 | CalE | BGC0000967 | Calyculin\_biosynthetic\_gene\_cluster | 36 | 21.4686998395 | 296 | 3.2e-79 |
| ABC36785.1 | peptide\_synthetase,\_putative | BGC0000964 | Burkholdac\_biosynthetic\_gene\_cluster | 33 | 26.4446227929 | 296 | 3.2e-79 |
| AAY37653.1 | Amino\_acid\_adenylation | BGC0000437 | Syringomycin\_biosynthetic\_gene\_cluster | 33 | 24.036918138 | 296 | 3.2e-79 |
| ABP55169.1 | amino\_acid\_adenylation\_domain | BGC0000150 | Sporolide\_biosynthetic\_gene\_cluster | 33 | 24.1573033708 | 295 | 4.2e-79 |
| AAL15600.1 | SimH | BGC0000270 | Simocyclinone\_biosynthetic\_gene\_cluster | 35 | 24.5585874799 | 295 | 4.2e-79 |
| AAG34184.1 | SimH | BGC0000835 | Simocyclinone\_biosynthetic\_gene\_cluster | 35 | 24.5585874799 | 295 | 4.2e-79 |
| AAK06804.1 | putative\_peptide\_synthase\_SimD6 | BGC0001072 | Simocyclinone\_biosynthetic\_gene\_cluster | 35 | 24.5585874799 | 295 | 4.2e-79 |
| AIG79243.1 | Hypothetical\_protein | BGC0000419 | Ristomycin\_A\_biosynthetic\_gene\_cluster | 34 | 25.2407704655 | 295 | 5.5e-79 |
| AHH53508.1 | non-ribosomal\_peptide\_synthetase | BGC0000439 | Taromycin\_biosynthetic\_gene\_cluster | 38 | 23.2343499197 | 295 | 5.5e-79 |
| ABM63530.1 | BryD | BGC0000174 | Bryostatin\_biosynthetic\_gene\_cluster | 35 | 20.746388443 | 295 | 5.5e-79 |
| AAD44234.1 | PstB | BGC0000362 | Glycopeptidolipid\_biosynthetic\_gene\_cluster | 34 | 25.3210272873 | 295 | 5.5e-79 |
| CAJ77716.1 | Mps2\_protein | BGC0000364 | Glycopeptidolipid\_biosynthetic\_gene\_cluster | 35 | 23.3547351525 | 295 | 5.5e-79 |
| CAJ57411.1 | polyketide\_synthase\_type\_I | BGC0000176 | Difficidin\_biosynthetic\_gene\_cluster | 36 | 21.227929374 | 295 | 7.2e-79 |
| CAG23965.1 | polyketide\_synthase\_type\_I | BGC0000181 | Macrolactin\_biosynthetic\_gene\_cluster | 34 | 21.1476725522 | 295 | 7.2e-79 |
| CBW45647.1 | PI\_synthetase\_3\_and\_4 | BGC0000952 | Pristinamycin\_biosynthetic\_gene\_cluster | 35 | 24.6789727127 | 295 | 7.2e-79 |
| AGZ15459.1 | putative\_non-ribosomal\_peptide\_synthetase | BGC0001036 | Polyoxypeptin\_biosynthetic\_gene\_cluster | 35 | 25.0401284109 | 294 | 9.3e-79 |
| AAY93354.1 | non-ribosomal\_peptide\_synthetase\_PvdD | BGC0000413 | Pyoverdine\_biosynthetic\_gene\_cluster | 34 | 24.8394863563 | 294 | 9.3e-79 |
| ADH04679.1 | non-ribosomal\_peptide\_synthetase | BGC0001344 | Tubulysin\_biosynthetic\_gene\_cluster | 35 | 24.036918138 | 294 | 9.3e-79 |
| CDG17981.1 | Non-ribosomal\_peptide\_synthetase | BGC0000464 | Xenoamicins\_biosynthetic\_gene\_cluster | 32 | 26.8459069021 | 294 | 1.2e-78 |
| AIJ04685.1 | polyketide\_synthase | BGC0001383 | Macrolactin\_biosynthetic\_gene\_cluster | 34 | 20.9069020867 | 294 | 1.2e-78 |
| ACZ55943.1 | non-ribosomal\_peptide\_synthetase | BGC0000302 | Anabaenopeptin\_biosynthetic\_gene\_cluster | 33 | 23.8362760835 | 294 | 1.2e-78 |
| ALG65318.1 | Cal18 | BGC0001297 | WS9326\_biosynthetic\_gene\_cluster | 36 | 24.3178170144 | 294 | 1.2e-78 |
| BAF50721.1 | hybrid\_non\_ribosomal\_peptide\_synthetase-polyketide\_synthase | BGC0001116 | Virginiamycin\_biosynthetic\_gene\_cluster | 36 | 22.2311396469 | 294 | 1.2e-78 |
| CAD29797.1 | peptide\_synthetase | BGC0001015 | Microcystin\_biosynthetic\_gene\_cluster | 34 | 24.3178170144 | 294 | 1.2e-78 |
| CBJ79916.1 | putative\_Ornithine\_racemase | BGC0001133 | Taxlllaid\_biosynthetic\_gene\_cluster | 34 | 24.077046549 | 294 | 1.2e-78 |
| CAG23969.1 | polyketide\_synthase\_type\_I | BGC0000181 | Macrolactin\_biosynthetic\_gene\_cluster | 33 | 20.9069020867 | 294 | 1.6e-78 |
| AAK81827.1 | peptide\_synthetase | BGC0000326 | Complestatin\_biosynthetic\_gene\_cluster | 35 | 23.2744783307 | 294 | 1.6e-78 |
| AEC14348.1 | nonribosomal\_peptide\_synthetase | BGC0000377 | Koranimine\_biosynthetic\_gene\_cluster | 35 | 23.1540930979 | 294 | 1.6e-78 |
| CAC48360.1 | peptide\_synthetase | BGC0000311 | Balhimycin\_biosynthetic\_gene\_cluster | 36 | 24.3178170144 | 294 | 1.6e-78 |
| AAT01807.1 | non-ribosomal\_peptide\_synthetase | BGC0000365 | Glycopeptidolipid\_biosynthetic\_gene\_cluster | 34 | 23.3948635634 | 293 | 2.1e-78 |
| AFJ23826.1 | WLIP\_synthetase\_C | BGC0000462\_c2 | WLIP\_biosynthetic\_gene\_cluster | 34 | 24.2375601926 | 293 | 2.1e-78 |
| AAG02355.1 | peptide\_synthetase\_NRPS9-8 | BGC0000963 | Bleomycin\_biosynthetic\_gene\_cluster | 34 | 23.9566613162 | 293 | 2.1e-78 |
| AEW31015.1 | plipastatin\_synthetase | BGC0000407 | Plipastatin\_biosynthetic\_gene\_cluster | 34 | 23.3547351525 | 293 | 2.7e-78 |
| AJV88377.1 | MfnE | BGC0001214 | Marformycins\_biosynthetic\_gene\_cluster | 34 | 24.6388443018 | 293 | 2.7e-78 |
| CAN89638.1 | putative\_non-ribosomal\_peptide\_synthetase | BGC0001070 | Kirromycin\_biosynthetic\_gene\_cluster | 35 | 23.3547351525 | 293 | 2.7e-78 |
| AHZ20781.1 | non-ribosomal\_peptide\_synthase | BGC0000369 | Hassallidins\_biosynthetic\_gene\_cluster | 33 | 23.5553772071 | 293 | 2.7e-78 |
| BAD83684.1 | PKSN\_polyketide\_synthase\_for\_alternapyrone\_biosynthesis | BGC0000012 | Alternapyrone\_biosynthetic\_gene\_cluster | 33 | 21.227929374 | 293 | 2.7e-78 |
| AHF21229.1 | TriE | BGC0000449 | Tridecaptin\_biosynthetic\_gene\_cluster | 33 | 23.7158908507 | 293 | 2.7e-78 |
| ACG60773.1 | NRPS(C/A/PCP) | BGC0001058 | Zorbamycin\_biosynthetic\_gene\_cluster | 35 | 24.077046549 | 293 | 2.7e-78 |
| sln7 | nonribosomal\_peptide\_synthetase | BGC0001230 | Salinamides\_biosynthetic\_gene\_cluster | 34 | 23.8764044944 | 292 | 3.6e-78 |
| AKA54626.1 | NRPS | BGC0001216 | Splenocin\_biosynthetic\_gene\_cluster | 35 | 25.2407704655 | 292 | 3.6e-78 |
| ACM68690.1 | AerG1 | BGC0000298 | Aeruginosin\_biosynthetic\_gene\_cluster | 34 | 24.2776886035 | 292 | 3.6e-78 |
| AEG64696.1 | LpmB | BGC0000379 | Laspartomycin\_biosynthetic\_gene\_cluster | 37 | 23.7560192616 | 292 | 4.6e-78 |
| BAH33409.1 | putative\_non-ribosomal\_peptide\_synthetase | BGC0000371 | Heterobactin\_biosynthetic\_gene\_cluster | 37 | 20.1845906902 | 292 | 4.6e-78 |
| ABL74940.1 | TlmVI | BGC0001048 | Tallysomycin\_biosynthetic\_gene\_cluster | 34 | 24.6789727127 | 292 | 6.1e-78 |
| CBA63680.1 | nonribosomal\_peptide\_synthetase\_NRPS | BGC0000368 | Griseobactin\_biosynthetic\_gene\_cluster | 34 | 24.8796147673 | 292 | 6.1e-78 |
| CDG17986.1 | Non-ribosomal\_peptide\_synthetase | BGC0000464 | Xenoamicins\_biosynthetic\_gene\_cluster | 34 | 23.3948635634 | 292 | 6.1e-78 |
| CUI25745.1 | Nonribosomal\_peptide\_synthase | BGC0001354 | U-68204\_biosynthetic\_gene\_cluster | 33 | 24.4382022472 | 291 | 7.9e-78 |
| CAG29032.1 | nonribosomal\_peptide\_synthetase\_(modules\_3\_to\_6) | BGC0001023 | Myxochromide\_biosynthetic\_gene\_cluster | 33 | 23.9566613162 | 291 | 7.9e-78 |
| AKC91856.1 | nonribosomal\_peptide\_synthetase | BGC0001414 | Griselimycin\_biosynthetic\_gene\_cluster | 36 | 24.1171749599 | 291 | 7.9e-78 |
| CAJ46692.1 | non\_ribosomal\_peptide\_synthase | BGC0000969 | Chondramide\_biosynthetic\_gene\_cluster | 34 | 22.7929373997 | 291 | 7.9e-78 |
| WP\_010639240.1 | gramicidin\_synthetase | BGC0000958 | Antimycin\_biosynthetic\_gene\_cluster | 35 | 25.2407704655 | 291 | 1e-77 |
| ABD65956.1 | nonribosomal\_peptide\_synthetase | BGC0000341 | Enduracidin\_biosynthetic\_gene\_cluster | 34 | 23.9165329053 | 291 | 1e-77 |
| CUI25726.1 | Polyketide\_synthase | BGC0001355 | U-68204\_biosynthetic\_gene\_cluster | 34 | 21.6693418941 | 290 | 1.3e-77 |
| CAC48361.1 | peptide\_synthetase | BGC0000311 | Balhimycin\_biosynthetic\_gene\_cluster | 35 | 23.7158908507 | 290 | 1.3e-77 |
| ACZ55944.1 | non-ribosomal\_peptide\_synthetase | BGC0000302 | Anabaenopeptin\_biosynthetic\_gene\_cluster | 33 | 24.3178170144 | 290 | 1.3e-77 |
| ADH01484.1 | putative\_type-I\_PKS | BGC0001096 | FR901464\_biosynthetic\_gene\_cluster | 34 | 26.2439807384 | 290 | 1.8e-77 |
| AIC32692.1 | FR9C | BGC0001113 | Spliceostatin\_/\_FR901464\_biosynthetic\_gene\_cluster | 34 | 26.2439807384 | 290 | 1.8e-77 |
| AHY86403.1 | non\_ribosomal\_peptide\_synthetase | BGC0000329 | Corpeptin\_biosynthetic\_gene\_cluster | 36 | 22.8731942215 | 290 | 1.8e-77 |
| CUI25728.1 | Nonribosomal\_peptide\_synthase | BGC0001355 | U-68204\_biosynthetic\_gene\_cluster | 32 | 24.3980738363 | 290 | 2.3e-77 |
| ABS74205.1 | FenE | BGC0001095 | Fengycin\_biosynthetic\_gene\_cluster | 32 | 23.3547351525 | 289 | 3e-77 |
| CAE14171.1 |  | BGC0000383 | Luminmycin\_biosynthetic\_gene\_cluster | 34 | 23.8764044944 | 289 | 3.9e-77 |
| ABV56588.1 | KtzH | BGC0000378 | Kutznerides\_biosynthetic\_gene\_cluster | 33 | 23.9165329053 | 289 | 3.9e-77 |
| CAJ77695.1 | MPS1\_protein | BGC0000363 | Glycopeptidolipid\_biosynthetic\_gene\_cluster | 33 | 23.9967897271 | 289 | 5.1e-77 |
| BAP05596.1 | CalH | BGC0000967 | Calyculin\_biosynthetic\_gene\_cluster | 32 | 25.8025682183 | 289 | 5.1e-77 |
| AAD44233.1 | PstA | BGC0000362 | Glycopeptidolipid\_biosynthetic\_gene\_cluster | 34 | 24.5987158909 | 289 | 5.1e-77 |
| ABA70582.1 | alpha-aminoadypil-cysteinyl-valine\_synthetase | BGC0000404 | Penicillin\_biosynthetic\_gene\_cluster | 33 | 24.1974317817 | 288 | 8.7e-77 |
| ABS90472.1 | PKS | BGC0001106 | Oxazolomycin\_biosynthetic\_gene\_cluster | 32 | 24.2776886035 | 287 | 1.1e-76 |
| CAJ34367.1 | NRPS\_protein | BGC0000445 | Thiocoraline\_biosynthetic\_gene\_cluster | 35 | 24.3178170144 | 287 | 1.1e-76 |
| AAF19811.1 | MtaC | BGC0001024 | Myxothiazol\_biosynthetic\_gene\_cluster | 32 | 26.0032102729 | 287 | 1.1e-76 |
| ABV79988.1 | ApnD | BGC0000301 | Anabaenopeptin\_biosynthetic\_gene\_cluster | 31 | 24.1171749599 | 287 | 1.1e-76 |
| ALJ49920.1 | TtmI | BGC0001236 | Thiotetroamide\_biosynthetic\_gene\_cluster | 33 | 23.7961476726 | 287 | 1.5e-76 |
| CAD91221.1 | putative\_non-ribosomal\_peptide\_synthetase,\_module\_3 | BGC0000289 | A40926\_biosynthetic\_gene\_cluster | 34 | 24.2776886035 | 287 | 1.9e-76 |
| ABS74209.1 | FenA | BGC0001095 | Fengycin\_biosynthetic\_gene\_cluster | 34 | 23.4751203852 | 287 | 1.9e-76 |
| AAF63833.1 | PstD | BGC0000362 | Glycopeptidolipid\_biosynthetic\_gene\_cluster | 34 | 24.7993579454 | 287 | 1.9e-76 |
| ABI22131.1 | putative\_non-ribosomal\_peptide\_synthetase | BGC0000422 | Saframycin\_A\_biosynthetic\_gene\_cluster | 35 | 24.1974317817 | 287 | 1.9e-76 |
| ABR12615.1 | ACV\_synthetase | BGC0000405 | Penicillin\_biosynthetic\_gene\_cluster | 33 | 24.1974317817 | 287 | 1.9e-76 |
| CBW45648.1 | PI\_synthetase\_2 | BGC0000952 | Pristinamycin\_biosynthetic\_gene\_cluster | 35 | 24.4783306581 | 286 | 2.5e-76 |
| ABC34305.1 | peptide\_synthetase,\_putative | BGC0000961 | Bactobolin\_biosynthetic\_gene\_cluster | 36 | 21.6693418941 | 286 | 2.5e-76 |
| ABS90471.1 | PKS\_type\_I | BGC0001106 | Oxazolomycin\_biosynthetic\_gene\_cluster | 35 | 21.3483146067 | 285 | 4.3e-76 |
| AAX31559.1 | peptide\_synthetase\_3 | BGC0000336 | Daptomycin\_biosynthetic\_gene\_cluster | 35 | 23.9967897271 | 285 | 4.3e-76 |
| AEF16059.1 | non-ribosomal\_peptide\_synthetase | BGC0000953 | Amicetin\_biosynthetic\_gene\_cluster | 33 | 24.7191011236 | 285 | 5.7e-76 |
| CAN89662.1 | putative\_non-ribosomal\_peptide\_synthetase | BGC0001070 | Kirromycin\_biosynthetic\_gene\_cluster | 35 | 23.7560192616 | 285 | 7.4e-76 |
| CAJ34382.1 | NRPS\_protein | BGC0000445 | Thiocoraline\_biosynthetic\_gene\_cluster | 33 | 24.036918138 | 285 | 7.4e-76 |
| AAY89052.1 | polyketide\_synthase | BGC0001069 | Chivosazole\_biosynthetic\_gene\_cluster | 36 | 20.8667736758 | 285 | 7.4e-76 |
| AGS77308.1 | NRPS\_module\_3 | BGC0001178 | UK-68,597\_biosynthetic\_gene\_cluster | 33 | 23.9566613162 | 285 | 7.4e-76 |
| AGN11880.1 | TstC | BGC0001114 | Thailanstatin\_biosynthetic\_gene\_cluster | 33 | 26.3242375602 | 285 | 7.4e-76 |
| ACI12948.1 | PfaA | BGC0000861 | Eicosapentaenoic\_acid\_biosynthetic\_gene\_cluster | 28 | 29.253611557 | 285 | 7.4e-76 |
| ABL74939.1 | TlmVII | BGC0001048 | Tallysomycin\_biosynthetic\_gene\_cluster | 33 | 25.963081862 | 284 | 9.7e-76 |
| AGN74885.1 | nonribosomal\_peptide\_synthetase | BGC0000459 | Griseoviridin\_/\_viridogrisein\_biosynthetic\_gene\_cluster | 35 | 24.3579454254 | 284 | 1.6e-75 |
| AMK48228.1 | nonribosomal\_peptide\_synthetase | BGC0001351 | Cahuitamycins\_biosynthetic\_gene\_cluster | 36 | 24.1573033708 | 283 | 2.2e-75 |
| CAA11796.1 | PCZA363.5 | BGC0000322 | Chloroeremomycin\_biosynthetic\_gene\_cluster | 35 | 24.1171749599 | 283 | 2.2e-75 |
| CAM56771.1 |  | BGC0000354 | Friulimicin\_biosynthetic\_gene\_cluster | 34 | 23.4349919743 | 283 | 2.2e-75 |
| CAJ57410.1 | polyketide\_synthase\_type\_I | BGC0000176 | Difficidin\_biosynthetic\_gene\_cluster | 35 | 21.227929374 | 283 | 2.8e-75 |
| BAI63283.1 | putative\_non-ribosomal\_peptide\_synthetase | BGC0000434 | SW-163\_biosynthetic\_gene\_cluster | 34 | 23.8362760835 | 283 | 2.8e-75 |
| ALV86868.1 | Tlo22 | BGC0001406 | Telomycin\_biosynthetic\_gene\_cluster | 35 | 23.5553772071 | 282 | 3.7e-75 |
| CUI25701.1 | amino\_acid\_adenylation\_domain-containing\_protein\_potentially\_involved\_in\_thiotetronic\_acid\_synthesis | BGC0001352 | Thiotetronate\_Tu\_3010\_biosynthetic\_gene\_cluster | 32 | 23.7961476726 | 282 | 6.3e-75 |
| BAO84868.1 | putative\_non-ribosomal\_peptide\_synthetase | BGC0000414 | Quinocarcin\_biosynthetic\_gene\_cluster | 37 | 20.9871589085 | 282 | 6.3e-75 |
| CBW45748.1 | hybrid\_PKS/NRPS\_(PKSI) | BGC0000952\_c3 | Pristinamycin\_biosynthetic\_gene\_cluster | 35 | 24.1171749599 | 282 | 6.3e-75 |
| ACY06285.1 | non-ribosomal\_peptide\_synthetase | BGC0001042 | Sanglifehrin\_A\_biosynthetic\_gene\_cluster | 32 | 24.5987158909 | 282 | 6.3e-75 |
| AJW65406.1 | nonribosomal\_peptide\_synthetase | BGC0001195 | Nocardiopsin\_biosynthetic\_gene\_cluster | 33 | 24.5585874799 | 281 | 8.2e-75 |
| ACR13997.1 | modular\_polyketide\_synthase,\_type\_I\_PKS | BGC0000185 | Tartrolon\_biosynthetic\_gene\_cluster | 30 | 23.7560192616 | 281 | 1.1e-74 |
| AIE77058.1 | peptide\_synthetase\_module\_3 | BGC0000418 | Ristocetin\_biosynthetic\_gene\_cluster | 35 | 23.6757624398 | 280 | 1.4e-74 |
| ADN26248.1 | peptide\_synthetase | BGC0000951 | Pacidamycin\_biosynthetic\_gene\_cluster | 35 | 23.6356340289 | 280 | 1.4e-74 |
| AIS24862.1 | Dst18 | BGC0001147 | Distamycin\_biosynthetic\_gene\_cluster | 31 | 26.6452648475 | 280 | 1.4e-74 |
| ctg1\_orf19 |  | BGC0001013 | Meridamycin\_biosynthetic\_gene\_cluster | 33 | 25.3210272873 | 280 | 1.8e-74 |
| ABC87508.1 | NRPS\_for\_pipecolate\_incorporation | BGC0001011 | Meridamycin\_biosynthetic\_gene\_cluster | 33 | 25.3210272873 | 280 | 1.8e-74 |
| AGN74894.1 | nonribosomal\_peptide\_synthetase/polyketide\_synthase\_hybrid\_protein | BGC0000459 | Griseoviridin\_/\_viridogrisein\_biosynthetic\_gene\_cluster | 36 | 22.1107544141 | 280 | 2.4e-74 |
| ABC38737.1 | polyketide\_synthase | BGC0000964 | Burkholdac\_biosynthetic\_gene\_cluster | 34 | 22.3916532905 | 279 | 3.1e-74 |
| ABC94347.1 | vicibactin\_biosynthesis\_non-ribosomal\_peptide\_synthase\_protein | BGC0000457 | Vicibactin\_biosynthetic\_gene\_cluster | 33 | 24.3178170144 | 279 | 4.1e-74 |
| CAJ77715.1 | Mps1\_protein | BGC0000364 | Glycopeptidolipid\_biosynthetic\_gene\_cluster | 33 | 24.077046549 | 279 | 4.1e-74 |
| AIG79242.1 | Hypothetical\_protein | BGC0000419 | Ristomycin\_A\_biosynthetic\_gene\_cluster | 34 | 23.4751203852 | 279 | 4.1e-74 |
| AHB38515.1 | EpnG | BGC0000345 | Eponemycin\_biosynthetic\_gene\_cluster | 34 | 23.3146067416 | 279 | 4.1e-74 |
| AGI61661.1 | modular\_PKS | BGC0000095\_c5 | Micromonolactam\_biosynthetic\_gene\_cluster | 36 | 22.6725521669 | 279 | 5.3e-74 |
| AAT01806.1 | non-ribosomal\_peptide\_synthetase | BGC0000365 | Glycopeptidolipid\_biosynthetic\_gene\_cluster | 34 | 23.1540930979 | 279 | 5.3e-74 |
| BAF50711.1 | non\_ribosomal\_peptide\_synthetase\_for\_virginiamycin\_S | BGC0001116 | Virginiamycin\_biosynthetic\_gene\_cluster | 34 | 23.5152487961 | 278 | 6.9e-74 |
| AHD05621.1 | non-ribosomal\_peptide\_ligase\_domain\_protein | BGC0001033 | Paenilamicin\_biosynthetic\_gene\_cluster | 31 | 24.7592295345 | 278 | 6.9e-74 |
| ABM63537.1 | BryA | BGC0000174 | Bryostatin\_biosynthetic\_gene\_cluster | 33 | 25.0401284109 | 278 | 6.9e-74 |
| BAF50720.1 | hybrid\_non\_ribosomal\_peptide\_synthetase-polyketide\_synthase | BGC0001116 | Virginiamycin\_biosynthetic\_gene\_cluster | 30 | 28.3707865169 | 278 | 9.1e-74 |
| EFL06867.1 | predicted\_protein | BGC0000300 | Amychelin\_biosynthetic\_gene\_cluster | 35 | 23.5152487961 | 278 | 9.1e-74 |
| AIG79240.1 | Hypothetical\_protein | BGC0000419 | Ristomycin\_A\_biosynthetic\_gene\_cluster | 34 | 24.5585874799 | 277 | 1.5e-73 |
| CCJ67646.1 | JagB | BGC0001127 | Jagaricin\_biosynthetic\_gene\_cluster | 35 | 23.2744783307 | 277 | 1.5e-73 |
| AAZ23078.1 | peptide\_synthetase | BGC0000291 | A54145\_biosynthetic\_gene\_cluster | 35 | 23.8764044944 | 277 | 1.5e-73 |
| AAG02349.1 | peptide\_synthetase\_NRPS11-10 | BGC0000963 | Bleomycin\_biosynthetic\_gene\_cluster | 34 | 23.595505618 | 277 | 2e-73 |
| CAN89663.1 | putative\_non-ribosomal\_peptide\_synthetase | BGC0001070 | Kirromycin\_biosynthetic\_gene\_cluster | 35 | 23.7560192616 | 277 | 2e-73 |
| AJI44176.1 | nonribosomal\_peptide\_synthetase | BGC0001193 | Thiolutin\_biosynthetic\_gene\_cluster | 34 | 22.8330658106 | 276 | 2.6e-73 |
| AFR69334.1 | nonribosomal\_peptide\_synthetase\_SpiDE1 | BGC0001045 | Spiruchostatin\_biosynthetic\_gene\_cluster | 33 | 23.2744783307 | 276 | 3.4e-73 |
| AAM80536.1 | StaD | BGC0000290 | A47934\_biosynthetic\_gene\_cluster | 34 | 24.4783306581 | 276 | 3.4e-73 |
| AAY37650.1 | Amino\_acid\_adenylation | BGC0000437 | Syringomycin\_biosynthetic\_gene\_cluster | 33 | 24.2375601926 | 276 | 3.4e-73 |
| AHB82069.1 | non\_ribosomal\_peptide\_synthetase | BGC0001231 | Microsclerodermins\_biosynthetic\_gene\_cluster | 35 | 23.113964687 | 275 | 4.5e-73 |
| KPN90374.1 | NunB1 | BGC0001416 | Nunapeptin\_/\_nunamycin\_biosynthetic\_gene\_cluster | 32 | 24.6388443018 | 275 | 4.5e-73 |
| ABP57749.1 | DepE | BGC0000993 | FK228\_biosynthetic\_gene\_cluster | 33 | 22.7126805778 | 275 | 4.5e-73 |
| EDY47118.1 | N-(5-amino-5-carboxypentanoyl)-L-cysteinyl-D-\_valine\_synthase | BGC0000319 | Cephamycin\_C\_biosynthetic\_gene\_cluster | 32 | 23.8764044944 | 275 | 7.7e-73 |
| BAO84861.1 | putative\_non-ribosomal\_peptide\_synthetase | BGC0000414 | Quinocarcin\_biosynthetic\_gene\_cluster | 34 | 23.595505618 | 274 | 1e-72 |
| ABP57748.1 | DepD | BGC0000993 | FK228\_biosynthetic\_gene\_cluster | 35 | 24.036918138 | 274 | 1.3e-72 |
| CAD91220.1 | putative\_non-ribosomal\_peptide\_synthetase,\_modules\_1-2 | BGC0000289 | A40926\_biosynthetic\_gene\_cluster | 34 | 23.9165329053 | 274 | 1.3e-72 |
| ABC35027.1 | JamP | BGC0000961 | Bactobolin\_biosynthetic\_gene\_cluster | 35 | 22.4719101124 | 274 | 1.3e-72 |
| AGS77310.1 | NRPS\_module\_7 | BGC0001178 | UK-68,597\_biosynthetic\_gene\_cluster | 33 | 23.7560192616 | 274 | 1.7e-72 |
| ERG36783.1 | phenylalanine\_racemase | BGC0000299 | Alterochromides\_biosynthetic\_gene\_cluster | 32 | 24.8394863563 | 273 | 2.2e-72 |
| AIE77060.1 | peptide\_synthetase\_module\_7 | BGC0000418 | Ristocetin\_biosynthetic\_gene\_cluster | 34 | 23.6356340289 | 273 | 2.2e-72 |
| WP\_010369428.1 | phenylalanine\_racemase | BGC0000314 | Bromoalterochromides\_biosynthetic\_gene\_cluster | 32 | 24.8394863563 | 273 | 2.2e-72 |
| ABP57746.1 | DepB | BGC0000993 | FK228\_biosynthetic\_gene\_cluster | 34 | 22.4317817014 | 273 | 2.9e-72 |
| ABL74945.1 | TlmIV | BGC0001048 | Tallysomycin\_biosynthetic\_gene\_cluster | 31 | 24.518459069 | 273 | 2.9e-72 |
| CAC11137.1 | NikP1\_protein | BGC0000876 | Nikkomycin\_biosynthetic\_gene\_cluster | 33 | 24.3579454254 | 272 | 5e-72 |
| AAC68816.1 | FK506\_peptide\_synthetase | BGC0000353 | FK506\_biosynthetic\_gene\_cluster | 34 | 24.4783306581 | 272 | 5e-72 |
| AKP45399.1 | CysK | BGC0001413 | Cystobactamide\_biosynthetic\_gene\_cluster | 34 | 23.3146067416 | 272 | 5e-72 |
| CAD91211.1 | putative\_non-ribosomal\_peptide\_synthetase,\_module\_7 | BGC0000289 | A40926\_biosynthetic\_gene\_cluster | 33 | 25.4414125201 | 272 | 5e-72 |
| sln14 | nonribosomal\_peptide\_synthetase/polyketide\_synthase\_type\_I | BGC0001230 | Salinamides\_biosynthetic\_gene\_cluster | 35 | 23.8362760835 | 272 | 6.5e-72 |
| ABC34599.1 | polyketide\_synthase,\_putative | BGC0000186 | Thailandamide\_biosynthetic\_gene\_cluster | 33 | 22.632423756 | 272 | 6.5e-72 |
| CCJ67645.1 | JagA | BGC0001127 | Jagaricin\_biosynthetic\_gene\_cluster | 34 | 23.9967897271 | 272 | 6.5e-72 |
| sln6 | nonribosomal\_peptide\_synthetase | BGC0001230 | Salinamides\_biosynthetic\_gene\_cluster | 35 | 24.5987158909 | 271 | 8.5e-72 |
| CAJ34374.1 | NRPS\_protein | BGC0000445 | Thiocoraline\_biosynthetic\_gene\_cluster | 34 | 23.7158908507 | 271 | 1.1e-71 |
| CAB53322.1 | putative\_peptide\_synthetase | BGC0000325 | Coelichelin\_biosynthetic\_gene\_cluster | 34 | 23.2343499197 | 270 | 1.4e-71 |
| WP\_010369430.1 | Non-ribosomal\_peptide\_synthase | BGC0000314 | Bromoalterochromides\_biosynthetic\_gene\_cluster | 33 | 23.9566613162 | 270 | 1.4e-71 |
| CCM44337.1 | putative\_nonribosomal\_peptide\_synthetase | BGC0001056 | Zeamine\_biosynthetic\_gene\_cluster | 33 | 25.6420545746 | 270 | 1.9e-71 |
| ALV82446.1 | putative\_type\_I\_polyketide\_synthase | BGC0001382 | Svaricin\_biosynthetic\_gene\_cluster | 35 | 21.3483146067 | 270 | 1.9e-71 |
| AJV88376.1 | MfnD | BGC0001214 | Marformycins\_biosynthetic\_gene\_cluster | 34 | 24.3980738363 | 270 | 1.9e-71 |
| AEH41794.1 | HrmP | BGC0000374 | Hormaomycin\_biosynthetic\_gene\_cluster | 33 | 23.7158908507 | 270 | 2.5e-71 |
| CAJ45639.1 | vanchrobactin\_non\_ribosomal\_peptide\_synthetase | BGC0000454 | Vanchrobactin\_biosynthetic\_gene\_cluster | 34 | 23.0337078652 | 269 | 3.2e-71 |
| AEI58867.1 | peptide\_synthetase | BGC0000455 | Vancomycin\_biosynthetic\_gene\_cluster | 34 | 23.3948635634 | 269 | 3.2e-71 |
| AFR69332.1 | polyketide\_synthase\_SpiB | BGC0001045 | Spiruchostatin\_biosynthetic\_gene\_cluster | 34 | 20.9470304976 | 269 | 3.2e-71 |
| AEC04364.1 | polyketide\_synthase | BGC0000178 | Elansolid\_biosynthetic\_gene\_cluster | 35 | 21.227929374 | 269 | 3.2e-71 |
| CAJ76292.1 | putative\_non-ribosomal\_peptide\_synthase | BGC0000972 | Colibactin\_biosynthetic\_gene\_cluster | 34 | 23.9967897271 | 269 | 4.2e-71 |
| ABC34154.1 | thiotemplate\_mechanism\_natural\_product\_synthetase | BGC0000961 | Bactobolin\_biosynthetic\_gene\_cluster | 35 | 22.191011236 | 268 | 9.4e-71 |
| ACR12418.1 | modular\_polyketide\_synthase,\_type\_I\_PKS | BGC0000185 | Tartrolon\_biosynthetic\_gene\_cluster | 30 | 26.6853932584 | 267 | 1.2e-70 |
| CAG23959.2 | polyketide\_synthase | BGC0001089 | Bacillaene\_biosynthetic\_gene\_cluster | 31 | 24.8796147673 | 267 | 1.2e-70 |
| AET98916.1 | putative\_non-ribosomal\_peptide\_synthetase | BGC0000415 | Quinomycin\_biosynthetic\_gene\_cluster | 33 | 23.2343499197 | 267 | 2.1e-70 |
| BAE98162.1 | putative\_non-ribosomal\_peptide\_synthetase | BGC0000339 | Echinomycin\_biosynthetic\_gene\_cluster | 33 | 24.2375601926 | 267 | 2.1e-70 |
| CAC48362.1 | peptide\_synthetase | BGC0000311 | Balhimycin\_biosynthetic\_gene\_cluster | 34 | 23.2744783307 | 266 | 2.7e-70 |
| CAI94718.1 | putative\_CoA\_ligase | BGC0000141 | Rubradirin\_biosynthetic\_gene\_cluster | 33 | 23.9967897271 | 266 | 2.7e-70 |
| AAM12925.2 | MmpE | BGC0000182 | Mupirocin\_biosynthetic\_gene\_cluster | 33 | 21.8699839486 | 266 | 2.7e-70 |
| CBK62718.1 |  | BGC0001115 | Thiomarinol\_biosynthetic\_gene\_cluster | 31 | 25.963081862 | 266 | 2.7e-70 |
| ADL64235.1 | aureusimine\_non-ribosomal\_peptide\_synthetase | BGC0000308 | Aureusimine\_biosynthetic\_gene\_cluster | 29 | 23.5152487961 | 266 | 3.6e-70 |
| AAG02359.1 | peptide\_synthetase\_NRPS5-4-3 | BGC0000963 | Bleomycin\_biosynthetic\_gene\_cluster | 34 | 24.3579454254 | 265 | 4.6e-70 |
| AAT09804.1 | NocA | BGC0000395 | Nocardicin\_A\_biosynthetic\_gene\_cluster | 34 | 23.1540930979 | 265 | 6.1e-70 |
| EFE73308.1 | non-ribosomal\_peptide\_synthetase | BGC0000431 | Stenothricin\_biosynthetic\_gene\_cluster | 33 | 22.9935794543 | 265 | 7.9e-70 |
| BAH04173.1 | putative\_non-ribosomal\_peptide\_synthetase | BGC0000450 | Triostin\_A\_biosynthetic\_gene\_cluster | 33 | 23.5152487961 | 265 | 7.9e-70 |
| ACU36654.1 | amino\_acid\_adenylation\_domain\_protein | BGC0000392 | Mirubactin\_biosynthetic\_gene\_cluster | 34 | 23.7961476726 | 264 | 1e-69 |
| AFD30953.1 | CrmB | BGC0000966 | Caerulomycin\_A\_biosynthetic\_gene\_cluster | 32 | 24.2375601926 | 264 | 1e-69 |
| ABD14712.1 | CesB | BGC0000320 | Cereulide\_biosynthetic\_gene\_cluster | 31 | 23.5553772071 | 264 | 1.8e-69 |
| CAJ76289.1 | putative\_hybrid\_non-ribosomal\_peptide-polyketide\_synthetase | BGC0000972 | Colibactin\_biosynthetic\_gene\_cluster | 33 | 20.8266452648 | 263 | 3e-69 |
| CBL93718.1 | NRPS\_didomain\_PCP-C | BGC0000360 | GE81112\_biosynthetic\_gene\_cluster | 32 | 25.0401284109 | 262 | 6.7e-69 |
| AAN65224.1 | peptide\_synthetase-like\_protein | BGC0000832 | Clorobiocin\_biosynthetic\_gene\_cluster | 32 | 24.036918138 | 261 | 8.8e-69 |
| CAC17499.1 | putative\_non-ribosomal\_peptide\_synthase | BGC0000324 | Coelibactin\_biosynthetic\_gene\_cluster | 32 | 23.5553772071 | 261 | 1.1e-68 |
| CAJ76285.1 | putative\_polyketide\_synthase | BGC0000972 | Colibactin\_biosynthetic\_gene\_cluster | 36 | 21.0272873194 | 261 | 1.1e-68 |
| ALG65313.1 | Cal23 | BGC0001297 | WS9326\_biosynthetic\_gene\_cluster | 34 | 22.9935794543 | 261 | 1.1e-68 |
| AAM12934.1 | MmpF | BGC0000182 | Mupirocin\_biosynthetic\_gene\_cluster | 31 | 26.0032102729 | 260 | 1.5e-68 |
| CAE52339.1 | Polyketide\_non-ribosomal\_peptide\_synthase | BGC0001088 | Albicidin\_biosynthetic\_gene\_cluster | 33 | 23.4349919743 | 260 | 2e-68 |
| AEG64689.1 | LpmA | BGC0000379 | Laspartomycin\_biosynthetic\_gene\_cluster | 32 | 24.3178170144 | 260 | 2e-68 |
| ALJ49909.1 | TlmI | BGC0001237 | Thiolactomycin\_biosynthetic\_gene\_cluster | 33 | 21.5890850722 | 260 | 2.6e-68 |
| CAE53353.1 | non-ribosomal\_peptide\_synthetase | BGC0000440 | Teicoplanin\_biosynthetic\_gene\_cluster | 33 | 23.7158908507 | 259 | 3.3e-68 |
| CAG15012.1 | peptide\_synthetase,\_module\_7 | BGC0000441 | Teicoplanin\_biosynthetic\_gene\_cluster | 33 | 23.7158908507 | 259 | 3.3e-68 |
| CBW45740.1 | hybrid\_NRPS/PKS\_(PKSIV) | BGC0000952\_c3 | Pristinamycin\_biosynthetic\_gene\_cluster | 35 | 21.8298555377 | 259 | 4.4e-68 |
| CAD70194.1 | non-ribosomal\_peptide\_synthetase | BGC0001047 | Syringolin\_A\_biosynthetic\_gene\_cluster | 32 | 24.4382022472 | 259 | 5.7e-68 |
| AAZ23076.1 | peptide\_synthetase | BGC0000291 | A54145\_biosynthetic\_gene\_cluster | 33 | 23.6757624398 | 259 | 5.7e-68 |
| AME18003.1 | enediyne\_polyketie\_synthase | BGC0001378 | Tiancimycin\_biosynthetic\_gene\_cluster | 32 | 27.9695024077 | 258 | 7.4e-68 |
| AFK57219.1 | DidH | BGC0000985 | Didemnin\_biosynthetic\_gene\_cluster | 35 | 23.2744783307 | 258 | 9.7e-68 |
| AAG02356.1 | peptide\_synthetase\_NRPS7 | BGC0000963 | Bleomycin\_biosynthetic\_gene\_cluster | 34 | 24.518459069 | 257 | 1.3e-67 |
| ALA09359.1 | type\_I\_modular\_PKS | BGC0001303 | Lobosamide\_biosynthetic\_gene\_cluster | 35 | 21.1476725522 | 257 | 1.3e-67 |
| ALG65314.1 | Cal22 | BGC0001297 | WS9326\_biosynthetic\_gene\_cluster | 32 | 24.1171749599 | 257 | 1.7e-67 |
| ABP73646.1 | SalB | BGC0000145 | Salinosporamide\_A\_biosynthetic\_gene\_cluster | 32 | 23.3146067416 | 255 | 4.8e-67 |
| EOY45602.1 | non-ribosomal\_peptide\_synthetase | BGC0001168 | Livipeptin\_biosynthetic\_gene\_cluster | 33 | 22.3113964687 | 255 | 4.8e-67 |
| ABP53497.1 | amino\_acid\_adenylation\_domain | BGC0001041 | Salinosporamide\_biosynthetic\_gene\_cluster | 32 | 23.3146067416 | 255 | 4.8e-67 |
| EFG10344.1 | Non-ribosomal\_peptide\_synthetase | BGC0000373 | Holomycin\_biosynthetic\_gene\_cluster | 31 | 23.3948635634 | 255 | 4.8e-67 |
| ACG60776.1 | NRPS(AL/ACP/C/A/PCP/C/A) | BGC0001058 | Zorbamycin\_biosynthetic\_gene\_cluster | 34 | 24.036918138 | 255 | 6.3e-67 |
| AFK57212.1 | DidA | BGC0000985 | Didemnin\_biosynthetic\_gene\_cluster | 33 | 24.4783306581 | 255 | 6.3e-67 |
| CAE52334.1 | non-ribosomal\_peptide\_synthase | BGC0001088 | Albicidin\_biosynthetic\_gene\_cluster | 32 | 23.6757624398 | 254 | 1.1e-66 |
| AED90004.1 | non-ribosomal\_peptide\_synthetase\_ThaC1 | BGC0000443 | Thanamycin\_biosynthetic\_gene\_cluster | 32 | 22.9133226324 | 254 | 1.4e-66 |
| AAG05693.1 | AmbB | BGC0000287 | 2-amino-4-methoxy-trans-3-butenoic\_acid\_biosynthetic\_gene\_cluster | 33 | 23.1942215088 | 254 | 1.4e-66 |
| AKA59447.1 | non-ribosomal\_peptide\_synthetase | BGC0001203 | Clarexpoxcin\_biosynthetic\_gene\_cluster | 32 | 23.6356340289 | 253 | 1.8e-66 |
| WP\_010369425.1 | ornithine\_racemase | BGC0000314 | Bromoalterochromides\_biosynthetic\_gene\_cluster | 30 | 22.9935794543 | 253 | 2.4e-66 |
| ERG36782.1 | Ornithine\_racemase | BGC0000299 | Alterochromides\_biosynthetic\_gene\_cluster | 30 | 22.9935794543 | 253 | 2.4e-66 |
| CAJ18237.2 | non-ribosomal\_peptide\_synthetase\_B | BGC0000354 | Friulimicin\_biosynthetic\_gene\_cluster | 32 | 23.6757624398 | 253 | 3.1e-66 |
| sln8 | nonribosomal\_peptide\_synthetase | BGC0001230 | Salinamides\_biosynthetic\_gene\_cluster | 33 | 23.3547351525 | 253 | 3.1e-66 |
| AAG29780.1 | acyl-CoA\_synthetase | BGC0000833 | Coumermycin\_A1\_biosynthetic\_gene\_cluster | 33 | 24.036918138 | 252 | 4.1e-66 |
| CCM44336.1 | putative\_nonribosomal\_peptide\_synthetase | BGC0001056 | Zeamine\_biosynthetic\_gene\_cluster | 33 | 24.1171749599 | 252 | 5.3e-66 |
| CAJ96472.1 | Non-ribosomal\_peptide\_synthetase | BGC0000330 | Cupriachelin\_biosynthetic\_gene\_cluster | 32 | 24.1573033708 | 252 | 6.9e-66 |
| AKP45395.1 | CysG | BGC0001413 | Cystobactamide\_biosynthetic\_gene\_cluster | 33 | 23.2343499197 | 252 | 6.9e-66 |
| AAN85501.1 | nonribosomal\_peptide\_synthetase | BGC0001101 | Leinamycin\_biosynthetic\_gene\_cluster | 35 | 22.9133226324 | 252 | 6.9e-66 |
| ABD65960.1 | nonribosomal\_peptide\_synthetase | BGC0000341 | Enduracidin\_biosynthetic\_gene\_cluster | 32 | 23.3948635634 | 251 | 1.2e-65 |
| BAP16693.1 | nonribosomal\_peptide\_synthetase | BGC0000376 | JBIR-34,\_JBIR-35\_biosynthetic\_gene\_cluster | 31 | 23.9967897271 | 249 | 4.5e-65 |
| ACN39727.1 | SibD | BGC0000428 | Sibiromycin\_biosynthetic\_gene\_cluster | 33 | 24.036918138 | 248 | 7.7e-65 |
| ALV82447.1 | putative\_non-ribosomal\_peptide\_synthetase | BGC0001382 | Svaricin\_biosynthetic\_gene\_cluster | 33 | 23.9967897271 | 248 | 1e-64 |
| AGE11891.1 | nonribosomal\_peptide\_synthetase | BGC0000366 | Gobichelin\_biosynthetic\_gene\_cluster | 32 | 22.4719101124 | 247 | 1.7e-64 |
| ABC39418.1 | dihydroaeruginoic\_acid\_synthetase | BGC0000964 | Burkholdac\_biosynthetic\_gene\_cluster | 32 | 23.6356340289 | 247 | 1.7e-64 |
| ABA59547.1 | Vlm1 | BGC0000453 | Valinomycin\_biosynthetic\_gene\_cluster | 30 | 24.5585874799 | 247 | 2.2e-64 |
| CBJ89761.1 | Non-ribosomal\_peptide\_synthase\_involved\_in\_xenocoumacin\_synthesis | BGC0001054 | Xenocoumacin\_biosynthetic\_gene\_cluster | 29 | 24.518459069 | 246 | 3.8e-64 |
| CBW45692.1 | putative\_modular\_polyketide\_synthase\_(fragment) | BGC0000952\_c3 | Pristinamycin\_biosynthetic\_gene\_cluster | 32 | 21.0674157303 | 245 | 6.5e-64 |
| ACN39014.1 | putative\_nonribosomal\_peptide\_synthetase\_TomA | BGC0000448 | Tomaymycin\_biosynthetic\_gene\_cluster | 31 | 24.7191011236 | 245 | 8.5e-64 |
| ctg14\_orf2 |  | BGC0000095 | Micromonolactam\_biosynthetic\_gene\_cluster | 34 | 22.5521669342 | 244 | 1.4e-63 |
| CAD32904.2 | non-ribosomal\_peptide\_synthetase\_A | BGC0000354 | Friulimicin\_biosynthetic\_gene\_cluster | 33 | 23.3547351525 | 244 | 1.4e-63 |
| AAF67501.2 | NovH | BGC0000834 | Novobiocin\_biosynthetic\_gene\_cluster | 32 | 24.036918138 | 244 | 1.4e-63 |
| ABP57745.1 | DepA | BGC0000993 | FK228\_biosynthetic\_gene\_cluster | 33 | 23.113964687 | 243 | 1.9e-63 |
| ctg1\_orf00001 |  | BGC0000901 | Ferrichrome\_biosynthetic\_gene\_cluster | 29 | 26.0834670947 | 241 | 1.2e-62 |
| AGZ15476.1 | putative\_non\_ribosomal\_peptide\_synthetase | BGC0001036 | Polyoxypeptin\_biosynthetic\_gene\_cluster | 32 | 23.5553772071 | 238 | 6.1e-62 |
| AFK57218.1 | DidG | BGC0000985 | Didemnin\_biosynthetic\_gene\_cluster | 33 | 22.1107544141 | 237 | 1.8e-61 |
| ABP53496.1 | Erythronolide\_synthase | BGC0001041 | Salinosporamide\_biosynthetic\_gene\_cluster | 32 | 21.5489566613 | 235 | 5.2e-61 |
| ABP73647.1 | SalC | BGC0000145 | Salinosporamide\_A\_biosynthetic\_gene\_cluster | 32 | 21.5489566613 | 235 | 5.2e-61 |
| AMK48225.1 | nonribosomal\_peptide\_synthetase | BGC0001351 | Cahuitamycins\_biosynthetic\_gene\_cluster | 31 | 24.5585874799 | 235 | 6.7e-61 |
| CBL93720.1 | NRPS\_didomain\_PCP-A | BGC0000360 | GE81112\_biosynthetic\_gene\_cluster | 32 | 24.3980738363 | 235 | 8.8e-61 |
| AAS92545.1 | SirP | BGC0001044 | Sirodesmin\_biosynthetic\_gene\_cluster | 30 | 24.9598715891 | 235 | 8.8e-61 |
| AAG02343.1 | peptide\_synthetase\_NRPS12 | BGC0000963 | Bleomycin\_biosynthetic\_gene\_cluster | 34 | 24.1171749599 | 232 | 4.4e-60 |
| CAG23961.2 | polyketide\_synthase | BGC0001089 | Bacillaene\_biosynthetic\_gene\_cluster | 30 | 21.8298555377 | 230 | 1.7e-59 |
| BAO73252.1 | non-ribosomal\_peptide\_synthetase | BGC0000456 | Verlamelin\_biosynthetic\_gene\_cluster | 30 | 25.2407704655 | 230 | 2.2e-59 |
| CCE28989.1 | non-ribosomal\_peptide\_synthetase | BGC0001365 | Clapurines\_biosynthetic\_gene\_cluster | 31 | 24.1974317817 | 230 | 2.8e-59 |
| CBA11570.1 | non-ribosomal\_peptide\_synthetase | BGC0001046 | Streptolydigin\_biosynthetic\_gene\_cluster | 33 | 20.6661316212 | 230 | 2.8e-59 |
| AAN85506.1 | nonribosomal\_peptide\_synthetase | BGC0001101 | Leinamycin\_biosynthetic\_gene\_cluster | 32 | 23.4751203852 | 228 | 6.3e-59 |
| AFR69331.1 | nonribosomal\_peptide\_synthetase\_SpiA | BGC0001045 | Spiruchostatin\_biosynthetic\_gene\_cluster | 30 | 24.8394863563 | 228 | 8.2e-59 |
| CAF32362.1 | putative\_non-ribosomal\_peptide\_synthetase | BGC0000712 | Paromomycin\_biosynthetic\_gene\_cluster | 34 | 22.0706260032 | 228 | 1.1e-58 |
| CBW45640.1 | PII\_peptide\_synthetase | BGC0000952 | Pristinamycin\_biosynthetic\_gene\_cluster | 31 | 21.4686998395 | 228 | 1.1e-58 |
| EAL85113.2 | hybrid\_PKS-NRPS\_enzyme,\_putative | BGC0001037 | Pseurotin\_A\_biosynthetic\_gene\_cluster | 31 | 24.7592295345 | 226 | 3.1e-58 |
| AEA29643.1 | putative\_nonribosomal\_peptide\_synthetase | BGC0000409 | Porothramycin\_biosynthetic\_gene\_cluster | 30 | 23.3948635634 | 226 | 4.1e-58 |
| AGN74898.1 | nonribosomal\_peptide\_synthetase | BGC0000459 | Griseoviridin\_/\_viridogrisein\_biosynthetic\_gene\_cluster | 34 | 20.4654895666 | 225 | 9.1e-58 |
| ACN39728.1 | SibE | BGC0000428 | Sibiromycin\_biosynthetic\_gene\_cluster | 29 | 23.4349919743 | 225 | 9.1e-58 |
| AFT91378.1 | EcdA | BGC0000338 | Echinocandin\_B\_biosynthetic\_gene\_cluster | 30 | 24.8796147673 | 225 | 9.1e-58 |
| AJO72717.1 | adenylation\_domain-containing\_protein | BGC0001381 | Brasilinolide\_biosynthetic\_gene\_cluster | 31 | 23.1942215088 | 223 | 2e-57 |
| EPE34341.1 | Acetyl-CoA\_synthetase-like\_protein | BGC0001035 | Pneumocandin\_biosynthetic\_gene\_cluster | 29 | 27.808988764 | 223 | 3.5e-57 |
| CBW54673.1 | ketosynthase | BGC0000971 | Cinnabaramide\_biosynthetic\_gene\_cluster | 31 | 20.6260032103 | 220 | 2.2e-56 |
| ACG60778.1 | NRPS(A/PCP) | BGC0001058 | Zorbamycin\_biosynthetic\_gene\_cluster | 30 | 23.3146067416 | 218 | 6.5e-56 |
| AMM63162.1 | AniA | BGC0001371 | Echinocandin\_B\_biosynthetic\_gene\_cluster | 30 | 24.7993579454 | 218 | 8.5e-56 |
| CDF96614.1 | Long-chain-fatty-acid--CoA\_ligase | BGC0001149 | Brabantamide\_biosynthetic\_gene\_cluster | 30 | 24.3178170144 | 217 | 1.5e-55 |
| AFK57215.1 | DidD | BGC0000985 | Didemnin\_biosynthetic\_gene\_cluster | 30 | 24.2776886035 | 217 | 1.9e-55 |
| AAO39107.1 | AdmM | BGC0000956 | Andrimid\_biosynthetic\_gene\_cluster | 30 | 21.4686998395 | 215 | 5.5e-55 |
| AEA29644.1 | putative\_nonribosomal\_peptide\_synthetase\_and\_kinurenine\_monooxygenase | BGC0000409 | Porothramycin\_biosynthetic\_gene\_cluster | 30 | 25.7624398074 | 214 | 1.2e-54 |
| WP\_051700151.1 | hypothetical\_protein | BGC0001368 | Tambromycin\_biosynthetic\_gene\_cluster | 33 | 22.7126805778 | 214 | 1.6e-54 |
| CCP45171.1 | Phenyloxazoline\_synthase\_MbtB\_(phenyloxazoline\_synthetase) | BGC0001021 | Mycobactin\_biosynthetic\_gene\_cluster | 29 | 24.3579454254 | 213 | 2.1e-54 |
| ADZ45333.1 | NRPS/PKS\_hybrid | BGC0001020 | Muraymycin\_biosynthetic\_gene\_cluster | 30 | 23.595505618 | 210 | 1.8e-53 |
| AFK57220.1 | DidI | BGC0000985 | Didemnin\_biosynthetic\_gene\_cluster | 33 | 22.2311396469 | 210 | 2.3e-53 |
| CCJ67647.1 | JagC | BGC0001127 | Jagaricin\_biosynthetic\_gene\_cluster | 32 | 22.5521669342 | 209 | 4e-53 |
| BAN91483.1 | putative\_Aculeacin\_A\_nonribosomal\_peptide\_synthase | BGC0001220 | Aculeacin\_A\_biosynthetic\_gene\_cluster | 28 | 27.2471910112 | 209 | 5.2e-53 |
| BAP82667.1 | non-ribosomal\_peptide\_synthetase\_A-domain\_containing\_protein | BGC0001148 | Pheganomycin\_biosynthetic\_gene\_cluster | 32 | 20.1444622793 | 209 | 5.2e-53 |
| AHB38511.1 | EpnJ | BGC0000345 | Eponemycin\_biosynthetic\_gene\_cluster | 29 | 23.7158908507 | 208 | 1.2e-52 |
| CAJ76286.1 | putative\_non-ribosomal\_peptide\_synthetase | BGC0000972 | Colibactin\_biosynthetic\_gene\_cluster | 28 | 23.7158908507 | 207 | 1.5e-52 |
| ADY76684.1 | non-ribosomal\_peptide\_synthetase | BGC0000950 | Napsamycin\_biosynthetic\_gene\_cluster | 32 | 20.0642054575 | 207 | 2e-52 |
| AEH42484.1 | adenylation\_for\_L-proline | BGC0000032 | Calcimycin\_biosynthetic\_gene\_cluster | 32 | 20.746388443 | 206 | 4.4e-52 |
| AAF01762.1 | AM-toxin\_synthetase | BGC0001261 | AM-toxin\_biosynthetic\_gene\_cluster | 27 | 24.9197431782 | 205 | 9.7e-52 |
| AFK57214.1 | DidC | BGC0000985 | Didemnin\_biosynthetic\_gene\_cluster | 33 | 22.8731942215 | 205 | 9.7e-52 |
| ADZ45339.1 | non-ribosomal\_peptide\_synthetase | BGC0001020 | Muraymycin\_biosynthetic\_gene\_cluster | 31 | 20.5056179775 | 204 | 1.7e-51 |
| EFY94500.1 | non-ribosomal\_peptide\_synthetase | BGC0000337 | Destruxins\_biosynthetic\_gene\_cluster | 28 | 24.1974317817 | 203 | 3.7e-51 |
| ADN13832.1 | Beta-ketoacyl\_synthase | BGC0001164 | 1-heptadecene\_biosynthetic\_gene\_cluster | 29 | 20.5056179775 | 200 | 2.4e-50 |
| ctg1\_orf000000 |  | BGC0000900 | Ferrichrome\_biosynthetic\_gene\_cluster | 28 | 25.0802568218 | 194 | 1.7e-48 |
| CBF87069.1 | nonribosomal\_peptide\_synthase,\_putative\_(Eurofung) | BGC0001290 | Emericellamide\_A\_/\_emericellamide\_B\_biosynthetic\_gene\_cluster | 28 | 24.1573033708 | 197 | 1.6e-49 |
| AAG31130.1 | MxcG | BGC0001345 | Myxochelin\_biosynthetic\_gene\_cluster | 29 | 24.3579454254 | 196 | 4.5e-49 |
| ACN69986.1 | proline\_adenyltransferase | BGC0000079 | Indanomycin\_biosynthetic\_gene\_cluster | 32 | 21.5088282504 | 191 | 8.5e-48 |
| AIC32695.1 | FR9I | BGC0001113 | Spliceostatin\_/\_FR901464\_biosynthetic\_gene\_cluster | 29 | 25.7624398074 | 187 | 1.6e-46 |
| AAD24881.1 | putative\_acyl-CoA\_synthetase | BGC0000127 | Pyoluteorin\_biosynthetic\_gene\_cluster | 29 | 20.6661316212 | 187 | 1.6e-46 |
| ADH01490.1 | type\_I\_polyketide\_synthase | BGC0001096 | FR901464\_biosynthetic\_gene\_cluster | 29 | 25.7624398074 | 187 | 1.6e-46 |
| CBL93716.1 | NRPS\_A-domain | BGC0000360 | GE81112\_biosynthetic\_gene\_cluster | 31 | 20.9069020867 | 187 | 2.1e-46 |
| ACR78148.1 | BSLS | BGC0000312 | Bassianolide\_biosynthetic\_gene\_cluster | 27 | 24.3980738363 | 186 | 2.7e-46 |
| AAG29789.1 | acyl-CoA\_synthetase | BGC0000833 | Coumermycin\_A1\_biosynthetic\_gene\_cluster | 31 | 21.0674157303 | 186 | 2.7e-46 |
| AAN65233.1 | acyl-CoA\_synthetase | BGC0000832 | Clorobiocin\_biosynthetic\_gene\_cluster | 30 | 20.9871589085 | 185 | 6.1e-46 |
| AHA34037.1 | Bmp4 | BGC0000890 | Bromophenols\_/\_bromopyrroles\_biosynthetic\_gene\_cluster | 29 | 20.9069020867 | 183 | 4e-45 |
| EAL89049.1 | nonribosomal\_peptide\_synthase,\_putative | BGC0000355 | Fumiquinazolines\_biosynthetic\_gene\_cluster | 27 | 25.8828250401 | 183 | 4e-45 |
| CAA16182.1 | putative\_peptide\_synthase | BGC0001063 | Undecylprodigiosin\_biosynthetic\_gene\_cluster | 30 | 21.4686998395 | 181 | 8.8e-45 |
| AHF22853.1 | MarM | BGC0000091 | Marineosin\_biosynthetic\_gene\_cluster | 30 | 21.2680577849 | 180 | 2e-44 |
| CCB53264.1 | non-ribosomal\_peptide\_synthetase | BGC0001393 | Lugdunin\_biosynthetic\_gene\_cluster | 25 | 22.8330658106 | 180 | 3.4e-44 |
| AHA12086.1 | amino\_acid\_adenyltransferase | BGC0001172 | Chlorizidine\_A\_biosynthetic\_gene\_cluster | 28 | 20.746388443 | 176 | 2.8e-43 |
| EAW16180.1 | nonribosomal\_peptide\_synthase,\_putative | BGC0000293 | Acetylaszonalenin\_biosynthetic\_gene\_cluster | 27 | 24.7191011236 | 176 | 3.7e-43 |
| CCA54209.1 | non-ribosomal\_peptide\_synthetase | BGC0000893 | Chloramphenicol\_biosynthetic\_gene\_cluster | 26 | 22.7126805778 | 175 | 6.3e-43 |
| CAH55637.1 | putative\_L-prolyl-AMP\_ligase | BGC0000258 | Prodigiosin\_biosynthetic\_gene\_cluster | 31 | 20.5858747994 | 173 | 2.4e-42 |
| ESP90847.1 | amino\_acid\_adenylation\_domain\_protein | BGC0000891 | Bromophenols\_/\_bromopyrroles\_biosynthetic\_gene\_cluster | 28 | 20.6260032103 | 172 | 7e-42 |
| AGC83576.1 | NRPS | BGC0000818 | Notoamide\_biosynthetic\_gene\_cluster | 26 | 23.9566613162 | 169 | 4.5e-41 |
| BAG84247.1 | putative\_L-prolyl-AMP\_ligase | BGC0000257 | Prodigiosin\_biosynthetic\_gene\_cluster | 30 | 20.1043338684 | 166 | 3.8e-40 |
| AET13879.1 | epichloenin\_A\_synthetase | BGC0001251 | Epichloenin\_A\_biosynthetic\_gene\_cluster | 27 | 24.5987158909 | 164 | 1.5e-39 |
| AET13875.1 | epichloenin\_A\_synthetase | BGC0001250 | Epichloenin\_A\_biosynthetic\_gene\_cluster | 27 | 24.5987158909 | 163 | 4.2e-39 |
| CAH55654.1 | putative\_L-prolyl-AMP\_ligase | BGC0000259 | Prodigiosin\_biosynthetic\_gene\_cluster | 31 | 20.1444622793 | 162 | 5.5e-39 |
| AAA33023.2 | HC-toxin\_synthetase | BGC0000370 | HC-toxin\_biosynthetic\_gene\_cluster | 27 | 21.5088282504 | 162 | 7.2e-39 |
| AAG02365.1 | peptide\_synthetase\_NRPS0 | BGC0000963 | Bleomycin\_biosynthetic\_gene\_cluster | 26 | 24.5585874799 | 160 | 3.6e-38 |
| XP\_001227984.1 | hypothetical\_protein | BGC0001240 | Serinocyclin\_A\_/\_serinocyclin\_B\_biosynthetic\_gene\_cluster | 27 | 24.3980738363 | 159 | 6.1e-38 |
| ACJ04424.1 | aureobasidin\_A1\_biosynthesis\_complex | BGC0000307 | Aureobasidin\_A1\_biosynthetic\_gene\_cluster | 25 | 23.9967897271 | 157 | 1.8e-37 |
| ADM34138.1 | non-ribosomal\_peptide\_synthetase | BGC0001084 | Notoamide\_/\_stephacidin\_biosynthetic\_gene\_cluster | 25 | 23.9967897271 | 156 | 5.2e-37 |
| CCB53267.1 | non-ribosomal\_peptide\_synthetase | BGC0001393 | Lugdunin\_biosynthetic\_gene\_cluster | 23 | 23.4751203852 | 148 | 1.1e-34 |
| ABX71145.1 | Lcz28 | BGC0000237 | Lactonamycin\_Z\_biosynthetic\_gene\_cluster | 28 | 21.8298555377 | 148 | 1.4e-34 |
| BAR73011.1 | putative\_ATP-dependent\_aminoacyl-ACP\_synthetase | BGC0001194 | Hitachimycin\_biosynthetic\_gene\_cluster | 27 | 20.1444622793 | 144 | 2e-33 |
| BAQ25507.1 | type\_I\_polyketide\_synthase | BGC0001288 | Maklamicin\_biosynthetic\_gene\_cluster | 28 | 22.7528089888 | 143 | 3.5e-33 |
| AAO39105.1 | AdmK | BGC0000956 | Andrimid\_biosynthetic\_gene\_cluster | 24 | 22.9133226324 | 143 | 3.5e-33 |
| CDG76957.1 | non-ribosomal\_peptide\_synthetase | BGC0000446 | Tilivalline\_biosynthetic\_gene\_cluster | 26 | 20.1444622793 | 142 | 5.9e-33 |
| BAD08370.1 | non-ribosomal\_peptide\_synthetase | BGC0000167 | Vicenistatin\_biosynthetic\_gene\_cluster | 27 | 20.9069020867 | 139 | 6.6e-32 |
| AJV88383.1 | MfnK | BGC0001214 | Marformycins\_biosynthetic\_gene\_cluster | 28 | 21.8699839486 | 138 | 8.6e-32 |
| AAO39104.1 | AdmJ | BGC0000956 | Andrimid\_biosynthetic\_gene\_cluster | 27 | 20.5056179775 | 138 | 8.6e-32 |
| AIL50189.1 | putative\_5-aminolevulinate\_CoA-ligase | BGC0000213 | Colabomycin\_biosynthetic\_gene\_cluster | 26 | 20.5056179775 | 131 | 1.4e-29 |
| ACO94464.1 | NRPS\_adenylation\_domain\_protein | BGC0000029 | BE-14106\_biosynthetic\_gene\_cluster | 28 | 20.7865168539 | 130 | 3e-29 |
| BAO66530.1 | nonribosomal\_peptide\_synthase | BGC0000042 | Cremimycin\_biosynthetic\_gene\_cluster | 25 | 20.7062600321 | 126 | 3.4e-28 |
| AGI61651.1 | long\_chain\_fatty\_acid\_CoA\_ligase | BGC0000095 | Micromonolactam\_biosynthetic\_gene\_cluster | 28 | 20.3852327448 | 126 | 5.7e-28 |
| ABX71111.1 | Lct28 | BGC0000238 | Lactonamycin\_biosynthetic\_gene\_cluster | 24 | 21.9101123596 | 118 | 1.6e-25 |
| BAP34718.1 | AMP-dependent\_synthetase\_and\_ligase | BGC0000078 | Incednine\_biosynthetic\_gene\_cluster | 27 | 21.1878009631 | 115 | 1e-24 |
| AEA60663.1 | pyoverdine\_synthetase | BGC0000892 | Caryoynencin\_biosynthetic\_gene\_cluster | 24 | 22.7126805778 | 79 | 4.7e-14 |
| WP\_052390333.1 | hypothetical\_protein | BGC0001308\_c2 | Simocyclinone\_D9\_/\_simocyclinone\_D10\_/\_simocyclinone\_D11 | 23 | 20.9069020867 | 69 | 8.3e-11 |
